# Grain boundary engineered aluminum current collector for energy-dense initially anode-free sodium metal batteries

**DOI:** 10.1038/s41467-026-74177-8

**Published:** 2026-06-06

**Authors:** Xueying Zheng, Dongpeng Yu, Fei Tian, Danni Lei, Chengxin Wang

**Affiliations:** State Key Laboratory of Optoelectronic Materials and Technologies, School of Materials Science and Engineering, Sun Yat-sen (Zhongshan) University, Guangzhou, China

**Keywords:** Batteries, Batteries, Energy, Batteries

## Abstract

The initially anode-free sodium metal batteries represent promising candidates for high specific energy and safe battery systems, relying solely on cathodic sodium reservoirs. However, the irreversible accumulation of electrochemically inactive “dead” Na and unstable solid electrolyte interphases fundamentally constrains cyclability through rapid active Na depletion. Here, we utilize a grain-boundary gallium-rich polycrystalline aluminum current collector to trigger controlled dissolution of aluminum ions. The dissolved aluminum ions modify the local coordination environment of the electrolyte, thereby perturbing the solvation equilibrium of sodium ions and facilitating sodium ions migration toward the positive electrode for charge compensation. The gallium-rich aluminum current collector enables stable Na plating/stripping for over 2500 cycles. The pouch cell with Na_3_V_2_(PO_4_)_3_ delivers 88.7% capacity retention after 100 cycles at 35.1 mA g^−1^ in 1 M NaPF_6_ in diglyme, based on a nominal capacity of 117 mAh g^−1^. In addition, the full cell with a high positive electrode loading (47.4 mg cm^−2^) achieves a high specific energy of 201.5 Wh kg^−1^, calculated based on all cell components (positive electrode, negative electrode, separator and electrolyte). This work proposes a viable current collector design concept that can be extended to other initially anode-free battery systems.

## Abstract (web summary)

The initially anode-free sodium metal offer advantages in energy density and safety. Here, authors design a grain-boundary Ga-rich polycrystalline aluminum current collector that enables controlled Al3+ dissolution to compensate Na loss, stabilizes Na plating/stripping, and delivers reversible batteries.

## Introduction

The growing demand for rechargeable batteries necessitates the development of novel battery systems to complement or replace lithium-ion technology^1–3^. Current sodium-ion batteries (SIBs) deliver relatively limited energy densities(105–150 Wh kg^−1^), are insufficient for long-range applications^4,5^. While recent efforts to enhance specific energy via negative electrode optimization have made progress, typical carbon materials like hard carbon are constrained by relatively low theoretical specific capacity of approximately 370 mAh g^−1^ and limited sodium storage sites^6,7^. In contrast, sodium metal (Na) is an attractive negative electrode candidate due to its high theoretical capacity (1166 mAh g^−1^) and a low electrode potential (−2.71 V vs standard hydrogen electrode, SHE)^8,9^. Its plating/stripping chemistry provides a direct route to higher specific energy, without relying on intercalation mechanisms^10^. However, the inherent air instability of Na presents significant challenges for practical applications^11^. The initially anode-free sodium metal battery (AFSMB) concept addresses these challenges by utilizing only a current collector as the initial negative electrode, thereby maximizing specific energy and enhancing safety^12,13^.

During charging in an AFSMB, sodium ions (Na^+^) are extracted from the positive electrode and reduced in situ onto the negative electrode current collector, forming the Na metal negative electrode^14^. However, Na deposition tends to initiate tip growth, leading to the formation of loose dendritic structures^15^. Concurrently, the high reactivity of Na drives continuous electrolyte decomposition and solid electrolyte interphase (SEI) formation^16^. Upon discharge, these dendritic structures may detach, becoming electrochemically isolated “dead Na”. This irreversibility depletes active Na^+^ inventory, returning fewer ions to the positive electrode than were extracted during charging. Consequently, Na^+^ deficiency triggers the rapid degradation of the positive electrode crystal structure, accelerating capacity fade and cycle life deterioration. Strategies such as positive electrode pre-sodiation can replenish lost Na, but often induce irreversible material lattice distortion and exacerbate interfacial side reactions at the positive electrode^17^. Similarly, modifying the current collector with sodiophilic phases improves the reversibility of Na but fails to enable Na compensation^18–20^. Therefore, achieving simultaneous control over dendrite-free Na deposition and in situ self-replenishment of active Na remains a critical challenge for practical AFSMBs.

Here, we fabricate a grain-boundary gallium-rich polycrystalline aluminum current collector (AlGa) by directly coating gallium (Ga) onto Al foil. Ga penetrates into the grain boundaries of the Al foil at 30 °C, inducing the controlled dissolution of Al^3+^. The dissolved Al^3+^ can interact with diglyme molecules, potentially altering the Na^+^ solvation environment and facilitating desolvation. Meanwhile, the dissolution process releases electrons into the external circuit, which are coupled with Na^+^ insertion at the positive electrode. This process may drive Na^+^ migration and contribute to compensating Na loss. The Na||AlGa cell exhibits a low nucleation overpotential and enables stable plating/stripping for 2500 cycles at a capacity of 1 mAh cm^−2^ and a current density of 1 mA cm^−2^. Pairing AlGa with commercial Na_3_V_2_(PO_4_)_3_ (NVP; 11.4 mg cm^−2^, N/P = 0) allows stable cycling at 35.1 mA g^−1^ with 88.7% capacity retention after 100 cycles. Furthermore, to achieve higher specific energy (201.5 Wh kg^−1^), AlGa is paired with a high loading NVP (47.4 mg cm^−2^). This work provides a feasible strategy for developing initially anode-free sodium metal batteries and may be extended to other initially anode-free battery systems.

## Results

### Mechanism of AlGa current collector

**Fig. 1:**
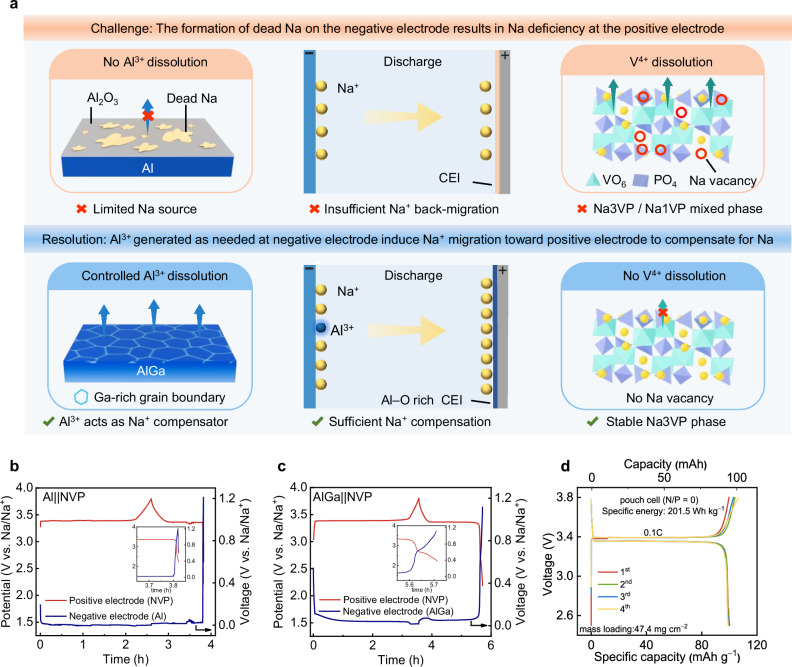
Mechanism of AlGa current collector. **a** Design ideas for the current collector of initially anode-free sodium metal battery. **b**, **c** Electrochemical behavior curves of Al||NVP and AlGa||NVP during charge-discharge cycles in Swagelok cell. The potential curve of AlGa and Al relative to Na were obtained through additional auxiliary measurements. **d** The voltage-capacity curves of 201.5 Wh kg^−1^ AlGa||NVP pouch cell. The specific parameters of the soft packaging in this figure can be found in Supplementary Information, Table 2.

For a typical charging process of the Na battery, the Na_3_V_2_(PO_4_)_3_ (Na3VP) releases Na^+^ to form the NaV_2_(PO_4_)_3_ (Na1VP), accompanied by the oxidation of V^3+^ to V^4+^. In the absence of Na deficiency, the discharge process entails Na^+^ reinsertion into Na1VP, reducing it back to Na3VP. Fig. 1a schematically illustrates the discharge states of Al | |NVP and AlGa | |NVP cells. For Al collectors, due to uneven Na deposition, the Na with dendritic structures is prone to detachment, becoming electrochemically isolated “dead Na”, while the inherent Al_2_O_3_ layer hinders the release of Al^3+^ in the ether electrolyte (1 mol L^−1^ NaPF_6_ in diglyme)^21^. Consequently, only a fraction of Na^+^ reintercalates into the positive electrode during discharge, resulting in a mixed phase composed of both Na3VP and Na1VP. Although both phases share the NASICON-type framework, the Na⁺ in Na1VP occupy only partial lattice sites, resulting in a higher vanadium oxidation state and lattice contraction, making the framework more susceptible to phase degradation and V^4+^ dissolution. For AlGa, the sodiophilic nature of Ga effectively alleviates the accumulation of electrochemically inactive Na, but irreversible Na^+^ consumption remains due to continuous SEI formation. Owing to its strong affinity for Al, Ga spontaneously diffuses into Al grain boundaries and lattice sites, disrupting the native Al_2_O_3_ layer and inhibiting new oxide formation^22^. Furthermore, Ga penetration along the Al grain boundaries leads to Ga enrichment and partial Al–Ga alloying. This grain-boundary modification weakens the local Al bonding environment and facilitates the gradual dissolution of Al atoms. In the discharge state, when Al^3+^ dissolves, electrons are released into the external circuit and transferred to the positive electrode, which prompts Na^+^ migration from the electrolyte toward the positive electrode. In addition, the dissolution of Al^3+^ into the electrolyte leads to its interaction with diglyme molecules (Supplementary Fig. 1), which may modify the Na^+^ solvation structure by altering the coordination environment between diglyme and Na^+^. This change in solvation equilibrium could facilitate the dissociation of coordinated Na⁺. This promotes more efficient utilization of Na^+^ in the electrolyte and effectively converts back to Na3VP. Besides, the dissolved Al^3+^ cannot be redeposited due to its high charge density and strong solvation. Instead, the dissolved Al^3+^ preferentially migrates toward the positive electrode and incorporate into the positive electrode–electrolyte interphase (CEI) as Al–O moieties. This CEI configuration reinforces interfacial integrity, ultimately improving cell cyclability.

The electrochemical processes on the Al and AlGa surfaces were further investigated using a three-electrode system (Fig. 1b–c). During cycling, current was applied to Al||NVP and AlGa||NVP. The cutoff voltages were 3.8 V (charge) and 2.2 V (discharge). The voltages between Al||NVP and Na||Al were monitored. During the charging process, Na^+^ was deposited on Al or AlGa, with the potential relative to Na approaching 0 V. During discharge, the voltage stayed near 0 V, whereas distinct electrochemical behavior is observed in the later stage of discharge. The standard potential of Al^3+^/Al (−1.662 V vs. SHE) is 1.05 V higher than that of Na⁺/Na (−2.71 V), thermodynamically allowing Al oxidation at *E* > 1.05 V vs. Na^+^/Na. However, the spontaneously formed Al_2_O_3_ passivation layer imposes an activation barrier for dissolution, resulting in apparent electrochemical inertness. At the end of discharge, the voltage between Al and Na rapidly rises to 1.2 V and then decreases, indicating that Al^3+^ dissolution is suppressed. For AlGa, the negative shift in corrosion potential (ΔE = 0.3–0.5 V) of Al–Ga alloys relative to pure Al^23,24^ reduces the activation barrier for anodic dissolution. Consequently, the voltage between AlGa and Na exhibits a distinct slope between 0.6 and 1.0 V vs. Na⁺/Na, corresponding to the initial stage of aluminum ionization (Al → Al^3+^ + 3e^−^). Notably, due to the relatively poor sealing capability of the Swagelok cell, the deposited Na on the Al and AlGa current collectors may be partially oxidized, which could exacerbate the irreversible Na⁺ loss. However, this does not affect the observation of Al dissolution.

In order to visualize the actual occurrence of Al^3+^ dissolution, we used aurintricarboxylic acid triammonium salt as an Al^3+^ indicator (Supplementary Fig. 2, Al reagent, 0.5 g L^−1^ in ultrapure water). The Al reagent complexs with Al^3+^, causing the solution color to change from orange to red. Further, ultraviolet and visible spectroscopy (UV–Vis) confirms the origin of the color change, showing a distinct peak near 533 nm, corresponding to the complexation of Al^3+^ and Al reagents (Supplementary Fig. 3)^25^. Polypropylene (PP) separators after different numbers of cycles in Na||AlGa and Na||Al cells were removed and immersed in Al reagent solutions (Supplementary Fig. 4a). Remarkably, even minimal electrolyte infiltration (μL scale) into individual PP pieces resulted in significant Al^3+^ leaching. Moreover, the color changes to a deeper red color as the number of cycles increases, indicating the gradual Al^3+^ dissolution with increasing cycle numbers. In contrast to Na | |Al, no evidence of Al^3+^ dissolution is observed even after 200 cycles. The UV–Vis results are consistent with the above conclusions (Supplementary Fig. 4b). To quantitatively evaluate this effect, as shown in Supplementary Table 1, the Al concentration in the electrolyte after long cycling was measured to be ~0.013 mol L^−1^ by Inductively Coupled Plasma–Optical Emission Spectrometry (ICP-OES). The dissolved Al^3+^ may also play a beneficial role by contributing to charge compensation and modifying the solvation structure, thereby influencing SEI and CEI formation. A proof-of-concept pouch cell with a high specific energy of 201.5 Wh kg^−1^ (NVP loading of 47.4 mg cm^−2^) was successfully operated (Fig. 1d and Supplementary Fig. 5), demonstrating the practical potential of AFSMBs. The specific energy of the full cell was calculated for all parameters except the packaging material (Supplementary Table 2).

### Fabrication and characterization of AlGa

**Fig. 2:**
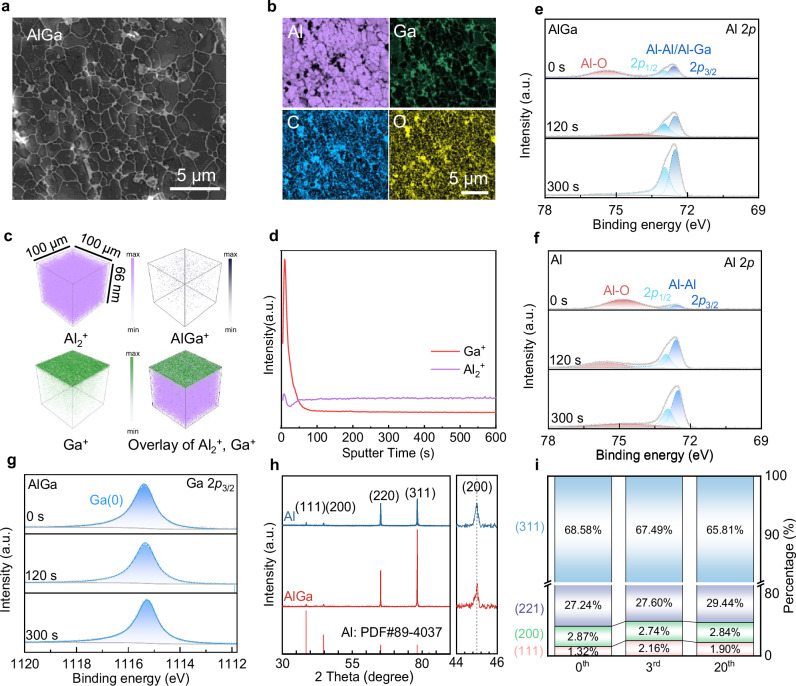
Fabrication and characterization of AlGa. **a** SEM image of the AlGa. **b** EDS mappings of AlGa. **c** TOF-SIMS 3D reconstruction of the sputtered volume of several secondary ion fragments on the AlGa (**d**) The variation of fragment species intensity with sputter time. XPS spectra of Al 2*p* for the surface of AlGa (**e**) and Al (**f**). **g** XPS spectra of Ga 2*p*_3/2_. **h** XRD patterns of AlGa and Al foil. **i** Changes in the relative peak area ratios of XRD patterns after AlGa cycling.

The AlGa was prepared by uniformly brushing liquid Ga onto an Al foil (Supplementary Fig. 6a). After aging at 30 °C for more than 24 h, Ga preferentially diffuses along Al grain boundaries and subsequently migrates into the Al lattice^26,27^. This scalable method enables large-area fabrication. Top-view scanning electron microscopy (SEM) images and energy-dispersive spectroscopy (EDS) elemental mapping (Fig. 2a, b and Supplementary Fig. 6b, c) show the enrichment of Ga at the grain boundaries as well as partial Al–Ga solid solutions. Ga adsorbs organic solvent molecules, resulting in the simultaneous observation of Ga, C, and O in the SEM^28,29^. Owing to the presence of Ga at the grain boundaries, a reduction in hardness and electrical conductivity is observed for AlGa^30,31^ (Supplementary Tables 3 and 4). Nevertheless, despite the decrease in hardness and conductivity compared with pristine Al, the resulting values (0.31 GPa in hardness and 2.12 × 10^7^ S m^−1^ in conductivity) remain sufficient for current collector applications. The unmodified Al foil surface shows visible gaps and cracks (Supplementary Fig. 7). The Time-of-Flight Secondary Ion Mass Spectrometry (TOF-SIMS) 3D reconstruction image shows that Ga is uniformly dispersed on the Al surface, with Ga^+^ and AlGa^+^ fragments detected, proving that Ga exists simultaneously at the grain boundaries and in the Al–Ga solid solution (Fig. 2c). The thickness is about 11.1 nm (Fig. 2d, Supplementary Fig. 8). X-ray photoelectron spectroscopy (XPS) was used to verify the damage to the Ga to the oxide layer on the Al surface. For Al 2*p* spectra (Fig. 2e, f), the peaks at 75.5 eV and 72.6 eV are attributed to Al–O and Al–Al/Al–Ga, respectively. Surface characterization reveals a distinct oxide layer on the pristine Al. In contrast, the AlGa sample exhibits a significantly reduced oxide layer, as Ga exhibits corrosive behavior towards Al_2_O_3_, leading to the destruction of the dense oxide layer. Following 300 s of Ar^+^ etching, the AlGa surface showed virtually no detectable oxide layer, whereas the Al sample retained trace amounts of surface oxides. The Ga 2*p*_*3/2*_ spectra display one component (Fig. 2g), with a peak at 1116.2 eV assigned to Ga metal. Fig. 2h shows that the X-ray diffraction (XRD) pattern of AlGa is similar to that of Al since no new phases were generated^32,33^. The (200) plane shows a peak with a slightly smaller angle, which can be attributed to the entry of some large Ga into the Al lattice to form an Al–Ga solid solution. The (111) and (200) planes show a decrease in intensity, whereas the (220) and (311) planes show a significant increase in intensity, which probably arises from the formation of a partial Al–Ga solid solution and the associated crystallographic reorientation.

To further illustrate why Ga only needs to accumulate mostly at grain boundaries, we prepared homogeneous Al–Ga solid solution samples (Supplementary Fig. 9, H-AlGa) and with surfaces full of Ga (Supplementary Fig. 10, S-AlGa). The PP separator cycled for 200 cycles in AlGa, S-AlGa and H-AlGa half-cells were immersed in an Al reagent solution (Supplementary Figs. 11 and 12). No Al leaching was observed for H-AlGa, limited leaching for S-AlGa, and the most pronounced leaching for AlGa, which proves that the enrichment of Ga at grain boundaries facilitates the dissolution of Al^3+^. The UV–Vis spectra showed the distinct peak near 533 nm for AlGa, further confirming enhanced Al dissolution. For S-AlGa, even though a large amount of Ga is on the surface of Al, the formation of Na–Ga alloy consumes most of the Ga and part of Na^+^ due to the incomplete reversibility of Na–Ga alloying/dealloying (Supplementary Fig. 13), which is inconsistent with our design principle. The Ga enriched in grain boundaries in the AlGa enables microalloying reaction while enhancing Al dissolution kinetics. According to the Al–Ga binary phase diagram^34^, the melting point of gallium (29.8 °C) is significantly lower than that of aluminum (660.4 °C). The melting point of the Al–Ga eutectic mixtures formed in the grain boundaries (~26.6 °C) is much lower than the bulk melting point of Al, which means that migration-dissolution processes can easily occur^35,36^. Additionally, since Ga partially enters the lattice of Al, it lowers the lattice energy of Al, which may also favor Al dissolution. XRD characterization (Fig. 2i, Supplementary Fig. 14) of AlGa after 3 and 20 cycles revealed small changes in the strength of each crystal surface during the cycling process, suggesting that Al^3+^ dissolution may induce slight perturbations in the Al lattice.

### Investigation of Na plating/stripping behavior

**Fig. 3:**
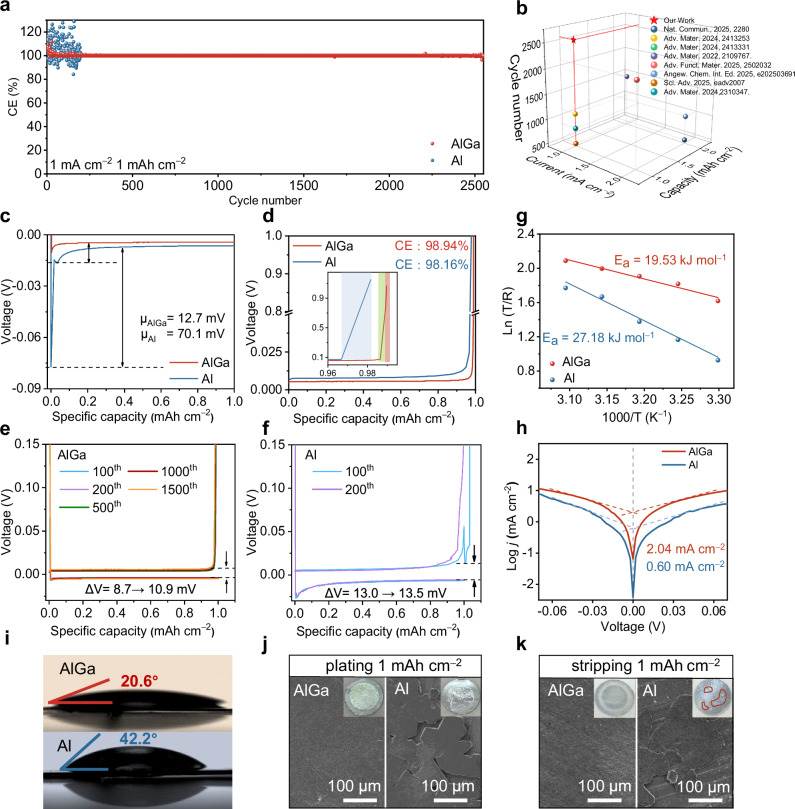
Electrochemical performance of the half and symmetric cells. **a** Coulombic efficiency of Na plating/ stripping on the AlGa and Al foil at 1 mAh cm^−2^ and 1 mA cm^−2^. **b** Comparison of the cycling performance of asymmetric cells between recently reported works and this work. The source of the literature data shown in this figure can be found in Supplementary Information, Table 5. The galvanostatic potential versus capacity curves of Na deposition (**c**) and stripping (**d**) at 1 mA cm^−2^ with 1 mAh cm^−2^. The blue shaded region indicates the gradual stripping of underlying Na on Al, while the green shaded region represents the extended Na stripping on the AlGa current collector. The red shaded region corresponds to the subsequent steep voltage increase, associated with limited Al stripping. **e**, **f** The galvanostatic Na deposition/stripping curve at different cycles. **g** Activation energy of Na^+^ diffusion between the electrolyte and electrodes. **h** Tafel curves of AlGa/Na and Al/Na symmetric cells. **i** Contact angles of electrolyte (1 M NaPF_6_ in diglyme) on AlGa and Al. **j** The SEM images of Na deposition AlGa and Al at 1 mA cm^−2^ for 1 mAh cm ^−2^, with corresponding optical images in the upper right corner. **k** The SEM images of AlGa and Al after stripping Na for 1 mAh cm ^−2^, with corresponding optical images in the upper right corner.

Even with Al^3+^ leaching compensating for the Na source, enhancing Na reversibility remains critical for cycling performance. In order to investigate the effect of the AlGa collector on the Na reversibility, half cells were assembled with Na as the counter electrode. AlGa shows high cycling stability and maintains stable plating/stripping over 2500 cycles at 1 mA cm^−2^ and 1 mAh cm^−2^, whereas Al shows unstable plating and stripping behavior (Fig. 3a). The results highlight the high Na reversibility of AlGa, which is competitive with recent reports^13,20,37–43^ (Fig. 3b and Supplementary Table 5). The initial nucleation behavior of Na was further investigated. AlGa exhibits a significantly lower nucleation overpotential (12.7 mV) compared to Al (70.1 mV), along with a smoother deposition profile (Fig. 3c). This can be attributed to the Na-philic nature of Ga, so the AlGa interfacial layer helps reduce the heterogeneous nucleation barrier of Na^+^^44^. Fig. 3d shows that in the first stripping curve, AlGa exhibits smaller polarization and higher coulombic efficiency (CE) compared to Al (98.94% vs. 98.16%). For Al collectors, in the initial stage, the nearly flat slope (close to zero) indicates the dominant stripping of Na⁺. As the capacity reaches 0.967 mAh cm^−2^, the increasing slope (blue shaded region) suggests the gradual stripping of the underlying Na under applied voltage and a CE of 98.16% is finally obtained. In contrast, the slope of AlGa remains low until the capacity approaches 0.986 mAh cm^−2^, indicating that a larger fraction of Na^+^ undergoes more efficient stripping (green region). The subsequent steep slope increase (red region) likely corresponds to limited Al stripping. As Al^3+^ removal requires higher energy, this stage may exhibit a sharp voltage rise per unit capacity. The voltage hysteresis from the 100^th^ to the 1500^th^ cycle shows only a very small increase (Δ*V* = 8.7 → 10.9 mV) in Fig. 3e, further demonstrating that Na on the AlGa collector is highly reversible. In contrast, the Al collector exhibits a larger polarization voltage and a more fluctuating stripping profile from 100th to 200th cycle (Fig. 3f). The CV curves (Supplementary Fig. 15) further confirm that AlGa has a stronger response current density and a lower deposition overpotential. In addition, a weak cathodic peak at 0.62 V corresponding to Na–Ga alloying and anodic peaks at 0.71 and 0.78 V, which are related to Al dissolution and Na–Ga dealloying (vs. Na/Na⁺), respectively, are identified. In the AlGa collector, due to the low content of Ga (~0.023 mg cm^−2^) and the presence of some Al–Ga solid solution, the volume expansion from trace NaGa_4_ has minimal impact on the current collector of the AlGa. Nanoindentation results (Supplementary Table 3) show that the hardness of AlGa after cycling is comparable to that before cycling. The small change in hardness before and after cycling suggests that the overall mechanical state of the surface remains largely unchanged, implying that the impact of Na–Ga alloy-induced volume expansion on the current collector is limited. Furthermore, Rietveld refinement of AlGa before and after 20 cycles (Supplementary Fig. 16) reveals only minor changes in the lattice parameters of Al, indicating that the combined effects of Al dissolution and Na–Ga alloying-induced volume expansion exert a limited impact on the structural integrity of the AlGa collector.

The Na | |AlGa and Na | |Al half cells after 1 cycle were measured by electrochemical impedance spectroscopy (EIS) tests (Supplementary Fig. 17), and the impedance value of the cell using Al (120 Ω) was much larger than that of AlGa (51 Ω). The initial nucleation behavior significantly influences the subsequent morphology of deposited Na^45,46^. Thus, the SEI formed between the deposited Na and the electrolyte, as well as the electronic contact between Na and the underlying current collector, can vary greatly depending on the surface properties of different current collectors. Therefore, 1 mAh cm^−2^ of Na was deposited on the collector (AlGa/Na and Al/Na) and assembled into symmetric cells and their impedance was measured at different temperatures to investigate the effect of the collector surface chemistry on the activation energy (Fig. 3g, Supplementary Fig. 18, Supplementary Table 6). The activation energies (Ea) in AlGa/Na and Al/Na were calculated by fitting the slope to the Arrhenius equation and were 19.53 kJ mol^−1^ and 27.18 kJ mol^−1^, respectively. The enrichment of Ga at AlGa grain boundaries serves as an effective nucleation site, reducing the initial nucleation size of Na. This not only stabilizes the direct electrical contact between Na and AlGa but also promotes a more homogeneous deposition interface, leading to uniform SEI layer formation. Consequently, the AlGa/Na interface exhibits lower energy barriers for both charge transfer and ion diffusion through the SEI layer. The exchange current densities were tested by Tafel curves to probe the kinetic intrinsic rates of Na^+^ transfer at the electrode/electrolyte interface. As shown in Fig. 3h, the exchange current densities of AlGa and Al are 2.04 and 0.60 mA cm−2, respectively, which indicates improved Na^+^ transport kinetics at the AlGa interface.

To further investigate the reason for the uniform deposition of Na on AlGa, contact angle tests were shown in Fig. 3i, the contact angles of AlGa and Al collector are 20.6° and 42.2°, respectively, indicating good electrolyte wettability, thereby promoting uniform Na deposition. Uniform and dense Na deposition is a key factor in achieving highly reversible Na. The nucleation processes and growth morphologies of Na at a current density of 1 mA cm^−2^ were evaluated by SEM. The deposited Na exhibits a more intimate interface with AlGa. In contrast, Na grows sparsely on pristine Al, with a clearly defined Na and Al boundary at a low areal capacity of 0.1 mAh cm^−2^ (Supplementary Fig. 19), which indicates inhomogeneous Na deposition and poor interfacial adhesion on the Al substrate. High-magnification SEM images further reveal that the AlGa substrate maintains a dense and uniform Na deposition morphology even at high magnification, whereas the pristine Al substrate shows pronounced height variations and localized pit-like features. This behavior can be attributed to Ga enrichment at the AlGa grain boundaries, which provides effective nucleation sites and reduces the initial nucleation size of Na. As a result, stable electrical contact between Na and AlGa is maintained, and a more uniform Na deposition interface is achieved. Further deposition of Na (1 mAh cm^−2^) was uniformly and densely distributed on the AlGa collector, while many cracks are shown on the Na layer on Al (Fig. 3j). The thickness of Na on AlGa is only 8.4 μm, which is close to the ideal deposition (8 μm) (Supplementary Fig. 20). In contrast, Na deposition on bare Al tends to preferentially grow on pre-deposited Na nuclei, leading to a nonuniform Na deposition process. As a result, Na accumulates excessively in certain regions (up to ~31 μm) while other areas remain thin (1 μm) or nearly bare. These observations demonstrate that the tip effect induces inhomogeneous Na deposition. After 15 cycles, Na remained nearly perfectly deposited on AlGa (Supplementary Fig. 21). For stripping (Fig. 3k), there is basically no residual dead Na on the AlGa, whereas isolated dead Na are observed on the Al foil, which cannot be effectively stripped back to the positive electrode due to the loss of electrical contact, leading to the rapid degradation of the battery capacity.

Furthermore, the evolution of the AlGa current collector and the Na deposition behavior after prolonged cycling were investigated. The microscale migration of Ga has been carefully considered. Vertical migration was investigated through EDS line scans and TOF-SIMS depth profiling, which were performed on AlGa after 50 and 100 cycles (with Na stripped), along with a control sample stored without cycling. Cross-sectional SEM shows that Ga remains enriched near the surface after cycling, gradually decreasing toward the bulk (Supplementary Fig. 22), although some fluctuations may arise from the limited resolution of EDS. To obtain more reliable depth information, TOF-SIMS analysis was conducted (Supplementary Figs. 23–25). The Ga-enriched region increases from ~9.3 nm to ~28.8 nm after 50 cycles, indicating nanoscale migration, despite some uncertainty from the SEI layer. Both Ga^+^ and NaGa^+^ signals decrease significantly after ~250 s sputtering, with Ga becoming nearly constant beyond ~500 s (~57.5 nm). Notably, NaGa^+^ is enriched in the near-surface region ( ~ 4.6–18 nm), while Ga^+^ is strongest within the topmost ~4.6 nm, suggesting reversible alloying at the surface and the formation of a subsurface Na–Ga layer that suppresses further inward migration. After 100 cycles, the positions of the Ga^+^ and NaGa^+^ enriched regions remain nearly unchanged, indicating stable Ga distribution during cycling. The Na deposition morphology after 50, 100, and 850 cycles by plating 1 mAh cm^−2^ Na onto the AlGa surface was tested (Supplementary Fig.26). The deposited Na remains smooth and uniform even after prolonged cycling. AlGa was simultaneously detected in the Na stripping state after 100 and 860 cycles (Supplementary Figs. 27–28). The slight surface exfoliation is observed on the AlGa current collector due to Al dissolution, while the overall surface remains essentially flat. EDS mapping confirms uniform elemental distribution and negligible Na accumulation, indicating suppression of “dead Na” and overall structural stability during long term cycling.

TOF-SIMS (Supplementary Figs. 29–30) was employed to characterize the composition and spatial distribution of the solid electrolyte interphase (SEI) formed on the AlGa and Al surface after cycling. Typical organic fragments, including NaCHO^−^, CH_3_ONa^−^, and C_2_HO^−^, are commonly attributed to the decomposition of diglyme. In the AlGa sample, the intensities of these organic fragments decrease gradually and uniformly from the surface toward the interior, whereas in the Al sample they exhibit a much higher surface concentration followed by a sharp decline, indicating more severe ether decomposition reactions on the Al surface. Additionally, inorganic fragments such as Na_2_O^−^, NaF_2_^−^, and CF^−^, which mainly originate from NaPF_6_ decomposition, display a more homogeneous depth distribution in the AlGa sample, while a highly nonuniform distribution is observed for the Al sample. Moreover, a higher abundance of inorganic species, including NaCO_2_^−^ and NaGa^+^, is detected in the AlGa sample, which is expected to help the SEI with enhanced mechanical robustness. Therefore, the modified AlGa exhibits a SEI with uniformly distributed organic and inorganic components in both the lateral and depth directions, whereas the SEI formed on the Al electrode is highly heterogeneous.

### Effect of Al^3+^ on NVP positive electrode

The effect of dissolved Al^3+^ on the positive electrode was also evaluated. Crystallographic studies have shown that the NVP consists of a polyanionic [V_2_(PO_4_)_3_]_3_ framework accommodating three Na^+^, corresponding to V in the +3 oxidation state^47^. When two Na ions are removed (at ~3.4 V (vs. Na^+^/Na)), the redox couple of V^3+^/^4+^ enables a specific capacity of 117.6 mAh g^−1^. However, due to the irreversible Na⁺ loss, a portion of vanadium is oxidized to the +4 state, which leads to structural degradation of the positive electrode. Therefore, beyond enhancing Na reversibility, the dissolved Al^3+^ plays dual functions: (1) enabling electrochemical insertion of electrolyte-derived Na⁺ into NVP, while (2) participating in CEI formation. This synergistic mechanism effectively stabilizes the NVP crystal structure during cycling.

**Fig. 4:**
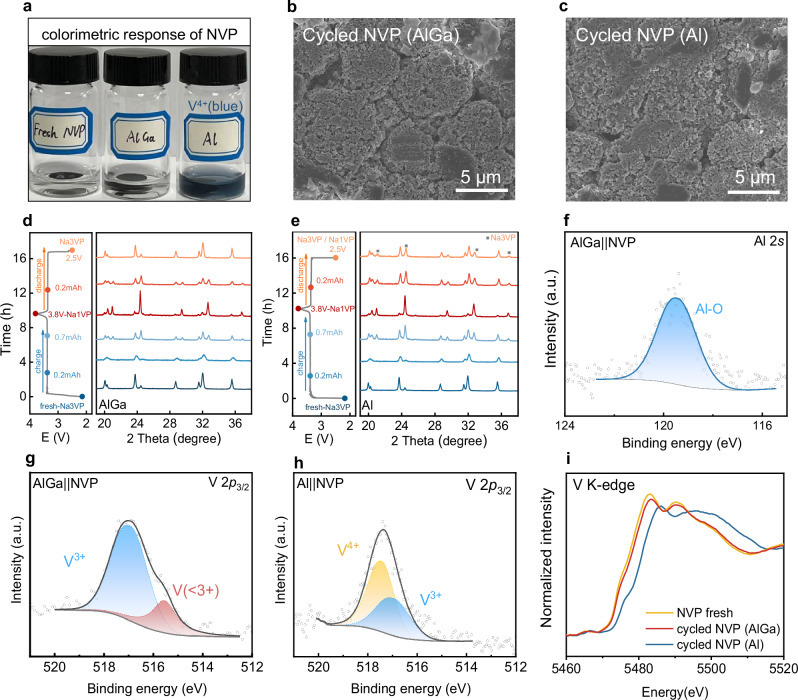
Effect of Al^3+^ on NVP positive electrode. **a** Optical images of fresh NVP and NVP after 15 cycles (1C) with AlGa and Al, V^4+^ displays blue in H_2_O. SEM images of the NVP surface after 50 cycles with AlGa (**b**) and Al (**c**). ex situ XRD patterns of NVP cycled with AlGa (**d**) and Al (**e**) for the sodiation and desodiation processes, corresponding to the phase transition between Na_3_V_2_(PO_4_)_3_ (Na3VP) and NaV_2_ (PO_4_)_3_ (Na1VP). **f** XPS spectra of Al 2*s* for the surface of NVP cycled with AlGa. XPS spectra of V 2*p*_3/2_ for the surface of NVP cycled with AlGa (**g**) and Al (**h**). **i** XANES spectra of V K-edge for fresh NVP and NVP after 15 cycles.

To visually and quickly observe the Na deficiency of the NVP positive electrode, the NVP cycled in full cell for 15 cycles was removed and immersed in ultrapure water for 0.5 h, based on the unique color characteristics of V^4+^ in H_2_O (Fig. 4a). For NVP cycled with Al, the limited amount of Na^+^ inserted into the NVP may lead to dissolution of V^4+^ ions from the framework, and thus even a brief soaking led to a visible color change^48^. Conversely, the fresh NVP and the NVP circulated with AlGa maintained the structural stability with negligible solubility in water and did not exhibit any color change. As shown in Supplementary Fig. 31, after 15 cycles, the NVP (cycled with AlGa) electrode thickness approaches that of fresh NVP (103 μm vs 106 μm). However, the thickness of the NVP cycled with Al decreased significantly (86.8 μm), indicating substantial collapse of the NVP structure. High-magnification SEM images reveal that the surface particles of the NVP sample paired with AlGa exhibit intact boundaries. In contrast, the surface morphology of NVP cycled with Al appears blurred boundaries, suggesting the presence of a thicker CEI layer. In Supplementary Fig. 32, even at high positive electrode loading (47.40 mg cm^−2^), the thickness and particle integrity of NVP-based positive electrodes are well maintained after cycling. A more detailed SEM observation of the NVP positive electrode show that the polycrystalline NVP particles that had been cycled with AlGa for 50 cycles basically maintained the original large spherical structure, while the NVP cycled with Al showed a large amount of fragmentation (Fig. 4b, c).

As a powerful measurement for detecting structural phase transitions in materials, XRD measurements were performed on NVP positive electrode at different charging/discharging stages of the first cycle. It can be found that the Na^+^ continuously deintercalates during the first charging stage, and the main phase becomes Na1VP when charging to 3.8 V^49,50^ (Fig. 4d, e). During discharge, Na^+^ continuously enters, and some of the phases are transformed from Na1VP into Na3VP. In the case of Al foils, due to the highly irreversible behavior of Na^+^, a substantial amount of the Na1VP phase remains after discharge to 2.5 V, ultimately forming a mixture of Na1VP and Na3VP phases. For the NVP positive electrode cycled with AlGa, due to the solvation compensation of Al^3+^ and the high reversibility of Na^+^, it essentially returns to the Na3VP phase.

To further verify the surface condition of the NVP positive electrode, XPS measurements were performed on the NVP after 15 cycles. Fig. 4f shows the surface of the NVP cycled with AlGa, Al–O is detected in the Al 2*s* spectrum, demonstrating that dissolved Al^3+^ is also involved in the formation of CEI, which has been reported to be conducive to the inhibition of positive electrode surface morphology evolution and phase transformation^51–53^. TOF-SIMS depth profiling of the NVP surface also revealed abundant Al related fragments: Al^+^, AlO^−^, AlO_2_^−^, and AlOC^−^ ion fragments (Supplementary Fig. 33). No signal of Al was observed on the surface of NVP cycled with Al foil (Supplementary Fig. 34). In V 2*p*_3/2_ spectra, only one V (+3) peak at 517.0 eV signal was observed in the fresh NVP^54^ (Supplementary Fig. 35). For the NVP cycled with AlGa (Fig. 4g), peaks at 517.0 eV (V (+3)) and 515.5 eV were found, which may be associated with the intercalation of Al^3+^, resulting in a further reduction of the V valence state^55^. In contrast, a large amount of vanadium (+4) was found with the NVP cycled with Al (Fig. 4h), again demonstrating the oxidation of V^3+^ due to Na^+^ deficiency. These are further supported by the X-ray absorption near-edge structure (XANES), which shows that after the same number of cycles, the characteristic rising edge displacement of the NVP cycled with AlGa is close to that of fresh NVP, with a fuller reduction reaction (Fig. 4i)^56^.

### Electrochemical performance of AlGa

**Fig. 5:**
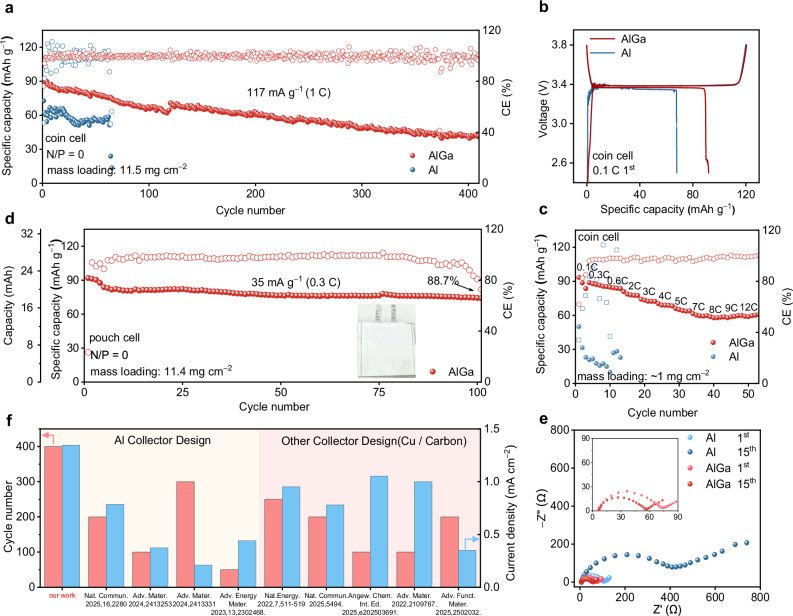
Characterization of Electrochemical performance of full cells. **a** The cyclic stability of AlGa||NVP and Al||NVP cells at 1C, 1C = 117 mA g^−1^. **b **The voltage-capacity curves of the AlGa||NVP and Al||NVP at first cycle. **c** Rate performance of AlGa||NVP and Al||NVP cells. **d** The cyclic stability of AlGa||NVP pouch cells. **e **The EIS of AlGa||NVP and Al||NVP in a fully discharged state after different cycles. **f** Comparison of the cycling performance of full cells between recently reported works and this work. The source of the literature data shown in this figure can be found in Supplementary Information, Table 7.

AFSMBs were assembled using the NVP positive electrode to explore the potential of AlGa for practical applications. It should be noted that none of the assembled AFSMBs was subjected to any pre-sodiation treatment that could affect the capacity. Fig. 5a and Supplementary Fig. 36 show the cycling stability of AlGa||NVP and Al||NVP full cells at 117 mA g^−1^ (1C). The AlGa||NVP (11.4 mg cm^−2^) full cell achieved more than 50% capacity retention after 400 cycles, while the capacity of NVP cycled with Al rapidly decayed after only 60 cycles. The discharge profile shows an initial discharge capacity of 92 mAh g^−1^, with a sloping feature at 2.6 V, corresponding to Al^3+^ dissolution. The initial discharge capacity of the Al sample is only 67.9 mAh g^−1^ (Fig. 5b). The AlGa||NVP cell exhibits excellent rate performance. As the rate increases from 0.1 C to 12C, the AlGa||NVP cell delivers a discharge capacity of 60.8 mAh g^−1^ at 12C (Fig. 5c). The slight capacity increase beyond 8C is attributed to accelerated Al^3+^ dissolution at high current densities, which induces additional charge compensation and enhances Na^+^ utilization despite kinetically limited Na^+^ transport. After cycling, PP separators containing absorbed electrolyte were immersed in Al reagent solution (Supplementary Fig. 37), and UV–Vis spectroscopy showed increased Al³⁺ dissolution at higher current densities (12C > 8 C> 4 C). At a high loading (44 mg cm^−2^), the AlGa||NVP cell stably cycled for more than 50 cycles at 0.2C (Supplementary Fig. 38). To further explore the practical applicability, a pouch cell (NVP: 11.4 mg cm^−2^) was assembled. In Fig. 5d, the initial discharge capacity of the pouch cell AlGa||NVP is 91.9 mAh g^−1^ at 11.7 mA g^−1^ (0.1C), and it maintains stable cycling for over 100 cycles and retains 88.7% capacity retention at 35 mA g^−1^ (0.3C). Fig. 5e shows that the EIS results for Al||NVP exhibit a sudden increase in charge transfer resistance and ion transport resistance from 100 Ω to 400 Ω after 15 cycles, implying an unstable thick SEI as well as poor electron transport paths due to repeated inhomogeneous Na deposition and the presence of dead Na. In contrast, AlGa exhibits relatively stable resistance (58 Ω, 75 Ω). The current collector design exhibits competitive performance compared with reported systems^2,20,37–41,57,58^. (Fig. 5f and Supplementary Table 7).

## Discussion

In conclusion, for AFSMBs, the intrinsic Na deficiency cannot be fundamentally resolved merely by enhancing interfacial compatibility, as the system inherently lacks a compensable Na reservoir. Therefore, in addition to enhance the sodophilicity of the Al substrate, controlled dissolution of Al^3+^ drives the self-migration of Na^+^ from electrolyte to the positive electrode, thereby achieving Na^+^ compensation. AlGa enriches Ga at grain boundaries, leading to a microalloying reaction that promotes high-density Na nucleation and improves Na reversibility. The asymmetric cell exhibits high reversibility and achieves stable plating/stripping over 2500 cycles at 1 mA cm^−2^, 1 mAh cm^−2^. More fundamentally, Ga-induced grain-boundary liquefaction facilitates Al^3+^ dissolution, implying that the sufficiently amount of Na^+^ intercalation to the NVP. The dissolved Al^3+^ participates in the composition of the CEI, helping stabilize the structure of the NVP and preventing the phase transition. The AlGa||NVP (11.4 mg cm^−2^) coin cell achieved more than 50% capacity retention at more than 400 cycles. The initially anode-free pouch cells matching with NVP (11.4 mg cm^−2^), exhibit cycling stability for more than 100 times with a capacity retention rate of 88.7%. A single-layer pouch cell of 201.50 Wh kg^−1^ with positive electrode mass loading of 47.40 mg cm^−2^ was successfully operated. This ion-compensated scheme provides a promising idea for the design of collectors for AFSMBs.

## Methods

### Synthesis of AlGa

All of the following operations are performed in the glovebox filled with argon gas (O_2_ < 0.01 ppm, H_2_O < 0.01 ppm). AlGa: Gallium (Ga, Aladdin) was evenly brushed onto the Al foil (Macklin, 99.9%, 100 μm), and then scrape the excess Ga off the surface. It was left to stand (30 °C) for one day and then used. H-AlGa: The prepared AlGa was put into a muffle furnace and heated to 600 °C for 5 h and then cooled with the furnace. S-AlGa: The aluminum foil was heated to 600 °C for 5 h, then cooled in the furnace. Then brush Ga evenly on the treated aluminum foil, and leave it to stand for one day before use. The Al foil was used as received without any further treatment.

### Synthesis of NVP positive electrodes

Positive electrode wet process: NVP powder (92.8 wt%, particle size ~20 μm, Shenzhen Kejing Star Technology Co., Ltd.), super P (Guangdong Canrd New Energy Technology Co., Ltd., 2 wt%), carbon nano-tube (CNT, 2 wt%), and poly(vinylidene fluoride) (PVDF, 4.2 wt%) were mixed and hand-milled for 30 min in planetary mixer, and then dissolved in n-methyl-2-pyridinone (NMP, Sigma-Aldrich, ≥99 %) solvent and stirred using a planetary mixer until a homogeneous slurry was formed with an loading of 1 and 11.4–11.5 mg cm^−2^. The slurry was prepared under ambient atmosphere at room temperature. The slurry was cast onto carbon-coated Al foil (99.35% purity, 15 μm thickness) using a doctor blade, followed by vacuum drying at 80 °C for 12 h. Semi-dry process: NVP powder (92 wt%, NVP- polycrystalline (particle size 20 μm): NVP-single crystal (1–2 μm) = 4: 1), CNT (1 wt%), graphene (2 wt%) were mixed and hand-milled for 30 min in marmalade mortar. Then slowly add the PTFE mixture (PTFE, 5 wt%, first dispersed PTFE in water and ethanol), grinding as it is added, until all the powders are gathered into soft flakes. Rolled to the proper thickness using a roller machine. The conductive adhesive (Shenzhen Kejing Star Technology Co., Ltd., 50 wt%) and Super P (1 wt%) using a planetary mixer, and the mixed adhesive was applied to the carbon-coated aluminum foil with a mass of about (1.8 mg cm^−2^). Immediately, the prepared dry sheet was pressed onto the conductive adhesive. Then dried using vacuum oven. The positive electrode mass loading was 47.40 mg cm^−2^.

### Characterizations

#### Materials characterization

All sample preparation and handling were conducted in an argon-filled glovebox (H2O < 0.1ppm, O2 < 0.1ppm) unless otherwise stated. After cycling, electrodes were rinsed with diglyme unless otherwise stated, vacuum-dried at room temperature, and transferred in sealed holders to prevent contamination.

The morphology and elemental distribution of the samples were characterized by scanning electron microscopy (SEM, Regulus SU8010) and energy-dispersive X-ray spectroscopy (EDS). The XRD patterns were performed by X-ray powder diffraction (XRD, D-MAX 2200 VPC) with Cu Kα radiation. The ex-situ XRD (Rigaku, Smartlab SE) patterns were collected for the sodiation and desodiation processes. The complexation of aurintricarboxylic acid triammonium salt and Al^3+^ was monitored using ultraviolet-visible absorption spectroscopy (UV-3600) at different numbers of cycles. The surface chemical state of Al and AlGa was analyzed using X-ray photoelectron spectroscopy (XPS, ESCALAB Xi+), and depth profiles were obtained using Ar^+^ sputtering (0, 120, 300 s) at 2000 eV and 2 × 2 mm area, as well as analyzing the surface chemical state of NVP after cycling. The electrolyte wetting of the collector surface was tested using a contact angle tester OCA20 from Data Physics, Germany. The valence state of vanadium(V) in the NVP positive electrode was characterized using a tabletop X-ray near-edge absorption technique (XANES, Rapid XAFS). TOF-SIMS (IONTOF M6) was employed to characterize the three-dimensional structure of AlGa and SEI/CEI, using a 30 kV Bi^+^ primary ion beam for AlGa and a 30 kV Bi3^+^ beam for SEI/CEI analysis, combined with 500 eV Ar^+^ sputtering. The analysis area was 100 μm × 100 μm, while the sputtering area was 300 μm × 300 μm. Detailed experimental parameters for each measurement are provided in the corresponding Supplementary Figs. Nanoindentation tests were carried out using an Agilent G200X equipped with a Berkovich indenter. A maximum load of 2 mN was applied with a dwell time of 2 s at the peak load. The resistance of the AlGa and Al was measured using a four-point probe system (HPS2661, Haierpa Electronics, Changzhou, China). Measurements were conducted on flat samples at room temperature. Each measurement was repeated at three different locations, and the average value is reported. ICP-OES measurements were performed using an Agilent ICP-OES 5110 system. The RF power was set to 1.20 kW, with a plasma gas flow of 12.0 L min^−1^, auxiliary gas flow of 1.00 L min^−1^, and nebulizer gas flow of 0.70 L min^−1^. The sample uptake delay and instrument stabilization delay were set to 20 s and 15 s, respectively. The replicate read time was 5 s, and three replicates were recorded for each sample. Crystal structure visualization of Na_3_V_2_(PO_4_)_3_ was generated using VESTA based on the reported crystallographic structure of Na_3_V_2_(PO_4_)_3_^49,59^.

#### Electrochemical measurements

Coin cells (CR2032), Swagelok cell and pouch cells were assembled in a glovebox (MIKROUNA, SUPER) filled with argon (H_2_O and O_2_ < 0.1 ppm). The auxiliary voltage experiment was conducted in a three-electrode cell device (Swagelok). Current was applied between the NVP and AlGa electrodes, while monitoring the voltage between NVP and AlGa and the voltage between AlGa and Na. The charge and discharge cutoff conditions were when the AlGa | |NVP cell voltage reached 3.8 V and 2.2 V, respectively. Celgard 2500 membranes were used as separators. The electrolyte (1 M NaPF_6_ in diglyme, Suzhou Duoduo Chemical Co., Ltd.) was stored and handled in an argon-filled glovebox, and was transferred using polypropylene (PP) pipette tips.CR2032-type coin cells were assembled using an AlGa electrode (12 mm in diameter) as the negative electrode and an NVP positive electrode (10 mm in diameter). For half-cells, Cyclic voltammetry (CV, DongHua Testing Technology Co., Ltd.) was scanned at 0.1 mV s^−1^. Tafel (IVIUM Vertex. One.) and Electrochemical Impedance Spectroscopy (EIS, DongHua Testing Technology Co., Ltd.) were tested on Al or AlGa deposited at 1 mA cm^−2^, 1 mAh cm^−2^ of Na. Electrochemical impedance spectroscopy (EIS) measurements were conducted in the frequency range of 0.1 Hz to 10 kHz with an amplitude of 10 mV under potentiostatic conditions. Prior to the measurement, the cell was held at open-circuit potential for 10 s. And then the data were fitted according to the Arrhenius equation (G = A exp(−*E*_a_/RT)) to calculate the apparent activation energy *E*_a_, where *A* is the prefactor, *R* is the universal gas constant, and *T* is the temperature (303–323 K). The Coulombic Efficiency (CE) values of the long cycle curves of the asymmetric cells and the long performance of the full cells were measured at 30 °C using a LAND electrochemical test system (Wuhan LAND Electronics Co., Ltd.) under constant current conditions. The CE was calculated as the ratio of the stripping capacity to the plating capacity of the asymmetric cells. The mathematical methods applied to calculate the specific energy values: specific energy = (positive electrode areal capacity **× **average discharge voltage)/total assigned density.

## Supplementary information

Supplementary Information

Transparent Peer Review file

## Source data

Source Data

## Data Availability

The data generated in this study are provided in the Supplementary Information/Source Data file. Source data are provided with this paper.
