# Supplementary material for: Grain boundary engineered aluminum current collector for energy-dense initially anode-free sodium metal batteries
Source: Nat Commun. 2026 Jun 6;17:7225. doi: 10.1038/s41467-026-74177-8 (PMC13396730; doi:10.1038/s41467-026-74177-8)
Supplement: Supplementary file 1 — Supplementary Information [file 41467_2026_74177_MOESM1_ESM.pdf]

Supporting Information

Grain boundary engineered aluminum current collector for energy-dense initially anode-free sodium metal batteries

Xueying Zheng[1], *Dongpeng Yu*[1], *Fei Tian*[1], *Danni Lei**[1], *Chengxin Wang**[1]

Affiliations

1.State Key Laboratory of Optoelectronic Materials and Technologies, School of Materials Science and Engineering, Sun Yat-sen (Zhongshan) University, Guangzhou 510275, China.

***Corresponding authors**

E-mail addresses: leidanni@mail.sysu.edu.cn (D. Lei), Wchengx@mail.sysu.edu.cn (C. Wang)

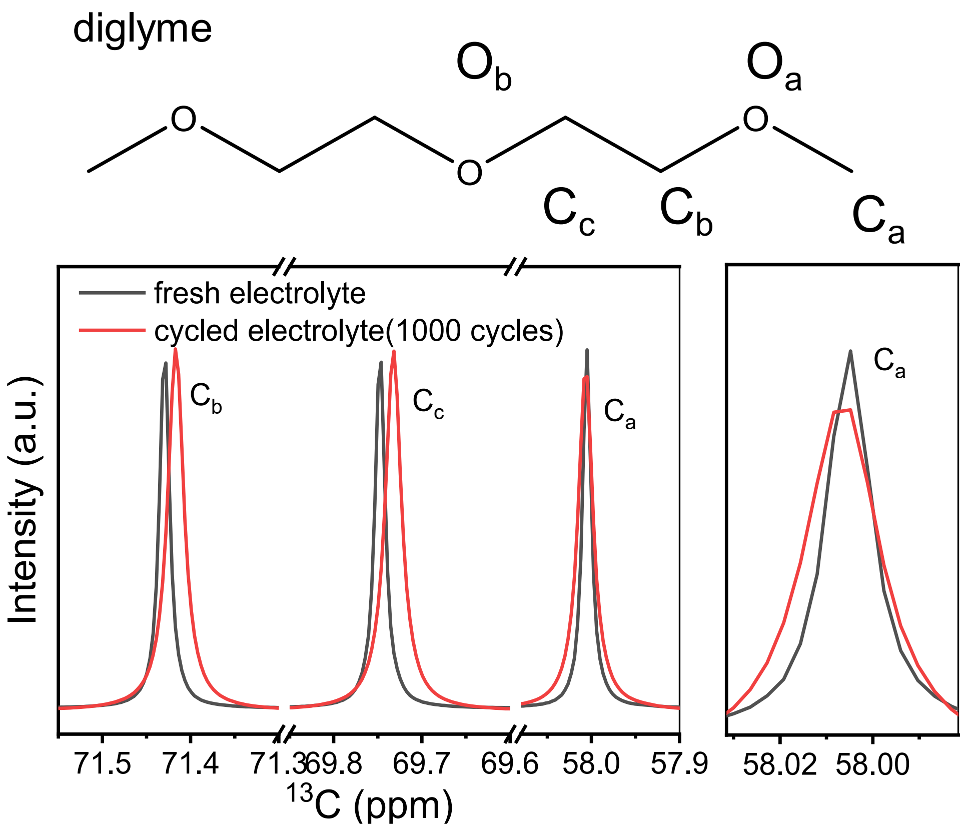

Supplementary Fig. 1 ^{13}C NMR of fresh electrolyte and cycled electrolyte (1000 cycles). The right panel shows a magnified view of the Ca peak. The x-axis is broken for clarity.

The cycled electrolyte (~30 μL) was diluted with 1 mL of pristine electrolyte (1 M $NaPF_6$ in diglyme) to obtain a sufficient volume for analysis. Although this dilution inevitably reduces the concentration of dissolved Al^{3+} and may partially weaken their influence on the solvent environment, the observed spectral shifts still clearly indicate the interaction between Al^{3+} and the ether oxygen atoms of diglyme.

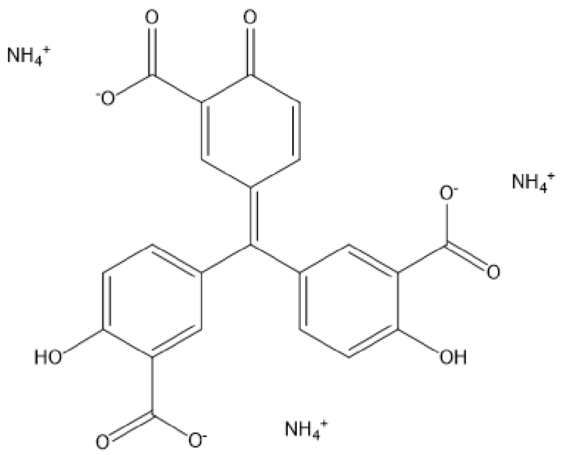

Supplementary Fig. 2 Chemical structure of aurintricarboxylic acid triammonium salt (Al reagent).

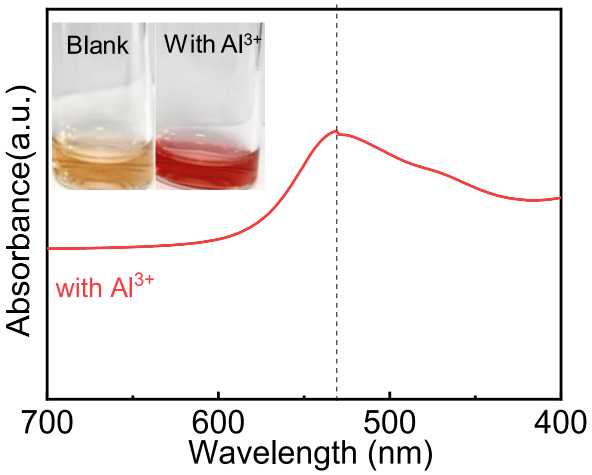

Supplementary Fig. 3 UV-Vis spectrum of the reaction between Al reagent and Al^{3+}. The insets are optical photographs before and after the reaction.

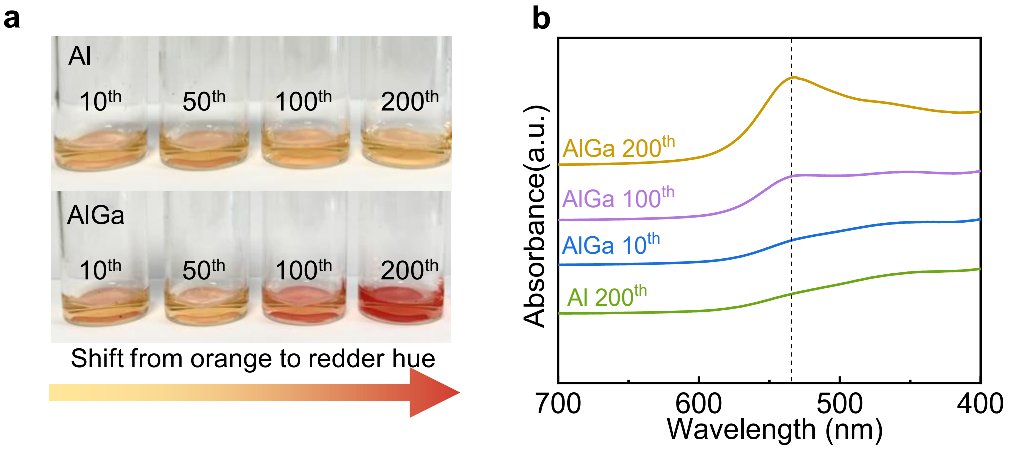

Supplementary Fig. 4 (a) Optical images of electrolytes infiltrated on PP cycled in Na||Al and Na||AlGa with different number of cycles with Al reagent. (b) UV-Vis spectra of electrolyte-infused PP separators (Na||Al and Na||AlGa, different cycles) after reaction with Al reagent.

a

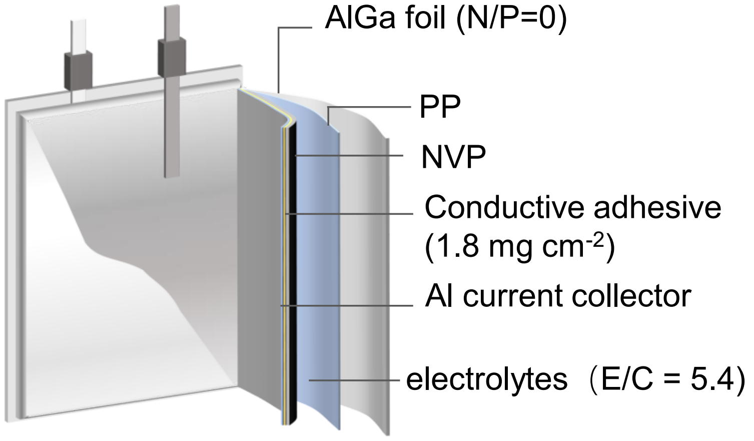

b

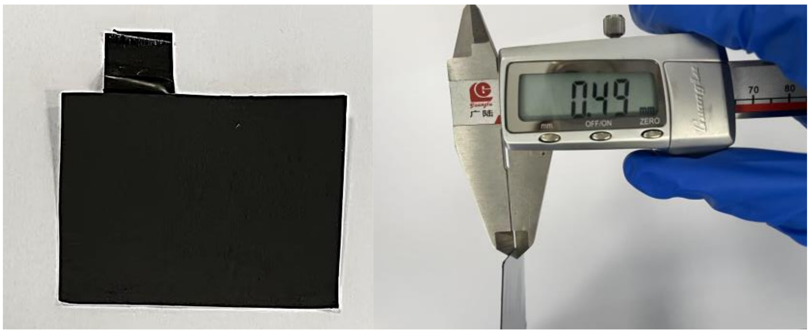

Supplementary Fig. 5 (a) Schematic illustration of the single-layer pouch cell configuration. (b)Optical photo of the NVP positive electrode (5 cm × 4 cm) and its thickness (0.49 μm).

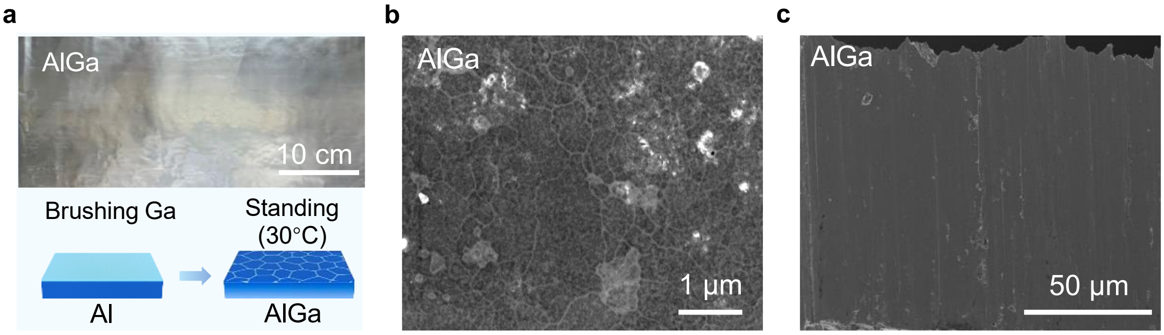

Supplementary Fig. 6 (a) The optical photo of AlGa. The schematic diagram of AlGa preparation. SEM image of AlGa surface (b) and cross-sectional morphology (c).

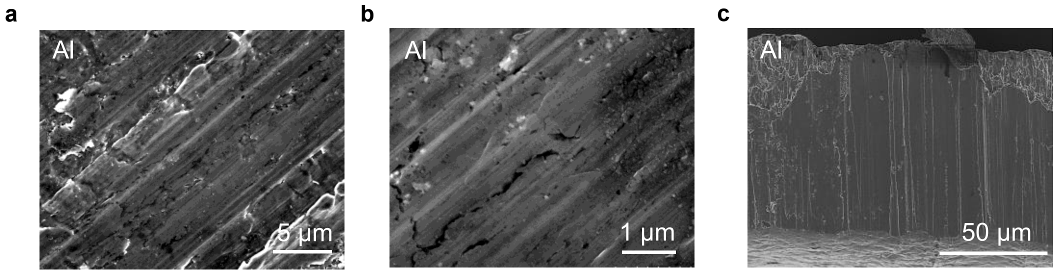

Supplementary Fig. 7 SEM image of Al surface (a-b) and cross-sectional morphology (c).

a

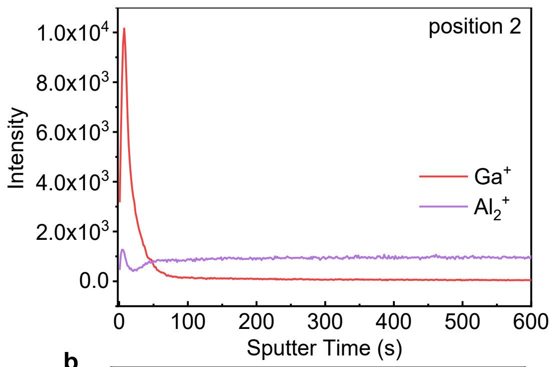

b

Instrument: IONTOF M6-Hybrid	
Acquisition phase	
Ion species	Bi1 (positive mode)
Energy	30 keV
Ion current	1.014 pA
Raster size	100 μm x 100 μm
pixel dimensions	128 x 128
Mass range	0 - 270 u
Mode	All-Purpose (Hybrid)
Analysis time	600 s (spectra)
Sputter beam	
Ion species	Ar
Energy/Current	500 eV / 62.920 nA
Raster size	300 μm x 300 μm
Sputtering time	600 sec

c

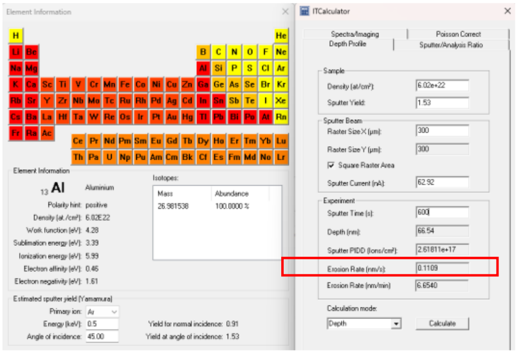

Supplementary Fig. 8 (a) The variation of fragment species intensity with sputtering time. (b) Actual conditions of TOF-SIMS testing. (c) Calculation of the Al etching rate corresponding to the set conditions within the instrument. For metal Al, the etching rate is 0.1109 nm s^{-1}. At an etching time of approximately 100 s, there is no significant change in Ga$^+$ ions. Therefore, the thickness of the Ga layer is estimated to be approximately 11.1 nm.

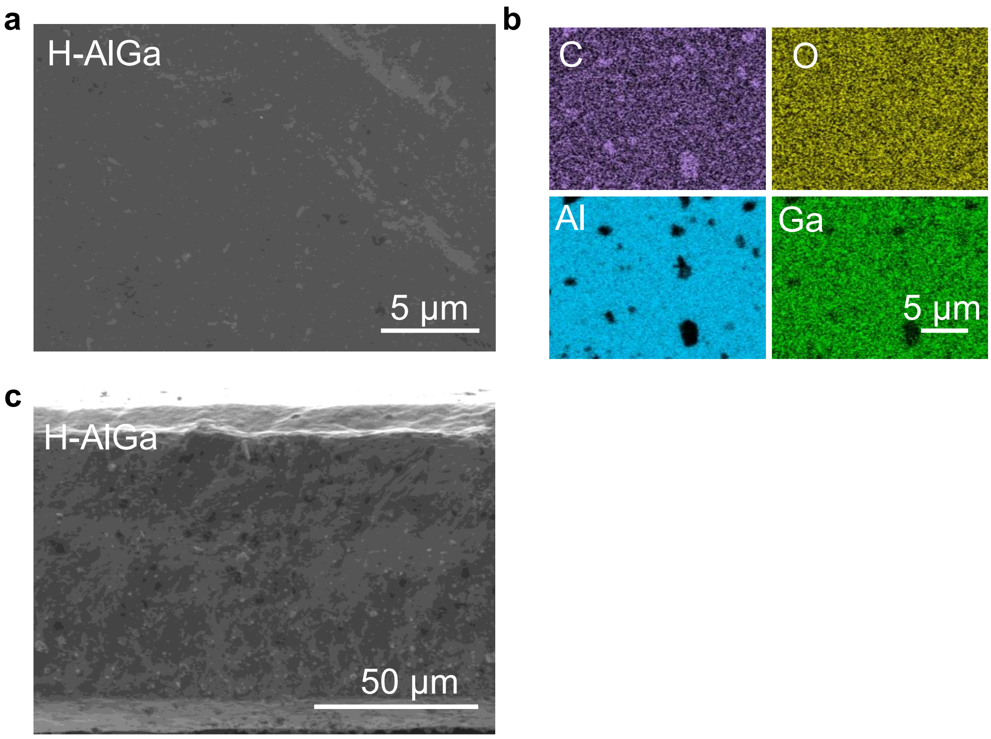

Supplementary Fig. 9 SEM image of H-AlGa surface (a), EDS mappings (b) and cross-sectional morphology (c). The EDS mapping analysis revealed an atomic ratio of Al to Ga as 99.86 : 0.14 at the surface region.

Due to the annealing treatment, Ga has been uniformly incorporated into the Al lattice, and there is no excess Ga precipitated in the grain boundaries.

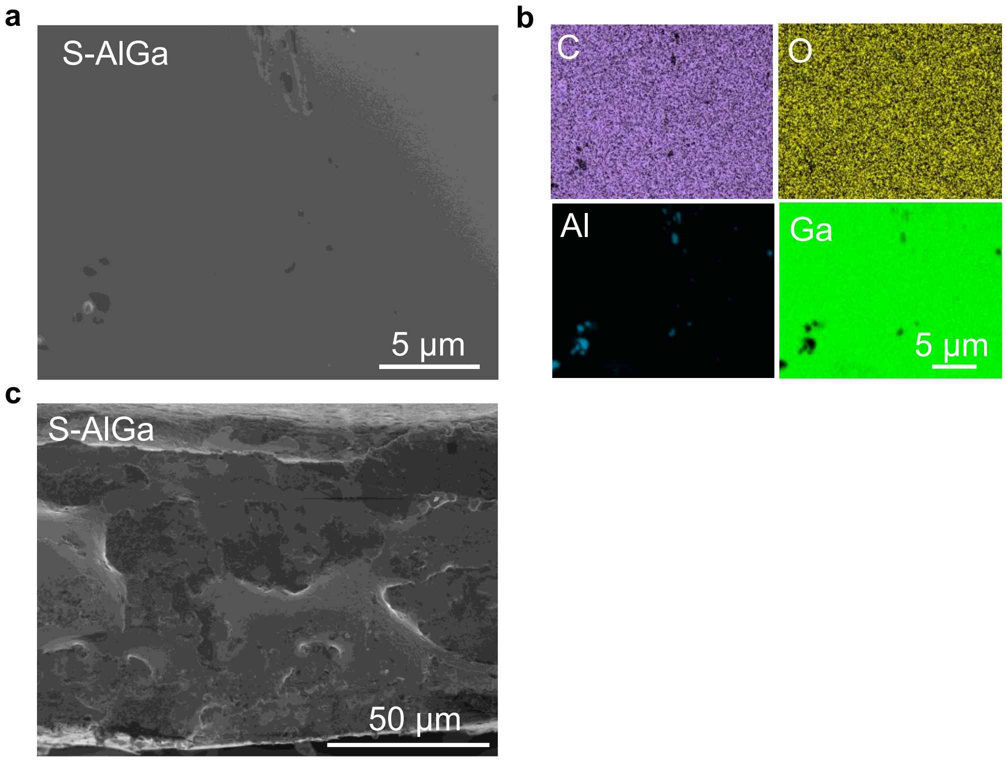

Supplementary Fig. 10 SEM image of S-AlGa surface (a), EDS mappings (b) and cross-sectional morphology (c). The EDS mapping analysis revealed an atomic ratio of Al to Ga as 6.50 : 93.50 at the surface region.

Due to the annealing treatment before brushing Ga, the Al orientation becomes more homogeneous, the grains become larger, and the grain boundaries become significantly smaller, so that most of the Ga covers the surface of Al at room temperature.

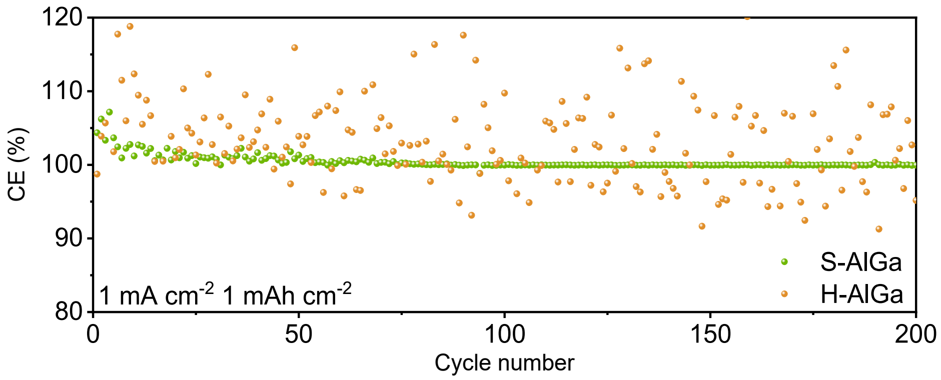

Supplementary Fig. 11 Coulombic efficiency of Na plating / stripping on the S-AlGa and H-AlGa at 1 mAh cm^{-2} and 1 mA cm^{-2}. (Cycle cut-off condition: 200 cycles)

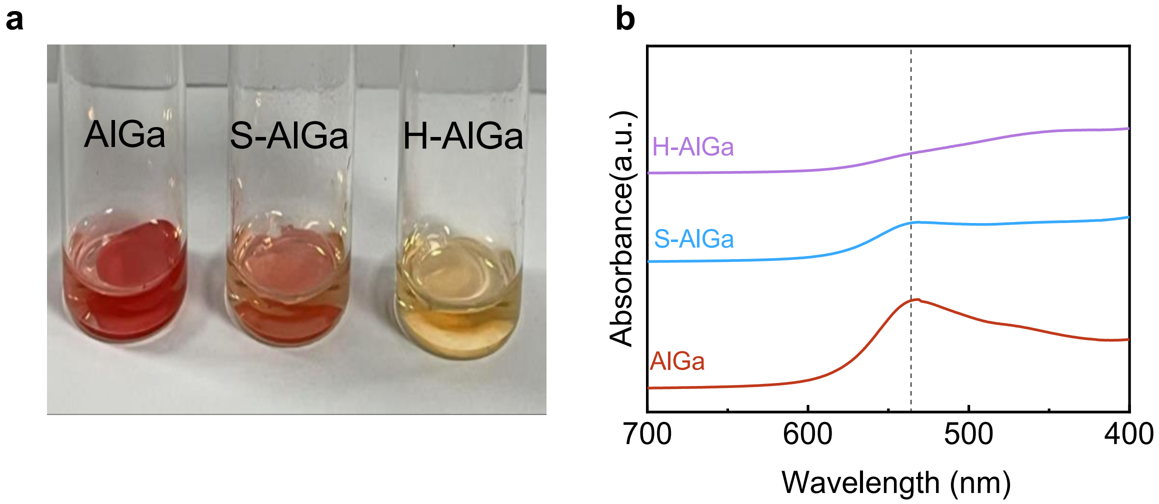

Supplementary Fig. 12 (a)Optical images of electrolytes infiltrated on PP cycled from Na||AlGa, Na||S-AlGa and Na||H-AlGa for 200 cycles reacted with Al reagent solution. (b) Corresponding ultraviolet and visible spectrum absorption spectrum.

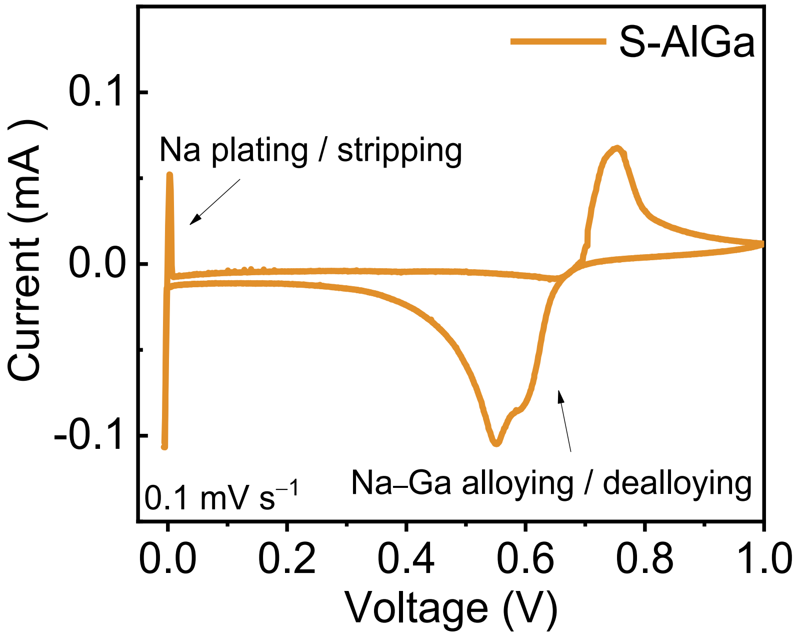

Supplementary Fig. 13 CV curves of Na||S-AlGa cycle at 0.1 mV s^{-1}. The voltage scanning range is -0.005V–1 V.

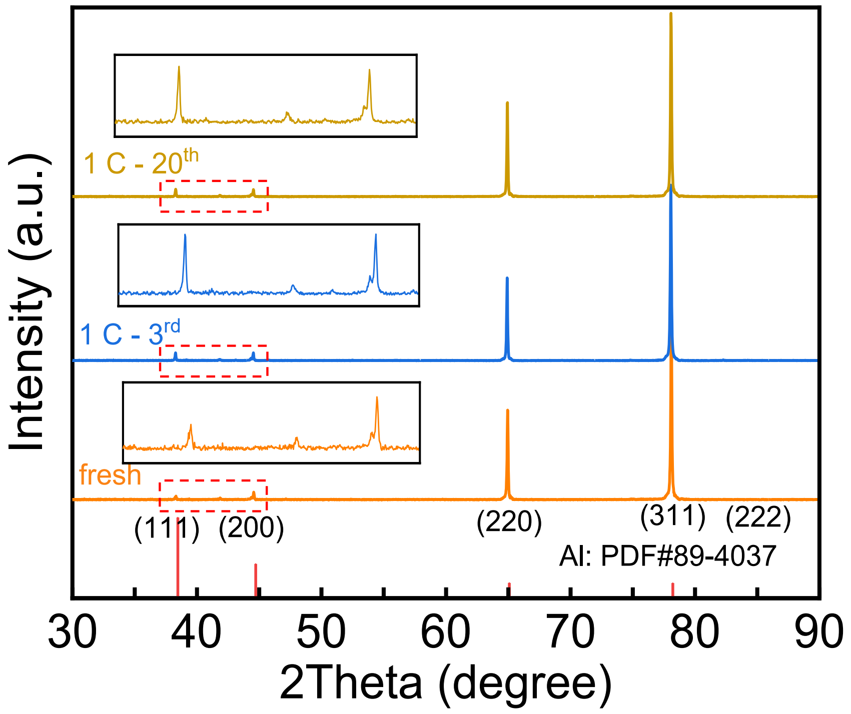

Supplementary Fig. 14 XRD patterns of cycled AlGa. The enlarged view of the (111) and (200) crystal plane within the box in the figure. It can be observed that the intensity ratio of each crystal plane changes during the cycle.

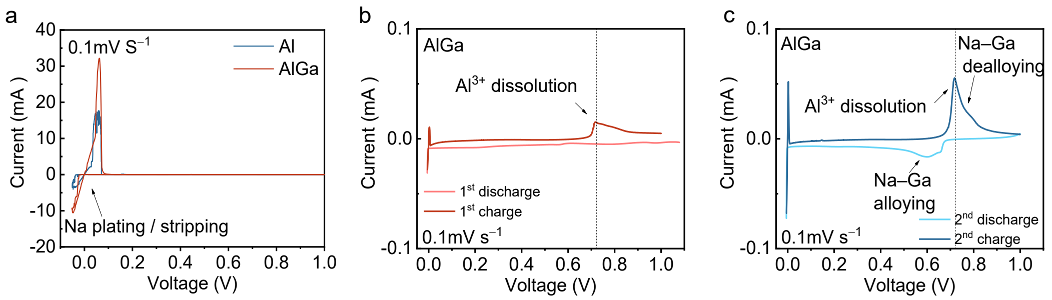

Supplementary Fig. 15 CV curves Na||AlGa and Na||Al cycling at 0.1 mV s^{-1}. The voltage scanning range is -0.05V–1V (a) and -0.005V–1 V (b-c).

a b

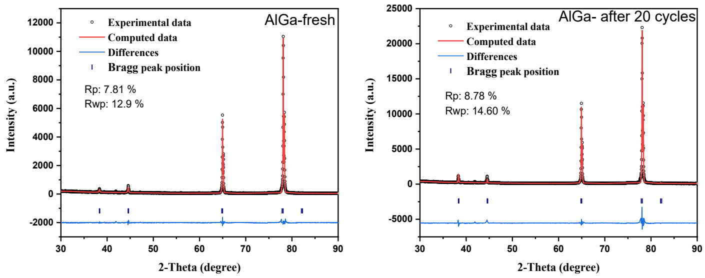

c

	a(Å)	b(Å)	c(Å)
Al-fresh	4.06552	4.06552	4.06552
Al after 20cycles	4.06464	4.06464	4.06464

Figure S16 (a-b) Rietveld refinement of the fresh and after 20 cycles AlGa. (c) Refined lattice parameters obtained from X-ray diffraction Rietveld refinement.

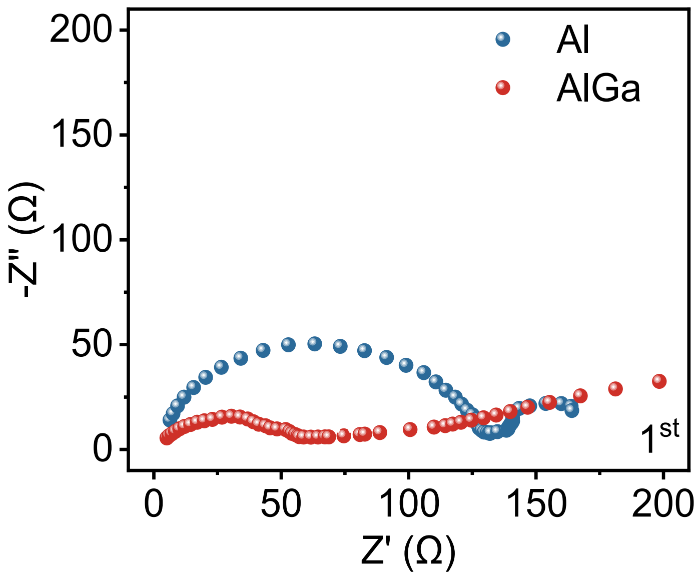

Supplementary Fig. 17 The electrochemical impedance spectra curves of the asymmetric battery after first cycle.

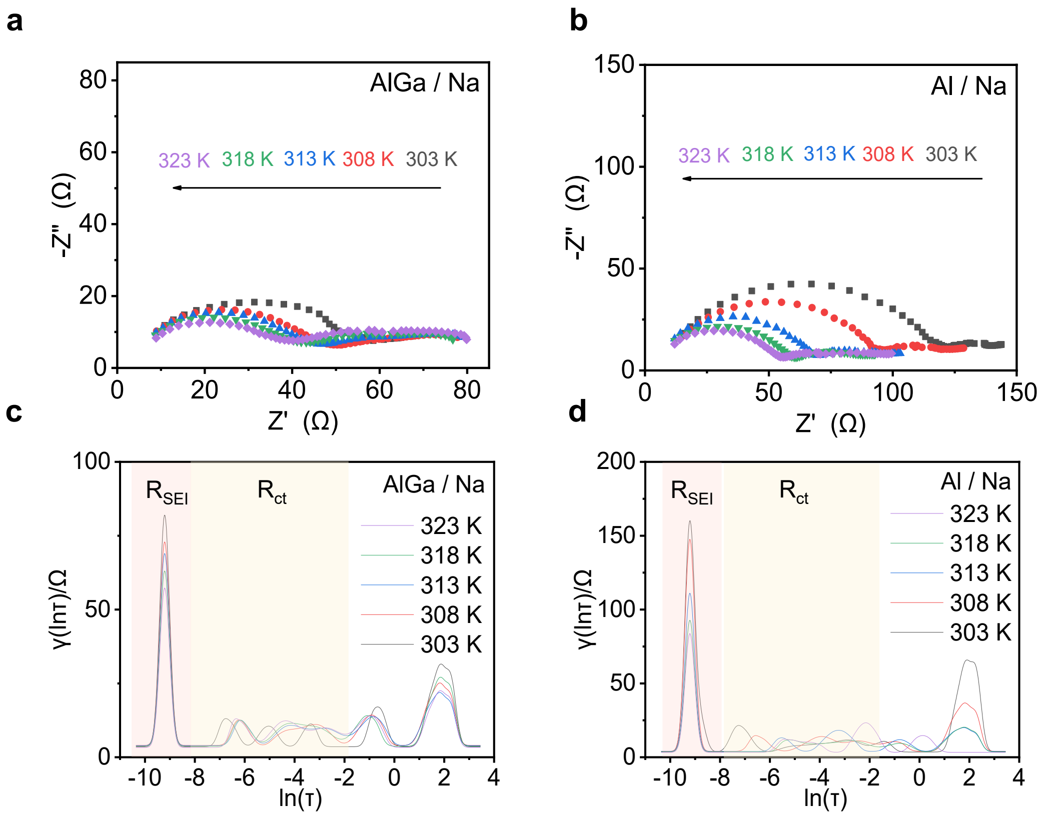

Supplementary Fig. 18 The nyquist plots and the corresponding DRT analyses of AlGa/Na (a, c) and Al/Na (b, d) based symmetric batteries at different temperatures (plating Na 1 mA cm^{-2} / 1 mAh cm^{-2}).

By integrating the DRT peaks over specific relaxation time ranges, the resistance contributions associated with the SEI layer (R_{SEI}, τ : 10^{-6}–10^{-4} s) and charge transfer processes (R_{ct}, τ : 10^{-3}–10^{-1} s) were quantitatively extracted[1, 2]. Both R_{SEI} and R_{ct} decrease monotonically with increasing temperature, which is consistent with thermally activated ion transport and charge transfer processes following Arrhenius-type behavior.

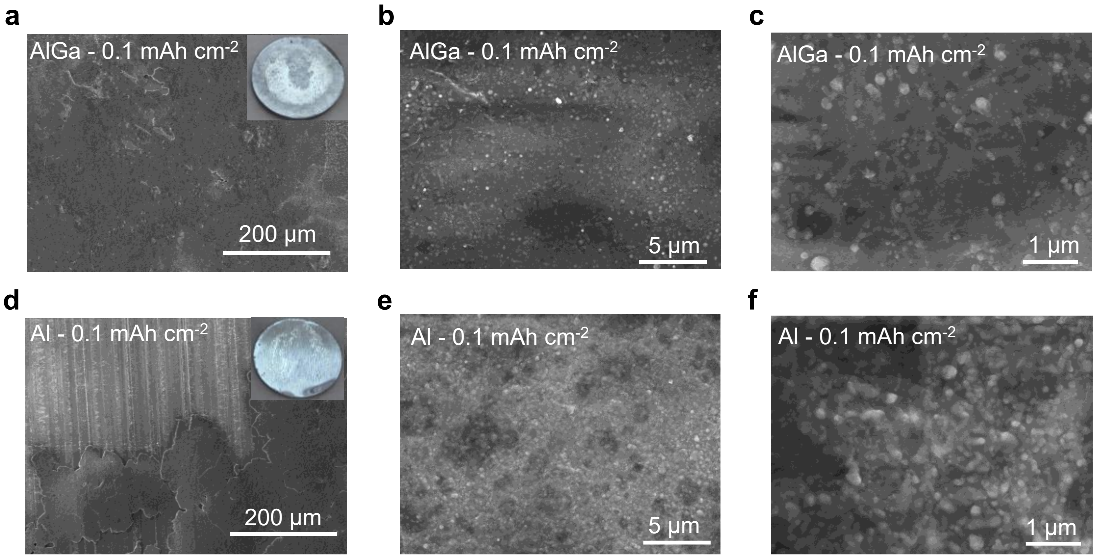

Supplementary Fig. 19 SEM images of Na deposition on AlGa (a-c) and Al (d-f) at 1 mA cm^{-2} for 0.1 mAh cm $^{-2}$. The corresponding optical images are shown in the upper right corners of Fig. a and d.

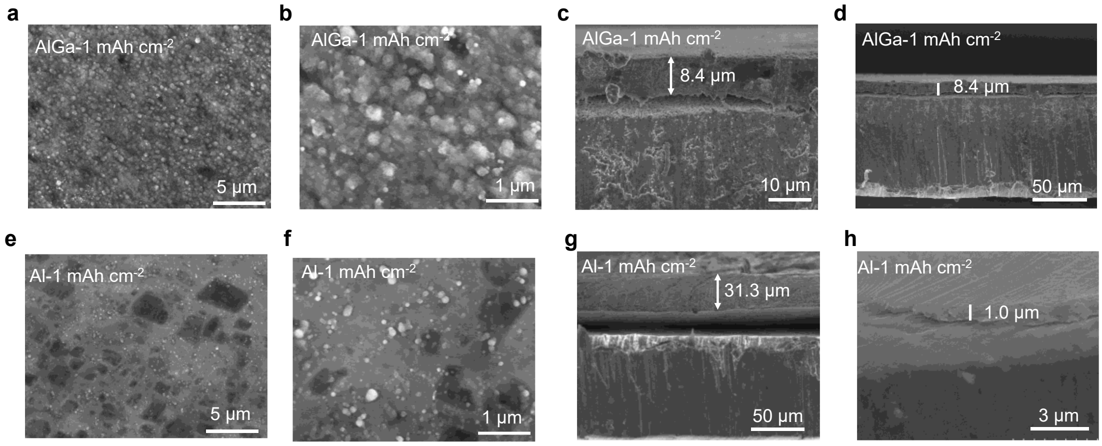

Supplementary Fig. 20 SEM images of Na deposition on AlGa(a-d) and Al(e-h) at 1 mA cm^{-2} for 1 mAh cm^{-2}.

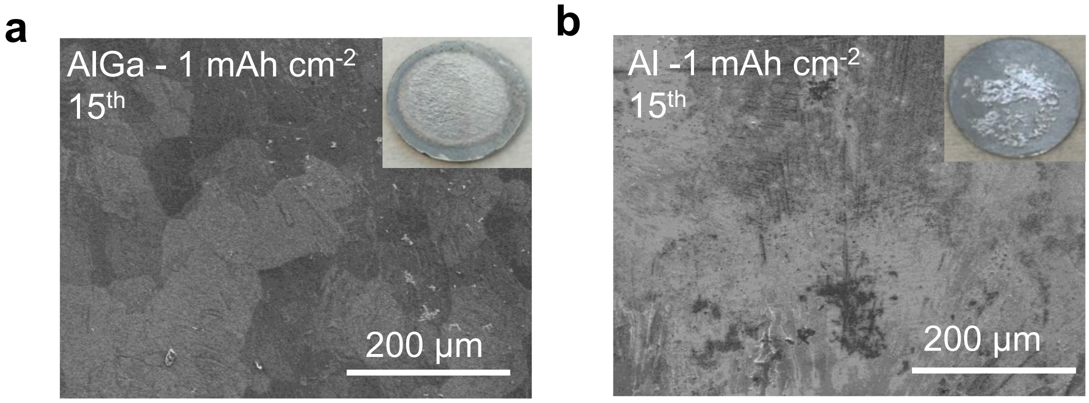

Supplementary Fig. 21 SEM images of Na deposition on AlGa (a) and Al (b) after 15 cycles at 1 mA cm^{-2} for 1 mAh cm^{-2}. The corresponding optical images are shown in the upper right corners.

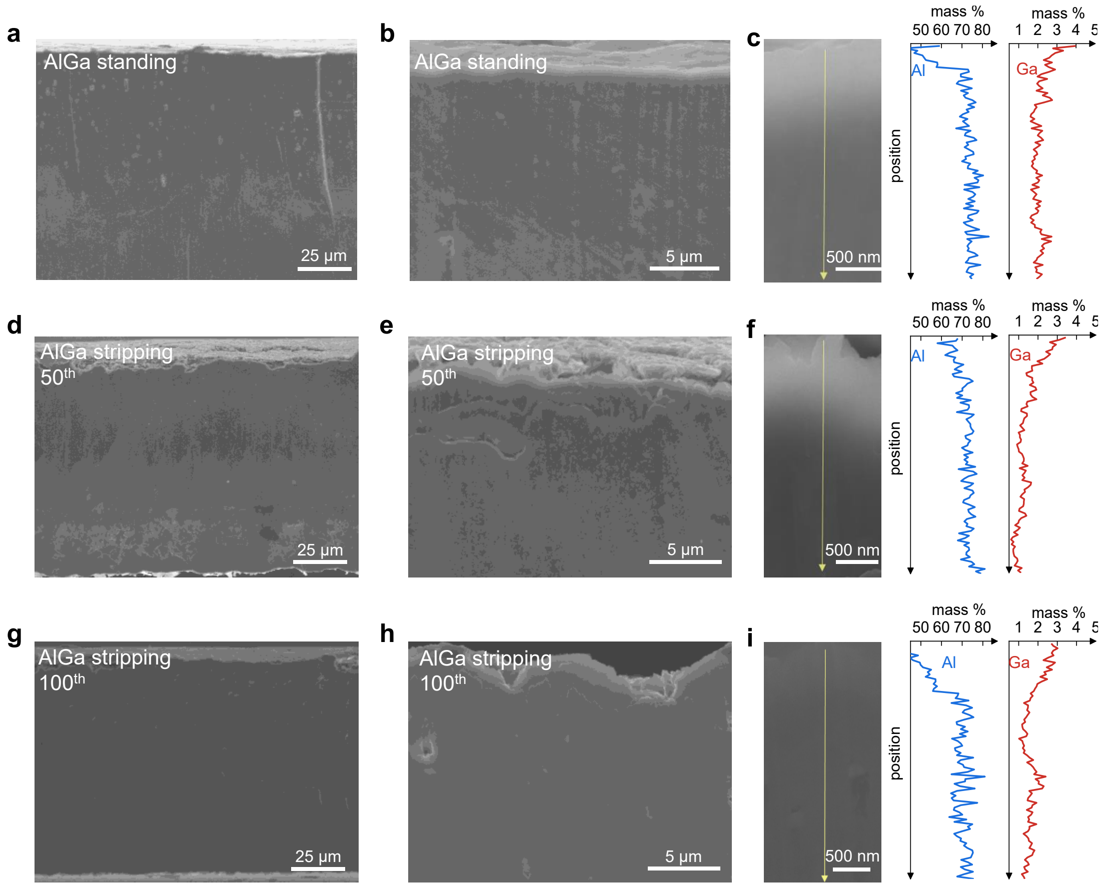

Supplementary Fig. 22 Cross-sectional SEM images of AlGa after 8 days of standing (a, b) and the corresponding EDS line scan of Al and Ga (c), showing an atomic ratio of 71.98:1.24. After 50 cycles (d, e), the corresponding line scan (f) shows an Al:Ga ratio of 66.74:0.90. After 100 cycles (g, h), the line scan (i) shows an Al:Ga ratio of 69.19:1.10.

AlGa standing

a

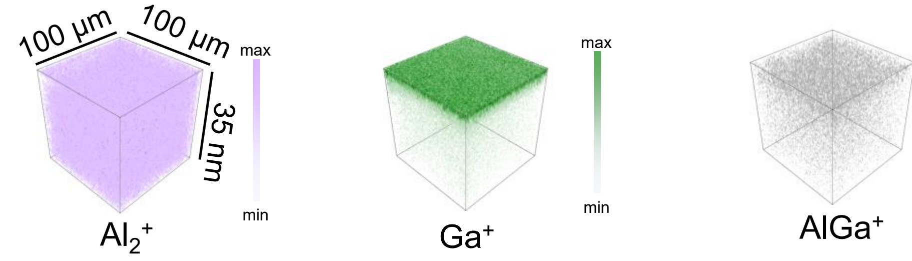

b

**Instrument:** IONTOF M6-Hybrid	
**Acquisition phase**	
**Ion species**	**Bi1 (positive mode)**
**Energy**	**30 keV**
**Ion current**	**0.870 pA**
**Raster size**	**100 μm x 100 μm**
**Mass range**	**0 - 270 u**
**Mode**	**All-Purpose (Hybrid)**
**Analysis time**	**300 s (spectra)**
**Sputter beam**	
**Ion species**	**Ar**
**Energy/Current**	**500 eV / 66.130 nA**
**Raster size**	**300 μm x 300 μm**
**Sputtering time**	**300 sec**

d

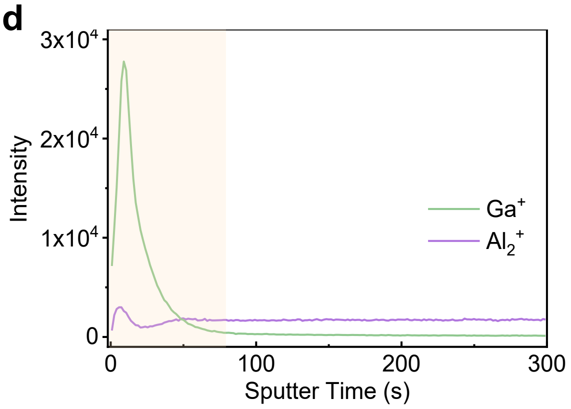

c

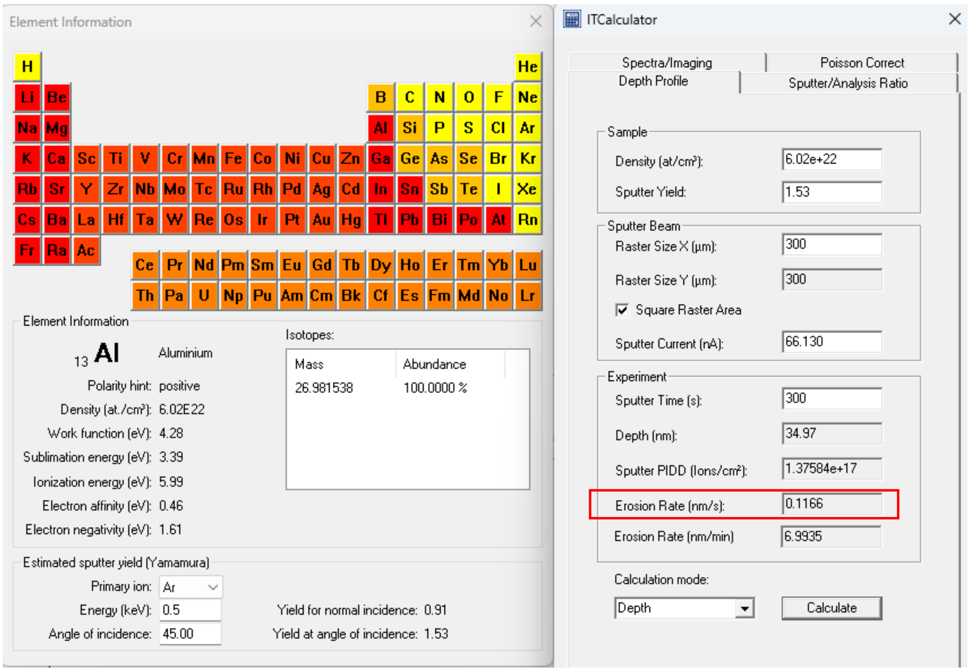

Supplementary Fig. 23 (a) TOF-SIMS depth profiles of AlGa after 8 days of standing. (b) Actual conditions of TOF-SIMS testing. (c)The variation of fragment species intensity with sputtering time. (d) Calculation of the Al etching rate corresponding to the set conditions within the instrument. For metal Al, the etching rate is 0.1166 nm s^{-1}. At an etching time of approximately 80 s, there is no significant change in Ga^+ ions. Therefore, the thickness of the Ga layer is estimated to be approximately 9.3 nm (0.1166 × 80 = 9.3).

AlGa stripping 50th

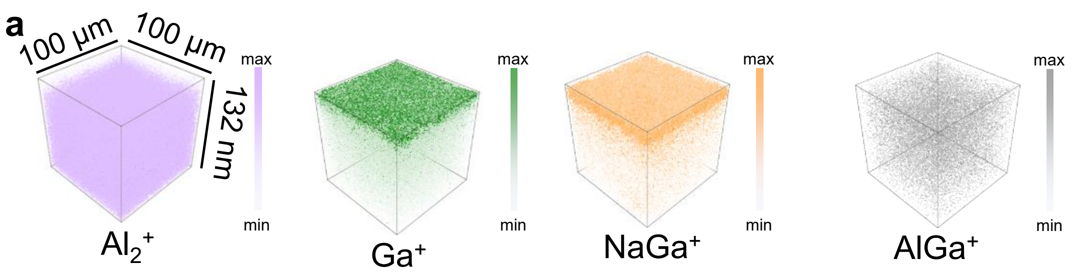

b

Instrument: IONTOF M6-Hybrid	
**Acquisition phase**	
Ion species	Bi1 (positive mode)
Energy	30 keV
Ion current	0.900 pA
Raster size	100 μm x 100 μm
Mass range	0 - 270 u
Mode	All-Purpose (Hybrid)
Analysis time	1147 s (spectra)
**Sputter beam**	
Ion species	Ar
Energy/Current	500 eV / 65.240 nA
Raster size	300 μm x 300 μm
Sputtering time	1147 sec

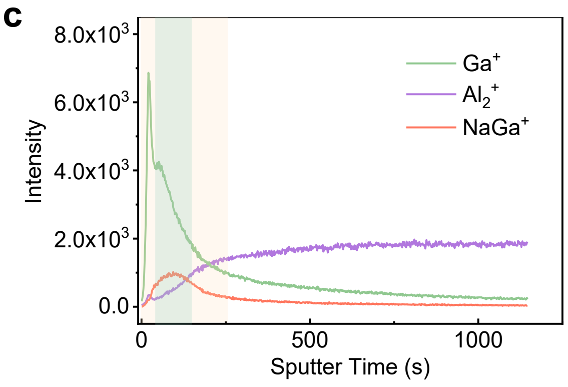

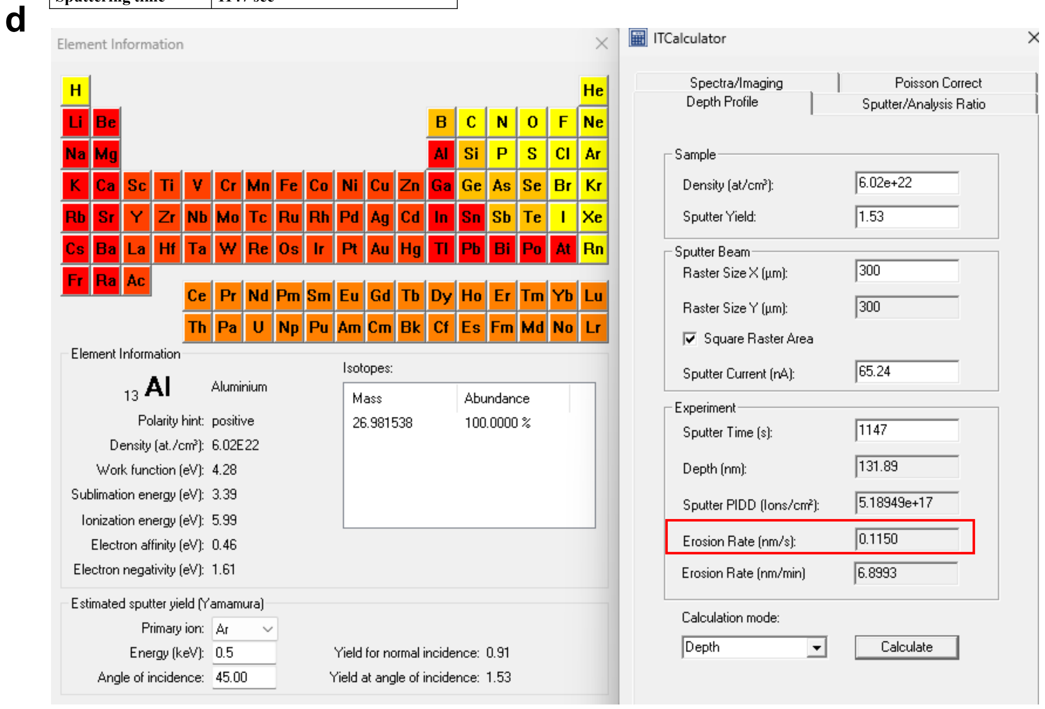

Supplementary Fig. 24 (a) TOF-SIMS depth profiles of AlGa after 50 cycles. (b) Actual conditions of TOF-SIMS testing. (c)The variation of fragment species intensity with sputtering time. (d) Calculation of the Al etching rate corresponding to the set conditions within the instrument. For metal Al, the etching rate is 0.1150 nm s^{-1}. At an etching time of approximately 250 s, there is no significant change in Ga^+ ions. Therefore, the thickness of the Ga layer is estimated to be approximately 9.3 nm (0.1150 × 250 = 28.8 nm).

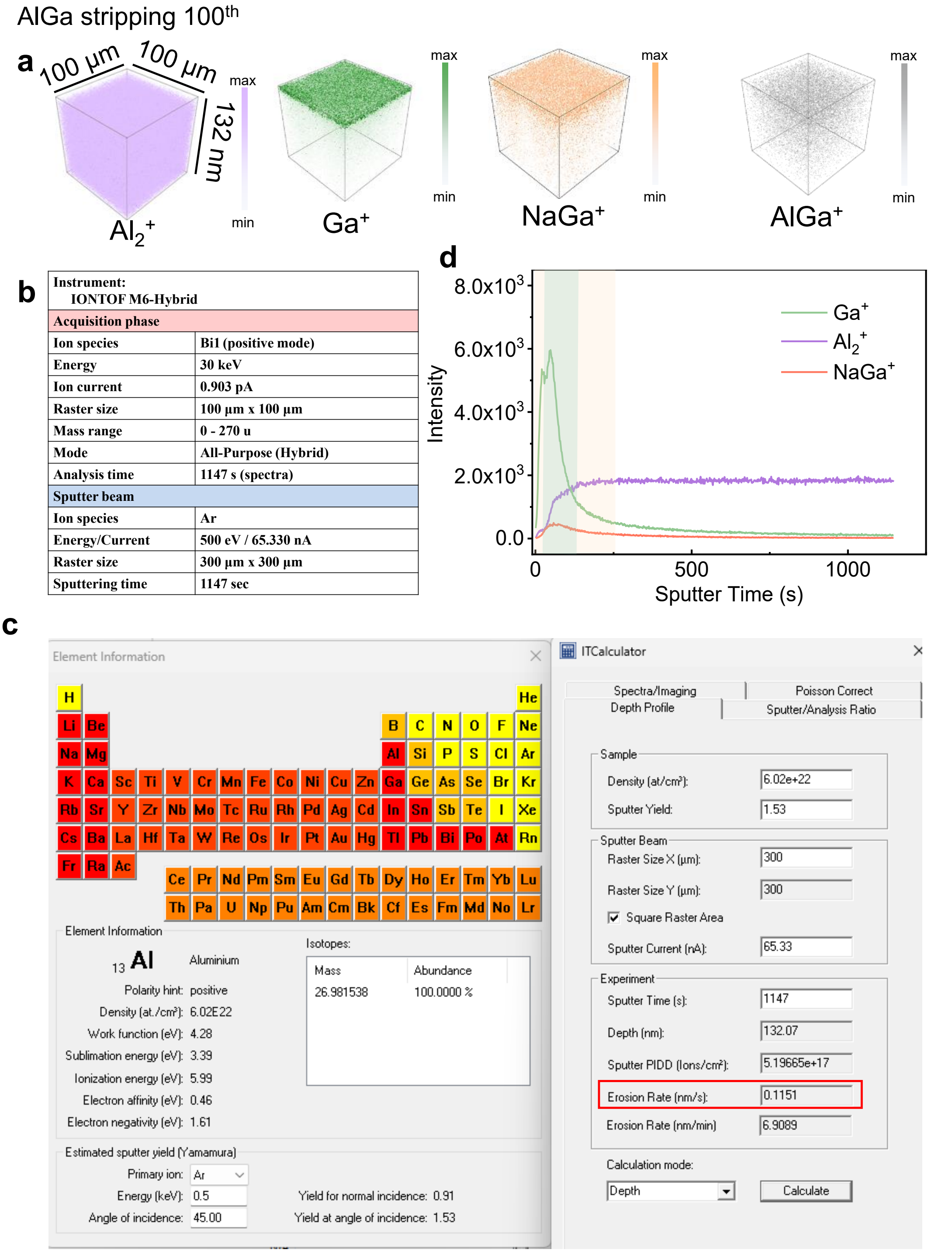

Supplementary Fig. 25 (a) TOF-SIMS depth profiles of AlGa after 100 cycles. (b) Actual conditions of TOF-SIMS testing. (c)The variation of fragment species intensity with sputtering time. (d) Calculation of the Al etching rate corresponding to the set conditions within the instrument. For metal Al, the etching rate is 0.1151 nm s^{-1}. At an etching time of approximately 250 s, there is no significant change in Ga^+ ions. Therefore, the thickness of the Ga layer is estimated to be approximately 28.8 nm (0.1151 × 250 = 28.8 nm).

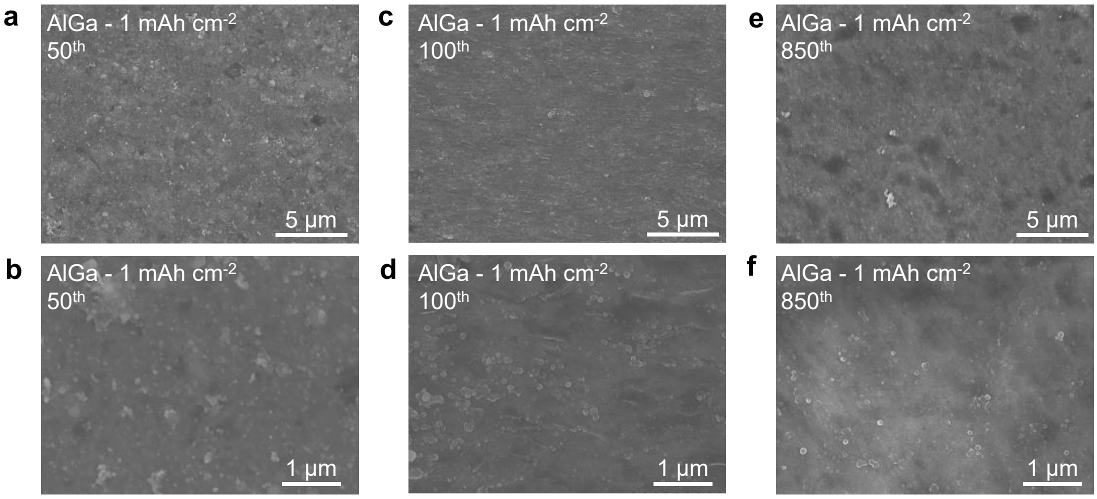

Supplementary Fig. 26 SEM images of Na deposition on AlGa at 1 mA cm^{-2} for 0.1 mAh cm^{-2} after 50(a-b), 100(c-d), 850(e-f) cycles.

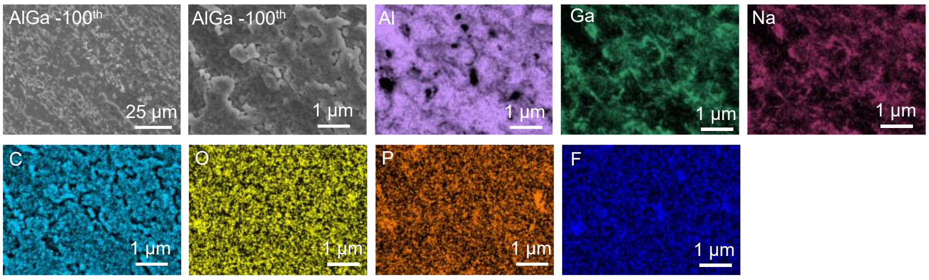

Element	Atom(%)
Al	62.12
Ga	2.64
Na	0.78
C	22.38
O	8.94
P	0.01
F	3.13
total	100

Supplementary Fig. 27 SEM images of Na deposition AlGa after 100 cycles at 1mA cm^{-2} for 1 mAh cm^{-2}

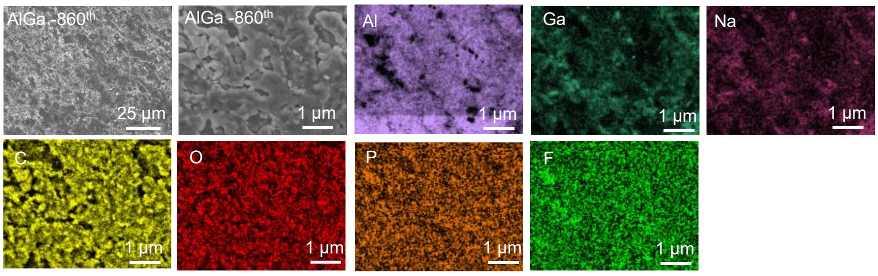

Element	Atom(%)
Al	64.80
Ga	1.85
Na	0.66
C	21.21
O	8.51
P	0.00
F	2.96
total	100

Supplementary Fig. 28 SEM images of Na deposition AlGa after 860 cycles at 1mA cm^{-2} for 1 mAh cm^{-2}

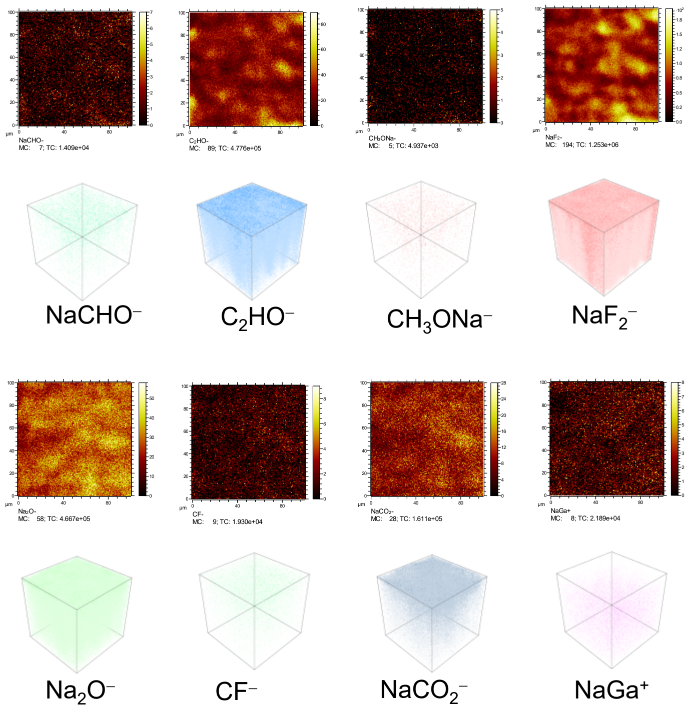

Instrument: IONTOF M6-Hybrid	
Acquisition phase	
Ion species	Bi3 (positive mode)
Energy	30 keV
Ion current	0.517 pA
Raster size	100 µm x 100 µm
pixel dimensions	128 x 128
Mass range	0 - 270 u
Mode	All-Purpose (Hybrid)
Analysis time	600 s (spectra)
Sputter beam	
Ion species	Ar
Energy/Current	500 eV / 65.020 nA
Raster size	300 µm x 300 µm
Sputtering time	600 sec

Instrument: IONTOF M6-Hybrid	
Acquisition phase	
Ion species	Bi3 (negative mode)
Energy	30 keV
Ion current	0.518 pA
Raster size	100 µm x 100 µm
pixel dimensions	128 x 128
Mass range	0 - 270 u
Mode	All-Purpose (Hybrid)
Analysis time	600 s (spectra)
Sputter beam	
Ion species	Ar
Energy/Current	500 eV / 64.560 nA
Raster size	300 µm x 300 µm
Sputtering time	600 sec

Supplementary Fig. 29 TOF-SIMS 3D reconstruction of the sputtered volume of several secondary ion fragments on the cycled AlGa surface.

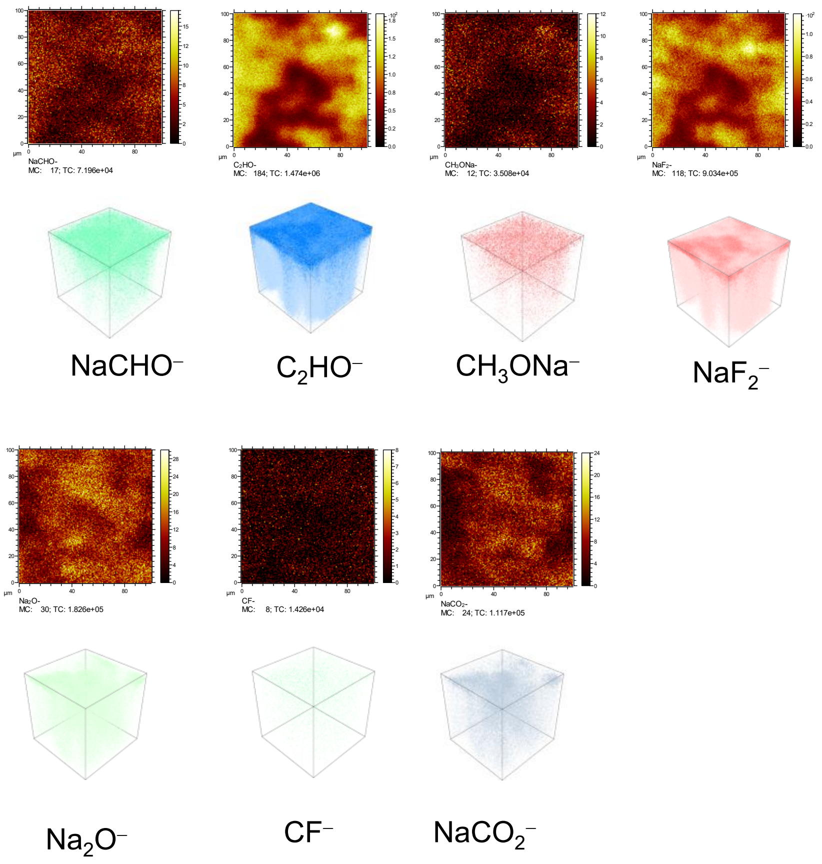

Instrument: IONTOF M6-Hybrid	
Acquisition phase	
Ion species	Bi3 (positive mode)
Energy	30 keV
Ion current	0.511 pA
Raster size	100 µm x 100 µm
pixel dimensions	128 x 128
Mass range	0 - 270 u
Mode	All-Purpose (Hybrid)
Analysis time	600 s (spectra)
Sputter beam	
Ion species	Ar
Energy/Current	500 eV / 65.300 nA
Raster size	300 µm x 300 µm
Sputtering time	600 sec

Supplementary Fig. 30 TOF-SIMS 3D reconstruction of the sputtered volume of several secondary ion fragments on the cycled Al surface.

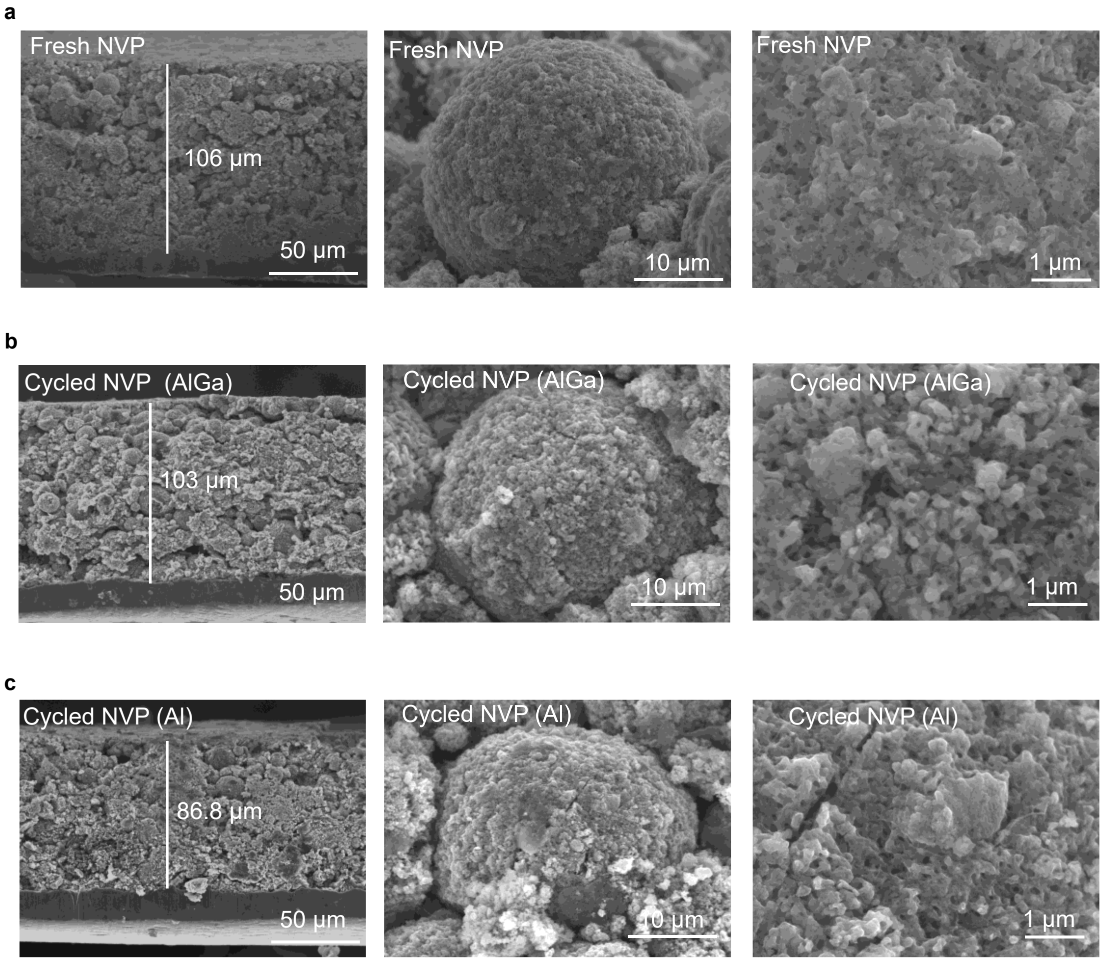

Supplementary Fig. S31 The cross-sectional SEM images of the fresh NVP(a), and NVP after cycled with AlGa (b) and Al (c). The NVP positive electrode massing loading is 11.4 mg cm^{-2}

a

b

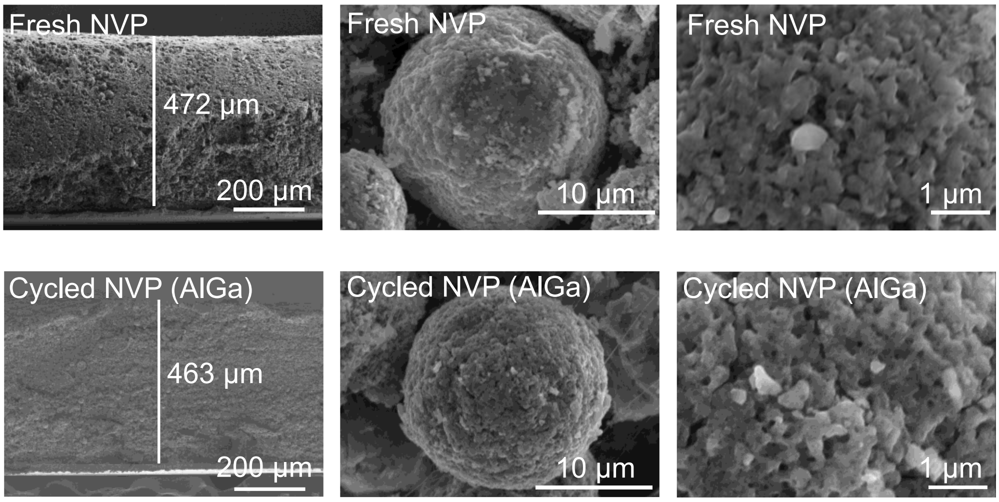

Supplementary Fig. 32 The cross-sectional SEM images of the fresh NVP(a), and NVP after cycled with AlGa (b). The NVP positive electrode massing loading is 47.4 mg cm^{-2}

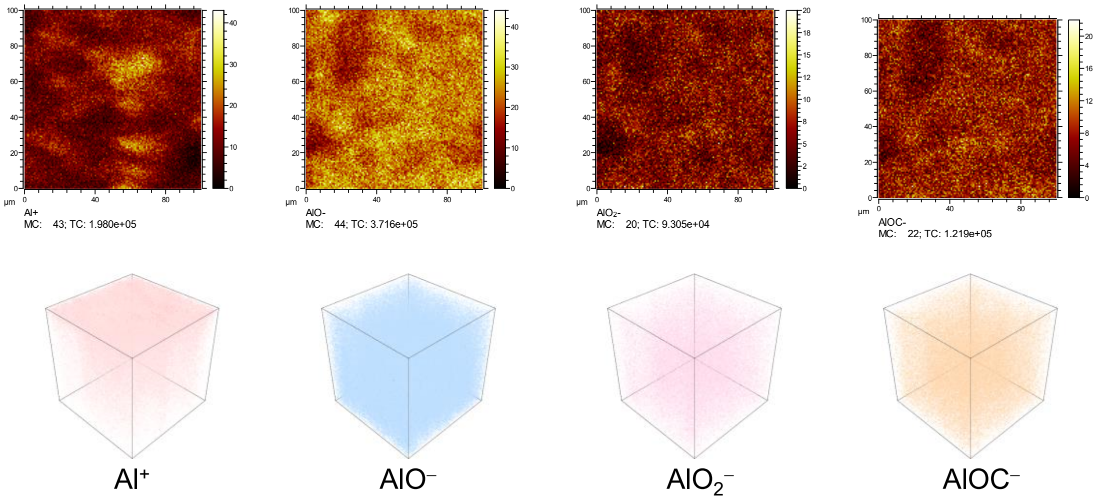

Instrument: IONTOF M6-Hybrid	
Acquisition phase	
Ion species	Bi3 (positive mode)
Energy	30 keV
Ion current	0.522 pA
Raster size	100 µm x 100 µm
Mass range	0 - 270 u
Mode	All-Purpose (Hybrid)
Analysis time	600 s (spectra)
Sputter beam	
Ion species	Ar
Energy/Current	500 eV / 62.12 nA
Raster size	300 µm x 300 µm
Sputtering time	600 sec

Instrument: IONTOF M6-Hybrid	
Acquisition phase	
Ion species	Bi3 (negative mode)
Energy	30 keV
Ion current	0.519 pA
Raster size	100 µm x 100 µm
Mass range	0 - 270 u
Mode	All-Purpose (Hybrid)
Analysis time	600 s (spectra)
Sputter beam	
Ion species	Ar
Energy/Current	500 eV / 62.460 nA
Raster size	300 µm x 300 µm
Sputtering time	600 sec

Supplementary Fig. 33 TOF-SIMS 3D reconstruction of the sputtered volume of several secondary ion fragments on the cycled NVP matched with AlGa surface.

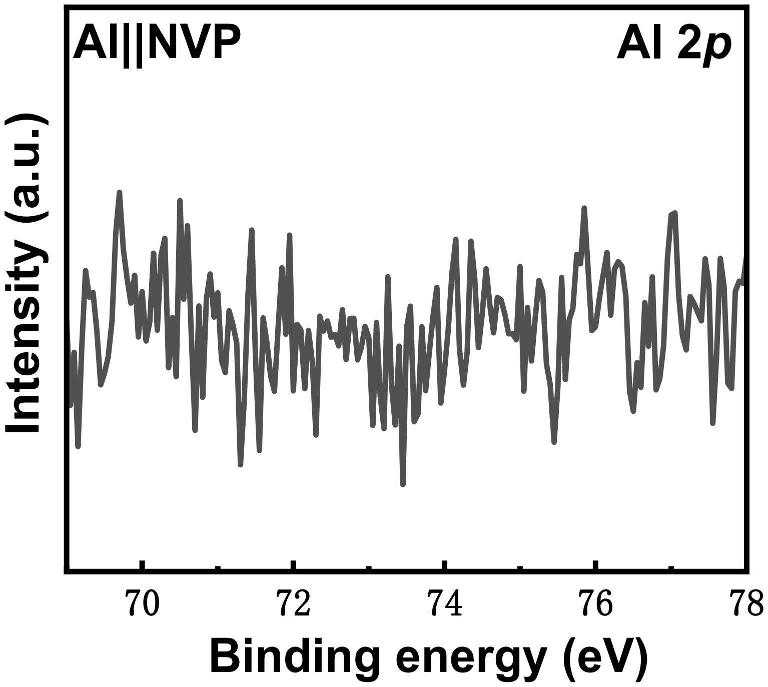

Supplementary Fig. 34 XPS spectra of Al 2*p* for the surface of NVP cycled with Al.

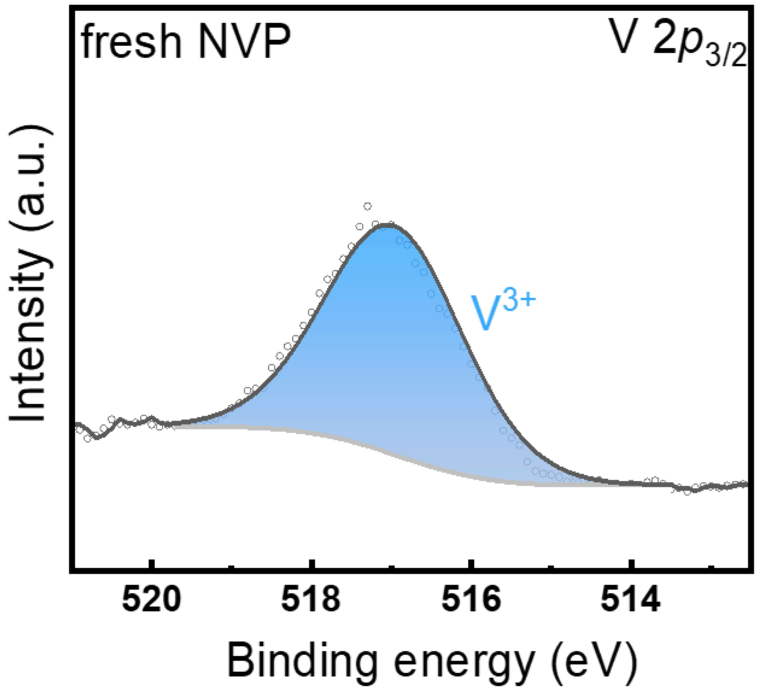

Supplementary Fig. 35 XPS spectra of V 2$p_{3/2}$ for the surface of fresh NVP

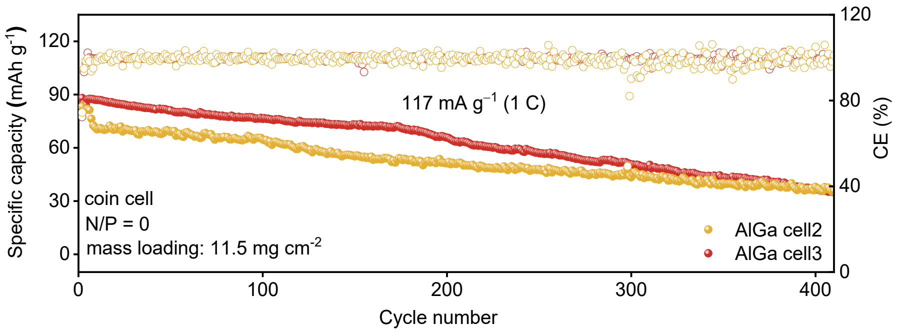

Supplementary Fig. 36 The cyclic stability of AlGa||NVP and Al||NVP cells at 1 C, 1 C = 117 mAh g^{-1}.

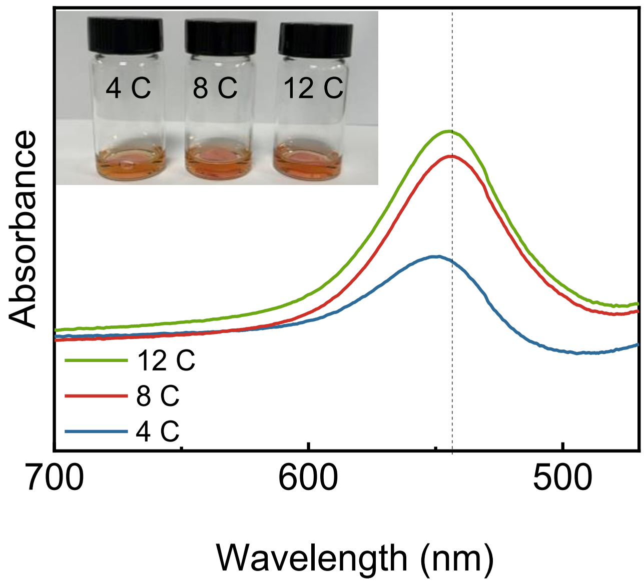

Supplementary Fig. 37 UV-vis spectra of electrolyte-infused PP separators from AlGa||NVP at different current density (4 C, 8 C, 12 C) for 20 cycles reacted with Al reagent solution. Inset is Optical images.

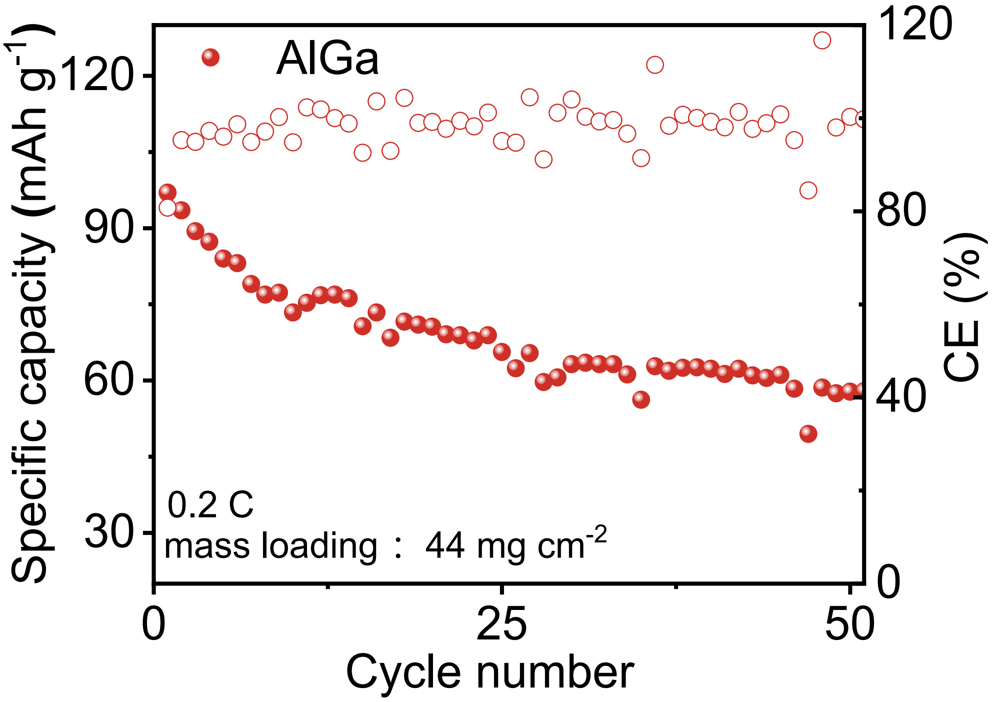

Supplementary Fig. 38 Cyclic stability of AlGa||NVP initially anode-free cells at 0.2 C.

Supplementary Table 1 ICP-OES test result.

Sample	Al element concentrationin the test solution (mg/L)
Diglyme	0.193
After cycled electrolyte	5.558

Based on ICP-OES results, the Al concentration in the electrolyte after cycling is 5.580 mg L^{-1}, while the Al concentration in the pristine diglyme is 0.193 mg L^{-1}. After subtracting the background contribution, the net Al concentration is 5.387 mg L^{-1}. This value corresponds to a sample obtained by diluting 30 μL of electrolyte to 2 mL. Therefore, the original Al concentration in the electrolyte is calculated to be 0.359 g L^{-1} ($5.387 \times 2000 / 30 = 359.1$ mg L^{-1} = 0.359 g L^{-1}).Given the molar mass of Al (27 g mol^{-1}), the corresponding molar concentration is approximately 0.013 mol L^{-1} ($0.359 / 27 \approx 0.013$ mol L^{-1}).

The total amount of dissolved Al in 30 μL electrolyte is thus 3.9×10^{-7} mol (0.013 mol $L^{-1} \times 30$ μL), corresponding to a mass of 10.5 μg ($3.9 \times 10^{-7} \times 27 = 1.05 \times 10^{-5}$ g). Using the density of Al ($\rho = 2.70$ g cm^{-3}), the corresponding volume of dissolved Al can be estimated. For an electrode with a diameter of 12 mm (area ≈ 1.13 cm^2), the equivalent thickness is calculated to be ~34 nm ($1.05 \times 10^{-5} / 2.7 / 1.13 \approx 3.4 \times 10^{-6}$ cm ≈ 34 nm).

Supplementary Table 2 Parameter for the 201.50 Wh kg^{-1} pouch cell

Parameter		Value
Positive electrode areal capacity		5.54 mAh cm^{-2}
N/P ratio		0
Discharge capacity		100.18 mAh g^{-1}
Average discharge voltage		3.36 V
size		5 cm × 4 cm
Assigned density	NVP	47.40 mg cm^{-2}
	AlGa foil	8.05 mg cm^{-2}
	Electrolyte	30.00 mg cm^{-2}
	Separator	1.03 mg cm^{-2}
	Al collector	4.10 mg cm^{-2}
	Conductive adhesive	1.80 mg cm^{-2}
	Total	92.38 mg cm^{-2}
Specific energy		201.50 Wh kg^{-1}

The pouch cell is of a single-layer configuration (i.e., one positive electrode, one negative electrode, and one separator), and the contribution of packaging materials is not included in the calculation.

Supplementary Table 3 The hardness (H-IT) of Al, AlGa, H-AlGa, Al-800°C and AlGa after 100 cycled.

Sample	H-IT (Gpa)	Average H-IT (Gpa)
Al	0.807	0.804
	0.783	
	0.821	
AlGa	0.280	0.314
	0.262	
	0.401	
H-AlGa	0.572	0.586
	0.566	
	0.621	
Al-800°C	0.543	0.521
	0.507	
	0.515	
AlGa after 100 cycled	0.373	0.311
	0.229	
	0.332	

The H-IT data were determined in accordance with ISO 14577.

To further rule out the possibility that the hardness reduction is caused by the enrichment of gallium at the grain boundaries and its dissolution in the aluminum lattice (solid solution), we prepared a control sample (labeled as H-AlGa) by annealing aluminum-gallium at 800°C for 5 hours to promote the complete diffusion of gallium into the aluminum lattice. Considering that the heat treatment itself may affect the hardness, we also tested pure aluminum foil (labeled as Al-800°C) after the same annealing treatment for comparison.

The results show that the hardness of both H-AlGa and Al-800°C is significantly higher than that of the prepared aluminum-gallium samples, indicating that the uniform dissolution of gallium in the aluminum lattice cannot explain this significant softening phenomenon. Notably, after 100 cycles, the hardness of the AlGa sample is still comparable to the original AlGa and is significantly lower than H-AlGa and Al-800°C.

In addition to the preferential distribution of Ga along grain boundaries, the nanoindentation results may also reflect the combined effects of residual stress, surface Al dissolution, and SEI coverage. Nevertheless, the largely unchanged surface hardness after cycling further confirms the structural stability.

Supplementary Table 4 The conductivity of Al and AlGa.

sample	Conductivity ($S\cdot m^{-1}$)	Average conductivity ($S\cdot m^{-1}$)
Al	2.55×10^7	2.64×10^7
	2.66×10^7	
	2.71×10^7	
AlGa	2.13×10^7	2.12×10^7
	2.10×10^7	
	2.14×10^7	

Supplementary Table 5 Comparison of our work with recent works on asymmetric cells.

Current collector	Asymmetric cells			electrolyte	Separator	Ref.	note
	Current density (mA cm^{-2})	Areal capacity (mAh cm^{-2})	Cycle number				
AlGa	1	1	2500	1M $NaPF_6$ in diglyme	PP	This work	
					PP	This work	
single-crystal Al(100)	2	2	500	1 M $NaPF_6$ in DME	GF/D	Nat. Commun. 2025 *16*:2280	
zinc flower matrix on Al	1	1	1100	0.9 M $NaPF_6$ + 0.1 M $NaBF_4$ in diglyme	PP	Adv. Mater. 2024, 2413253	
high-entropy alloy (NbMoTaWV@Al)	2	2	1000	1 M $NaPF_6$ in DME	–	Adv. Mater. 2024, 2413331	
carbon particle containing Fe nanoparticles	1	2	1500	1M $NaPF_6$ in diglyme	glass fiber	Adv. Mater. 2022, *34*, 2109767.	
carbon nanosheet with SnO_2 nanoparticles	2	1	2100	1M $NaPF_6$ in diglyme	glass fiber	Adv. Funct. Mater. 2025, 2502032	
fluorinated covalent triazine framework	2	1	400	1M $NaPF_6$ in diglyme	glass fiber	Sci. Adv. 2023, *9*, eadh8060	
Ru modified-Cu	2	2	1000	1M $NaPF_6$ in diglyme	GF/F	Angew. Chem. Int. Ed. 2025, e202503691	Pre-cycles for 5 times
carbon macroporous fibers incorporated with CoP nanoparticles	1	1	480	1M $NaPF_6$ in diglyme	Glass fiber	Sci. Adv. 2025, *11*, eadv2007	

Cu_3P@Cu	1	1	800	1M $NaPF_6$ in diglyme	—	Adv. Mater. 2024, *36*, 2310347.	

Supplementary Table 6 The R_{SEI} and R_{ct} results obtained by fitting based on the DRT results

AlGa		Al	
R_{SEI} (Ω)	R_{ct} (Ω)	R_{SEI} (Ω)	R_{ct} (Ω)
38.51	29.21	85.24	39.71
33.83	25.21	70.49	33.00
31.92	23.04	52.60	26.23
29.13	21.11	43.71	25.83
26.49	19.05	39.46	24.29

Supplementary Table 7 Comparison of our work with recent works on full cells.

Current collector	Full cells (N / P = 0)				electrolyte	Seperator	Current density (mA cm^{-2})	Ref.	note
	Positive electrode	Mass loading (mg cm^{-2})	Cycle number	capacity retention					
AlGa	NVP (pouch cell)	11.4	100	88.7%	1M $NaPF_6$ in diglyme	PP	0.45	This work	
	NVP (coin cell)	11.5	400	50%		PP	1.35	This work	
graphitic carbon-coated current collector	NFM (coin cell)	15.0	260	84%	0.9 M NaPF6+0.1 M NaBF4 in diglyme	GF/A+PP	0.952	Nat. Energy. 2022,*7*, 511-519	
single-crystal Al(100)	NVP (coin cell)	1.5	200	~85%	1 M $NaPF_6$ in DME	GF/D	0.79	Nat. Commun. 2025, *16*, 2280	
zinc flower matrix on Al	NVP (coin cell)	32	100	86%	0.9 M NaPF6+0.1 M NaBF4 in diglyme	PP	0.37	Adv. Mater. 2024, 2413253	
high-entropy alloy (NbMoTaWV@Al)	NVP (coin cell)	1.8	300	98%	1 M $NaPF_6$ in DME	—	0.21	Adv. Mater. 2024, 2413331	
Fluorination of annealed Al	NVP(coin cell)	11.3	50	46.1%	30 mol% NaFSA-[C2C1im][FSA]	GF/A	0.44	Adv. Energy Mater. 2023, *13*, 2302468.	

carbon particle containing Fe nanoparticles	NVP(coin cell)	10	100	97%	1M $NaPF_6$ in diglyme	glass fiber	1.00	Adv. Mater. 2022, *34*, 2109767.	pre-sodium-treated.
carbon nanosheet with SnO_2 nanoparticles	NVP (coin cell)	3.5	200	79.5%	1M $NaPF_6$ in diglyme	glass fiber	0.35	Adv. Funct. Mater. 2025, 2502032	
Ru modified-Cu	NVP (coin cell)	18	100	98.1%	1M $NaPF_6$ in diglyme	GF/F	1.05	Angew. Chem. Int. Ed. 2025, e202503691	Pre-cycles for 5 times
mesoporous carbon fibers	NVP (coin cell)	20	200	90%	1M $NaPF_6$ in diglyme	GF/D	0.78	Nat. Commun. 2025, *16*, 5494.	

Supplementary References

1. Wan TH, Saccoccio M, Chen C, Ciucci F. Influence of the Discretization Methods on the Distribution of Relaxation Times Deconvolution: Implementing Radial Basis Functions with DRTtools.Electrochim. Acta 184, 483-499 (2015).
2. Maradesa A, et al. Advancing electrochemical impedance analysis through innovations in the distribution of relaxation times method. Joule 8, 1958-1981 (2024).
