# Supplementary material for: Grain boundary engineered aluminum current collector for energy-dense initially anode-free sodium metal batteries
Source: Nat Commun. 2026 Jun 6;17:7225. doi: 10.1038/s41467-026-74177-8 (PMC13396730; doi:10.1038/s41467-026-74177-8)
Supplement: Supplementary file 2 — Transparent Peer Review file [file 41467_2026_74177_MOESM2_ESM.pdf]

nature portfolio

Peer Review File

Grain boundary engineered aluminum current collector for energy-dense initially anode-free sodium metal batteries

Corresponding Author: Professor Danni Lei

This file contains all reviewer reports in order by version, followed by all author rebuttals in order by version.

Version 0:

Reviewer comments:

Reviewer #1

(Remarks to the Author)
The manuscript reports a strategy for stabilizing sodium deposition through a hierarchically alloyed current collector. The authors utilize a grain-boundary gallium-rich polycrystalline aluminum current collector (AlGa) to trigger controlled dissolution of Al3+ and localized charge redistribution. The pouch cells are constructed by coupling AlGa with commercial Na3V2(PO4)3. However, I have several critical concerns over this work, which I believe preclude its further consideration for acceptance in Nature Communications.
1. Gallium metal has a melting point of 29.76 °C, rendering it highly susceptible to liquefaction. This tendency is further exacerbated when forming alloys, which can lower their melting point. Consequently, the question arises as to how the gallium layer can be assured of stable existence upon the Al current collector in this manuscript.
2. Gallium metal also forms alloys with sodium metal, causing volume expansion and internal stress that adversely affect the long-cycle stability of current collectors, particularly in anode-free configurations. However, the authors did not mention anything about this issue in the manuscript.
3. The author mentioned that “Further, the Ga penetration along the grain boundaries generates amorphous regions, which weaken the Al crystal structure and facilitate Al atoms gradual dissolution.” How can the stability of the collector be ensured over extended cycles when Al current collectors are subject to continuous corrosion? The manuscript only presents SEM images after 15 cycles. The morphology and phase distribution of the alloy current collector should be characterized after longer cycling (e.g., 1000 cycles).
4. In Figure S17, the cross-sectional SEM images of Na deposition on the Al current collector also appear to be smooth and dense, which is inconsistent with the author's description (drastic inhomogeneous Na deposition). Please provide clearer planar and cross-sectional SEM images and explain why the uniform deposition thickness reaches 31 μm at a current density of 1 mA cm^{-2}.
5. The authors declare AlGa leading to a uniform SEI layer formation, but it does not provide a systematic analysis of its chemical composition and formation mechanism. Please supplement the test results and analyze the reasons for this result.
6. The capacity of AlGa||NVP cell in Figure 5c increases with rising current density (after 8C). What is the reason for this phenomenon? Is it because dissolved Al3+ ions participate in the battery reaction? If not, please explain the reason of the sudden increase in capacity.

Reviewer #2

(Remarks to the Author)
This manuscript addresses the critical issue of rapid active Na depletion in anode free sodium metal batteries by presenting a cutting-edge strategy, which holds great relevance and timeliness for the advancement of post-lithium battery technologies. The AlGa collector design demonstrates innovation and feasibility for mass production, and the superior performance of both Na||AlGa and AlGa||NVP cells is commendable. Additionally, the proposed mechanism of charge compensation offers useful insights for collector engineering, making the work likely to interest battery researchers. To further enhance the manuscript’s quality, a few issues need clarification and improvement. Therefore, I recommend accepting this manuscript once minor revisions are completed.
1.The authors verified the dissolution of Al^{3+} from AlGa. I wonder if Al^{3+} in AlGa will be continuously released during the battery cycling process.
2.In Fig.2c, the distribution of C and O elements appears to correspond to that of Ga, what is their origin?

3.In Fig.5a, what are the reasons for the slight fluctuation in the Coulombic efficiency of the battery?
4.In Fig.5b, there are fluctuations in the charge-discharge curve around 3.4 V, please explain the reason.
5.Please specify the sample preparation conditions and the specific transfer conditions in the Experimental Section.
6.There are numerous formatting issues in the manuscript, the authors are requested to carefully check and revise them. For example, “2wt%” should be “2 wt%”, “FigureS8” should be “Figure S8”.

Reviewer #3

(Remarks to the Author)
This work designs a current collector by coating Ga onto Al foil (AlGa) for anode-free Na batteries. The penetration of Ga into the grain boundaries of the Al foil induces the controlled dissolution of Al3+, thereby altering the local electrolyte charge distribution and triggering spontaneous Na+ migration from the bulk electrolyte to the cathode. As a result, the AlGa//Na cell demonstrates stable cycling performance for 2500 cycles at 1 mA cm-2 and 1 mAh cm-2. When paired with a Na3V2(PO4)3 cathode, the assembled anode-free cell realizes a high energy density exceeding 201.5 Wh kg-1. Overall, this work presents some valuable and insightful research findings. The experiments are well conducted in general, and the results are carefully analyzed and generally support the conclusions. Therefore, I would like to recommend the acceptance of this work after some revisions noted below.

1. The authors mention that enriching Ga at grain boundaries enhances battery performance. Can Ga at the grain boundaries maintain its stability during long-term cycling? Please clarify.
2. How does the introduction of Ga affect the intrinsic properties of the Al current collector, such as its mechanical property and electrical conductivity?
3. Figure 3h illustrates improved Na+ transport kinetics at the AlGa interface. To further substantiate this conclusion, the CV curves of AlGa//Na and Al//Na cells should be provided.
4. Figure S15 shows the Nyquist plots of AlGa/Na and Al/Na symmetric batteries at different temperatures. Please provide the equivalent circuit diagram to investigate the evolution of RSEI and Rct.
5. Figure S16 shows that the AlGa current collector is covered by Na metal at an areal capacity of 0.1 mAh cm-2. Please discuss in depth how the AlGa current collector continues to influence Na plating morphology under these conditions.
6. The anode-free AlGa||NVP cell with high mass loadings of the NVP cathode (~11.4 mg cm-2 and ~47.40 mg cm-2) demonstrates stable cycling performance. To better correlate performance with electrode structure, it is recommended to include cross-sectional SEM images of the NVP cathode, illustrating its thickness and morphology.
7. Please provide SEM image of the AlGa current collector after cycling in the anode-free Na battery to demonstrate its structural stability.
8. Some minor errors should be corrected.
In Figure 3d, “mAh cm-1” should be “mAh cm-2”
In Page 2 and 3, “… achieving simultaneous control over dendrite-free Na deposition and in situ self-replenishment of active Na remains …” should be “… achieving simultaneous control over dendrite-free Na deposition and in situ self-replenishment of active Na remain ...”
In Page 3, “The AlGa||Na exhibites …” should be “The AlGa||Na exhibits …”
In Page 3, “… which can easily extent to other anode-free battery systems” should be “… which can easily extend to other anode-free battery systems”
In Page 4, “The electrochemical processes on the Al and AlGa surface …” should be “The electrochemical processes on the Al and AlGa surfaces …”
In Page 5, “… the deposited Na on the Al and AlGa current collector …” should be “… the deposited Na on the Al and AlGa current collectors …”
In Page 7, “… elemental mapping tests (Figure 2b-c; Figure S5) shows … ” should be “… elemental mapping tests (Figure 2b-c; Figure S5) show … ”
In Page 17, “… the AlGa||NVP cell deliver …” should be “… the AlGa||NVP cell delivers …”
9. This direction of anode-free Na batteries is highly promising for future practical high-energy density batteries. Several highly related papers about functionalized Al collectors were most recently published, which do not undermine the novelty of this work significantly, for example, A functionalized porous Al current collector enables high-energy density anode-free Na batteries, Science Advances, 2025, 11, eadx7124; Growing Sodiophilic ZnO Nanorod Arrays on Al Substrate for High-Energy-Density Anode-Free Na Batteries, Journal of the American Chemical Society, https://doi.org/10.1021/jacs.5c16420

Reviewer #4

(Remarks to the Author)
The manuscript by Zheng et al. “Engineered Al Drives Na Self-Replenishment Realizes 200 Wh kg−1 Anode Free Batteries” discusses an interesting approach to preventing dendrite formation and dead Na in reservoir-free sodium ion batteries. To this end, the authors apply Ga to the aluminium current collector. This accumulates at the grain boundaries of the aluminium and, according to a cited paper, ensures that no covering layer of aluminium oxide forms in these areas. As a consequence, according to the authors' hypothesis, Al3+ ions are dissolved out of the current collector during cell cycling. The paper examines full cells with commercial NVP as the cathode material. It is demonstrated that the cells can operate for over 2500 cycles without short-circuiting. This is indeed a remarkable advance, although the charging rate of 0.3 C is very conservative.
The energy density achieved here is remarkably high for SIB and represents a noteworthy improvement and is worth being published in a high prestigious journal.
Although the connections are clearly illustrated with appropriate figures, the work has a few weaknesses that need to be

addressed before publication. I have the following comments, which are in the order in which they appear in the manuscript:
1) I cannot follow the argument of the introduction “The dissolution alters the local charge distribution in the electrolyte, thereby inducing spontaneous migration of Na^+ from the bulk electrolyte toward the cathode to compensate.” - Why should this happen? Al3+ can simply be dissolved at a lower rate due to its three times higher charge in comparison to Na+. Or instead, anions are attracted.
2) Please comment further on this statement “Further, the Ga penetration along the grain boundaries generates amorphous regions, which weaken the Al crystal structure and facilitate Al atoms gradual dissolution.” – Is Ga alloying with Al? If it is only adsorbed in the grain boundaries, the Al should not get amorphous.
3) If Al3+ is dissolved it might be also shuttling to the cathode. Can it be found in the cathode after cycling? – This might easily be proven by ToF-SIMS depth profiling, which is highly sensitive.
4) “Ga is mechanically coated onto Al foil” – How exactly was this carried out? I cannot find any information about this in the experimental section.
5) Figure 2c) Scale bars are missing
6) Figure 2d) No scale bars are given (dimensions and intensities (colour code)).
7) The experimental section has significant gaps in the materials characterisation part. The information provided is insufficient to repeat the experiments/analyses or to assess the quality of the work within the scope of reviewing this manuscript. For example, the rather complex ToF-SIMS analysis is used. Here, only the device type is named, followed by two confusing pieces of information.
“In addition, the three-dimensional structure of AlGa was characterized by time-of-flight secondary ion mass spectrometry (TOF-SIMS) (IONTOF M6), which was equipped with a 30 kV Bi+ analysis beam and 2000 eV Ar ion sputtering.”
Fact is that the device is equipped with a 30 kV liquid metal bismuth ion source. Several primary ion species are available to choose from. The same applies to the 2 kV Ar source. Here, several energies can be selected. The beam is then an Ar+ beam. Essential information is missing about the method itself in order to repeat the measurements. No statement was made about the ablation rate for metallic Ga mentioned in the caption to Figure S7. With so few measured data points, the depth profiling should be repeated even with a lower energy sputter beam. For data reporting in ToF-SIMS I can recommend the paper by Graham and Gamble (https://doi.org/10.1116/6.0002633). In section IV they state what is essential following the rules of good scientific practice. This statement applies to all other analytical techniques used.
8) The initials in the Author contribution section cannot be attributed to the authors. I have the feeling that this section has never been edited. Given the multitude of methods used, there are also doubts as to whether one person carried out all the experiments and analyses.

Version 1:

Reviewer comments:

Reviewer #1

(Remarks to the Author)
The authors have made substantial efforts to address my previous concerns by providing additional electrochemical data, nanoindentation results, and TOF-SIMS analysis. I appreciate the revisions and the expanded discussion. However, after carefully examining the responses and the newly provided data, several important issues remain insufficiently resolved. Some mechanistic interpretations are still not fully convincing, and certain conclusions appear to be overextended relative to the presented evidence.
1. While the authors argue that Ga is confined at grain boundaries and exists in low concentration, the core issue regarding its low melting point has not been fundamentally addressed. The resolution of EDS is extremely limited, which makes it impossible to rule out the occurrence of micro-scale liquid migration and unable to prove that Ga does not undergo localized rearrangement. The nanoindentation argument is not fully conclusive, as hardness variations can arise from multiple factors beyond Ga redistribution (e.g., surface corrosion, SEI coverage, residual stress). Therefore, the stability of Ga under electrochemical and thermal stress remains only indirectly supported.
2. The CV results clearly reveal detectable Na-Ga alloying peaks, confirming the formation of NaGa4, as acknowledged by the authors in their response to Comment 3. A weak alloying peak does not necessarily correspond to a small volume change. The peak intensity in CV primarily reflects electrochemical kinetics and current response, rather than directly quantifying the magnitude of structural expansion. Even if the extent of alloy formation is limited, the associated localized volume changes may still induce significant mechanical stress, particularly at grain boundaries.
3. The authors attribute the capacity increase to accelerated Al3+ dissolution, which supposedly facilitates Na+ utilization. If additional electrons originate from Al oxidation (Al → Al3+ + 3e-), this represents an irreversible parasitic reaction rather than reversible battery capacity, and this would contradict the claim of structural stability and raise concerns about long-term durability.
4. I am still confused about “The dissolved Al3+ in the electrolyte alters the local coordination environment through interactions with anions and solvent molecules perturbing the Na+ solvation equilibrium and promoting the dissociation of coordinated Na+, which subsequently migrates toward the cathode for charge compensation.” Why could this happen? This requires a clear electrochemical driving force. It is not evident how local perturbation of Na^+ solvation by Al^{3+} would independently generate a net Na^+ flux toward the cathode beyond that already imposed by the external current.

Reviewer #2

(Remarks to the Author)

The author has well addressed all my comments. I suggest to accept the contribution at the current status.

Reviewer #3

(Remarks to the Author)
I have looked at the revised manuscript and the response letter to all 4 reviewers. Overall, the authors have done a good job in revision. I am happy to recommend its acceptance of this excellent work for publication in Nat comm. The only minor comment I have now is that there are some minor errors in the references in year number, volume number, page number/article number, etc.
These can be corrected during the proof stage.

Reviewer #4

(Remarks to the Author)
The authors have satisfactorily addressed the raised concerns and revised the manuscript accordingly. However, two minor issues have come to my attention that should be addressed prior to publication:

In the abstract you write „The AlGa||Na3V2(PO4)3 pouch cell". Please give the full cell including the electrolyte. This makes it clearer for the reader. This also counts for the other mentioned cells.

In Supplementary Fig. 22 your write in the tables "Sputter phase (for cleaning)". Please correct to "sputter beam". I am wondering why you are writing "cleaning". It is standard depth profiling, and you want to show the 3D SEI composition. Or am I wrong? – Same for Fig. 26

Version 2:

Reviewer comments:

Reviewer #1

(Remarks to the Author)
The author has adequately addressed my comments and concerns. I have no further suggestions. Therefore, I recommend accepting the manuscript in its current form.

Open Access This Peer Review File is licensed under a Creative Commons Attribution 4.0 International License, which permits use, sharing, adaptation, distribution and reproduction in any medium or format, as long as you give appropriate credit to the original author(s) and the source, provide a link to the Creative Commons license, and indicate if changes were made.
In cases where reviewers are anonymous, credit should be given to 'Anonymous Referee' and the source.

The images or other third party material in this Peer Review File are included in the article's Creative Commons license, unless indicated otherwise in a credit line to the material. If material is not included in the article's Creative Commons license and your intended use is not permitted by statutory regulation or exceeds the permitted use, you will need to obtain permission directly from the copyright holder.
To view a copy of this license, visit https://creativecommons.org/licenses/by/4.0/

Itemized Responses/Revisions to the original manuscript

Responses to reviewer 1:

The manuscript reports a strategy for stabilizing sodium deposition through a hierarchically alloyed current collector. The authors utilize a grain-boundary gallium-rich polycrystalline aluminum current collector (AlGa) to trigger controlled dissolution of Al^{3+} and localized charge redistribution. The pouch cells are constructed by coupling AlGa with commercial $Na_3V_2(PO_4)_3$. However, I have several critical concerns over this work, which I believe preclude its further consideration for acceptance in Nature Communications.

Response:

Thank you very much for your comments on our work. In response to your concerns, we have provided additional clarification regarding the stability of Ga in AlGa and its interaction with Na. We have also included supplementary results on the AlGa after long-term cycling and corrected several imprecise descriptions in the manuscript. Furthermore, the composition and spatial distribution of the SEI formed on the AlGa surface have been analyzed and discussed in more detail. We also clarified the origin of the slight capacity increase observed at rate performance. We hope that these revisions and explanations adequately address your comments.

Comment 1:

Gallium metal has a melting point of 29.76 °C, rendering it highly susceptible to liquefaction. This tendency is further exacerbated when forming alloys, which can lower their melting point. Consequently, the question arises as to how the gallium layer can be assured of stable existence upon the Al current collector in this manuscript.

Response:

We sincerely thank the reviewer for this careful and insightful comment.

Although gallium (Ga) metal has a low melting point (29.76 °C) and its melting temperature can be further reduced upon alloying, the Ga in our AlGa current collector does not exist as a continuous metallic layer. Instead, Ga is initially confined at the grain boundaries of the Al matrix and partially incorporated into the Al lattice, forming an Al–Ga solid solution with a very low Ga fraction. Consequently, no large-scale liquefaction or migration of Ga occurs.

Importantly, the stability of Ga in the AlGa current collector is experimentally verified by cycling characterization. SEM images of the AlGa current collector after cycling in anode-free Na batteries, including extended cycling up to 860 cycles (Supplementary Fig. 20–21), show that although localized surface peeling can be observed due to partial Al^{3+} dissolution during long-term cycling, the overall surface morphology remains relatively flat. Furthermore, energy-dispersive X-ray spectroscopy (EDS) mapping reveals that the elemental distribution of Ga and Al remains largely unchanged after cycling, and the Ga/Al ratio shows no noticeable

degradation.

In addition, apart from a portion of Ga alloying with Na, a portion of Ga may still accumulate at the grain boundaries. As is well known, the aggregation of gallium at the aluminum grain boundaries significantly weakens the intergranular bonding force, thereby causing a significant reduction in hardness[1, 2]. Therefore, if the gallium still accumulates at the grain boundaries during the cycling process, the hardness is expected to be comparable to that of the original AlGa sample. Thus, we conducted nanoindentation measurements on the AlGa samples after the cycling treatment. To further rule out the possibility that the hardness reduction is caused by the enrichment of gallium at the grain boundaries and its dissolution in the aluminum lattice (solid solution), we prepared a control sample (labeled as H-AlGa) by annealing aluminum-gallium at 800°C for 5 hours to promote the complete diffusion of gallium into the aluminum lattice. Considering that the heat treatment itself may affect the hardness, we also tested pure aluminum foil (labeled as Al-800°C) after the same annealing treatment for comparison.

The results show that the hardness of both H-AlGa and Al-800°C is significantly higher than that of the prepared aluminum-gallium samples, indicating that the uniform dissolution of gallium in the aluminum lattice cannot explain this significant softening phenomenon. Notably, after 100 cycles, the hardness of the AlGa sample is still comparable to the original AlGa and is significantly lower than H-AlGa and Al-800°C.

These results collectively demonstrate that, despite the intrinsically low melting point of Ga, its grain boundary confined distribution and low overall content ensure a stable existence on the Al current collector, enabling long-term structural and electrochemical stability in anode-free configurations.

We have modified the content in revised manuscript (see page 13, lines 301-308.) The revised description is as follows:

"After 100 and 860 cycles (Supplementary Fig. 20–21), slight surface exfoliation is observed on the AlGa current collector due to Al dissolution, while the overall surface remains essentially flat. EDS mapping confirms uniform elemental distribution and negligible Na accumulation, indicating suppression of "dead Na" and overall structural stability during long-term cycling. Nanoindentation results (Supplementary Table 1) show that the hardness of AlGa after cycling is comparable to that before cycling, confirming the persistent accumulation of Ga at the grain boundaries."

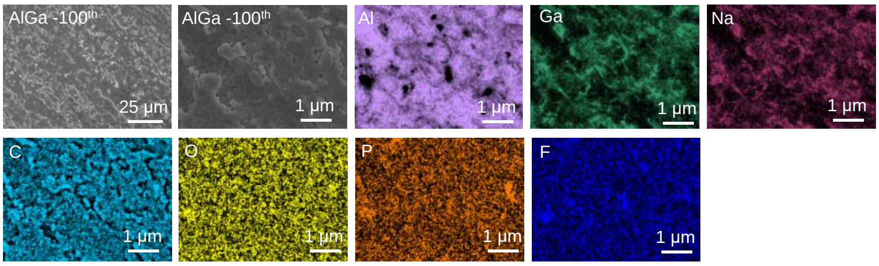

Element	Atom(%)
Al	62.12
Ga	2.64
Na	0.78
C	22.38
O	8.94
P	0.01
F	3.13
total	100

Supplementary Fig.20 SEM images of Na deposition AlGa(a) and Al(b) after 100 cycles at 1mA cm^{-2} for 1 mAh cm^{-2}

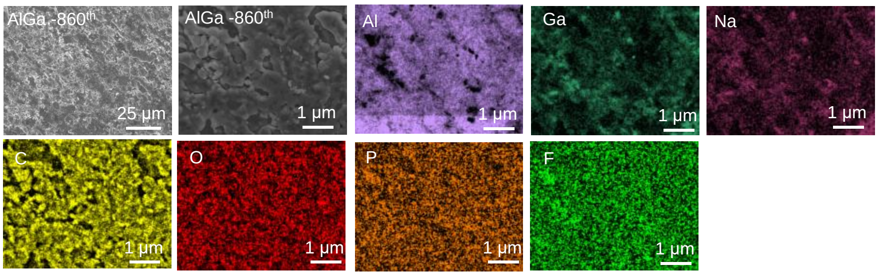

Element	Atom(%)
Al	64.80
Ga	1.85
Na	0.66
C	21.21
O	8.51
P	0.00
F	2.96
total	100

Supplementary Fig.21 SEM images of Na deposition AlGa(a) and Al(b) after 860 cycles at 1mA cm^{-2} for 1 mAh cm^{-2}

Supplementary Table 1 The hardness (H-IT) of Al, AlGa, H-AlGa, Al-800°C, AlGa after 100 cycled.

sample	H-IT (Gpa)	Average H-IT (Gpa)
Al	0.807	0.804
	0.783	
	0.821	
AlGa	0.280	0.314
	0.262	
	0.401	
H-AlGa	0.572	0.586
	0.566	
	0.621	
Al-800°C	0.543	0.521
	0.507	
	0.515	
AlGa after 100 cycled	0.373	0.311
	0.229	
	0.332	

The H-IT data were determined in accordance with ISO 14577.

Comment 2:

Gallium metal also forms alloys with sodium metal, causing volume expansion and internal stress that adversely affect the long-cycle stability of current collectors, particularly in anode-free configurations. However, the authors did not mention anything about this issue in the manuscript.

Response:

Thank you very much for your valuable comments.

Although Na–Ga alloying is typically accompanied by substantial volume expansion, the extent of alloying in our system is spatially confined. This limited alloying behavior originates from the initial distribution of Ga confined at the grain boundaries of the Al matrix. In this configuration, Ga is surrounded by the Al lattice and exists in a nanoscale form. Such a structural limitation effectively reduces the

alloying reaction of Na–Ga.

In Supplementary Fig. 14(a), we assembled half cells and performed cyclic voltammetry (CV) measurements at a scan rate of 0.1 mV s^{-1} within a voltage window of −0.05 to 1.0 V (vs. Na/Na^+). Under these conditions, no obvious Na–Ga alloying behavior was observed. This is mainly attributed to the dominant Na deposition/stripping process, whose large current response overwhelms the much weaker alloying signal, together with the limited sensitivity of the electrochemical workstation at extremely low currents.

To suppress excessive Na deposition, the lower cutoff potential was further narrowed to −0.005 V while maintaining the same scan rate (0.1 mV s^{-1}, Supplementary Fig. 14b–c). Under this condition, Na deposition is significantly reduced, and the weak Na–Ga alloying behavior becomes detectable. During the first discharge, no pronounced cathodic peak associated with Na–Ga alloying is observed, whereas a characteristic Al dissolution peak (~0.71 V) appears during the subsequent stripping process. In the second cycle, a weak cathodic peak at ~0.62 V corresponding to Na–Ga alloying and a shallow anodic peak at ~0.78 V related to Na–Ga dealloying (vs. Na/Na^+) are identified[3].

Consequently, instead of acting as a bulk alloying phase, grain-boundary Ga primarily modulates the surface energy and interfacial wettability of the current collector, thereby promoting uniform Na nucleation and deposition. As a result, limited deterioration in long-term cycling stability attributable to Na–Ga alloying is observed, even in anode-free configurations.

We have modified the content in revised manuscript (see page 11, lines 243-250.) The revised description is as follows:

"The CV (Supplementary Fig. 14) curve further confirmed that AlGa has a stronger response current density and a lower deposition overpotential. In addition, a weak cathodic peak at 0.62 V corresponding to Na–Ga alloying and anodic peaks at 0.71 and 0.78 V, which are related to Al dissolution and Na–Ga dealloying (vs. Na/Na^+), respectively, are identified. In this system, Na–Ga alloying is limited compared with Na deposition/stripping, owing to the initial confinement of Ga at the grain boundaries of the Al, which effectively minimizes the detrimental volume changes associated with alloying."

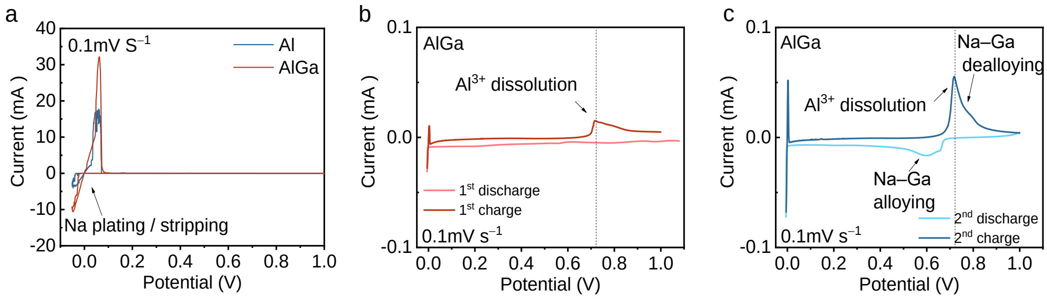

Supplementary Fig. 14 CV curves AlGa || Na and Al || Na cycling at 0.1 mV s^{-1}. The voltage scanning range is -0.05V – 1V(a) and -0.005V –1V(b-c).

Comment 3:

The author mentioned that “Further, the Ga penetration along the grain boundaries generates amorphous regions, which weaken the Al crystal structure and facilitate Al atoms gradual dissolution.” How can the stability of the collector be ensured over extended cycles when Al current collectors are subject to continuous corrosion? The manuscript only presents SEM images after 15 cycles. The morphology and phase distribution of the alloy current collector should be characterized after longer cycling (e.g., 1000 cycles).

Response:

We sincerely thank the reviewer for this careful and insightful comment.

The long-term stability of the AlGa current collector was experimentally verified through extended cycling tests. SEM images of the AlGa current collector retrieved from anode-free Na batteries after prolonged cycling, including 100 and 860 cycles (Supplementary Fig. 20–21), indicate that although localized surface peeling can be observed—likely associated with partial Al^{3+} dissolution during long-term operation—the overall surface morphology remains relatively flat and structurally intact.

From a phase perspective, the formation of a $NaGa_4$ phase is additionally identified. Energy-dispersive X-ray spectroscopy (EDS) reveals a strong spatial correlation between the Na signal and Ga-rich regions, indicating localized Na–Ga interactions. This suggests that after extended cycling, $NaGa_4$, which is the most commonly reported Na–Ga alloying phase[3], may form locally on the AlGa current collector. Notably, the Na signal on the cycled surface is extremely weak, suggesting negligible accumulation of residual sodium. This observation indicates that the formation of “dead Na” is effectively suppressed on the AlGa current collector during long-term cycling.

We have modified the content in revised manuscript (see page 11, lines 243-250.)

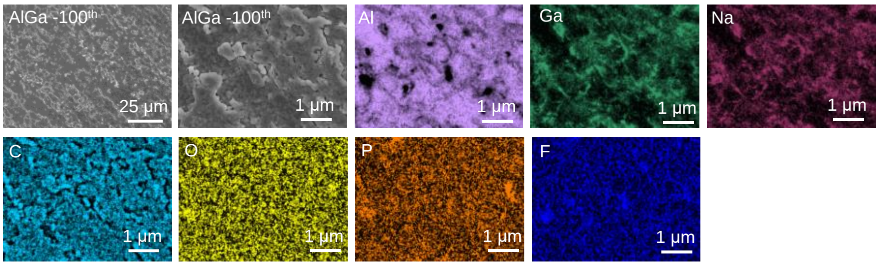

Element	Atom(%)
Al	62.12
Ga	2.64
Na	0.78
C	22.38
O	8.94
P	0.01
F	3.13
total	100

Supplementary Fig. 20 SEM images of Na deposition AlGa(a) and Al(b) after 100 cycles at 1mA cm^{-2} for 1 mAh cm^{-2}

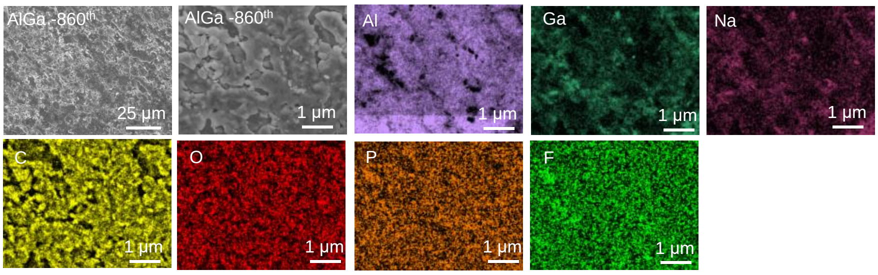

Element	Atom(%)
Al	64.80
Ga	1.85
Na	0.66
C	21.21
O	8.51
P	0.00
F	2.96
total	100

Supplementary Fig. 21 SEM images of Na deposition AlGa(a) and Al(b) after 860

cycles at 1mA cm^{-2} for 1 mAh cm^{-2}

Comment 4:

In Supplementary Fig. 17, the cross-sectional SEM images of Na deposition on the Al current collector also appear to be smooth and dense, which is inconsistent with the author's description (drastic inhomogeneous Na deposition). Please provide clearer planar and cross-sectional SEM images and explain why the uniform deposition thickness reaches 31μm at a current density of 1 mA cm^{-2}.

Response:

Thank you for your careful inspection and constructive comments. We have provided additional planar and cross-sectional SEM images at higher magnifications to better clarify the Na deposition behavior (Supplementary Fig. 18).

At low magnification, thicker Na deposits may appear locally smooth and dense in cross-sectional scanning electron microscope images. Therefore, we provide magnified planar and cross-sectional SEM images of Na deposition that clearly reveal distinct pits on the Al surface, consistent with highly uneven sodium deposition. In contrast, Na deposited on the AlGa current collector exhibits a much more uniform morphology. Both planar and cross-sectional views show a continuous and homogeneous Na layer without obvious pits or large thickness variations, indicating regulated nucleation and growth behavior.

It should be clarified that the term "drastic inhomogeneous Na deposition" refers to the macroscopic non-uniformity of Na deposition across the Al current collector. Specifically, Na deposition on bare Al tends to preferentially grow on pre-deposited Na nuclei, leading to non-uniform Na deposition process, where Na accumulates excessively in certain regions (up to ~31 μm) while other areas remain extremely thin (1 μm) or nearly bare.

We have modified the content in revised manuscript (see page 13, lines 292-297). The revised description is as follows:

"In contrast, Na deposition on bare Al tends to preferentially grow on pre-deposited Na nuclei, leading to non-uniform Na deposition process. As a result, Na accumulates excessively in certain regions (up to ~31 μm) while other areas remain extremely thin (1 μm) or nearly bare. These observations demonstrate that the tip effect induces drastic inhomogeneous Na deposition."

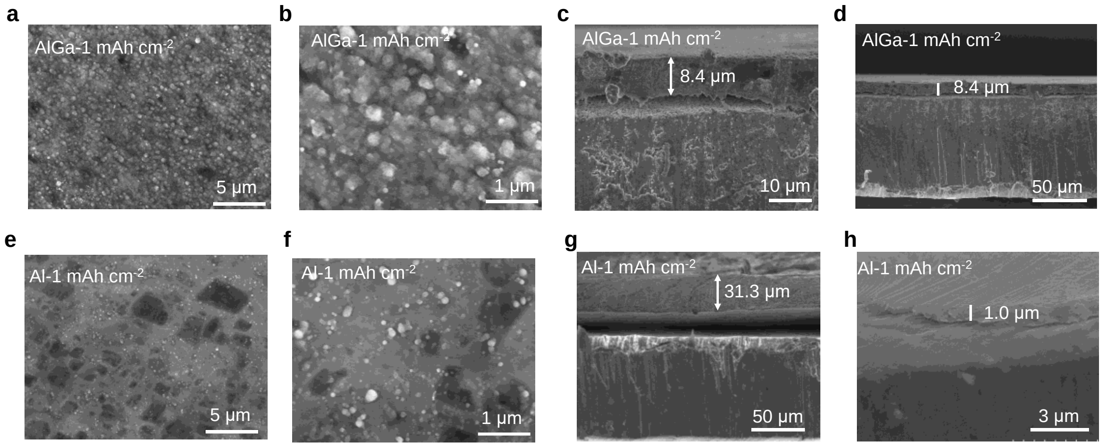

Supplementary Fig. 18 cross-sectional SEM images of Na deposition AlGa (a-d) and

Al(e-f) at 1mA cm^{-2} for 1 mAh cm^{-2}.

Comment 5:

The authors declare AlGa leading to a uniform SEI layer formation, but it does not provide a systematic analysis of its chemical composition and formation mechanism. Please supplement the test results and analyze the reasons for this result.

Response:

Time-of-flight secondary ion mass spectrometry (TOF-SIMS, Supplementary Fig. 22-23) was employed to characterize the composition and spatial distribution of the solid electrolyte interphase (SEI) formed on the AlGa and Al surface after cycling. Typical organic fragments, including $NaCHO^-$, CH_3ONa^-, and C_2HO^-, are commonly attributed to the decomposition of diglyme. In the AlGa sample, the intensities of these organic fragments decrease gradually and uniformly from the surface toward the interior, whereas in the Al sample they exhibit a much higher surface concentration followed by a sharp decline, indicating more severe ether decomposition reactions on the Al surface. Additionally, inorganic fragments such as Na_2O^-, NaF_2^-, and CF^-, which mainly originate from $NaPF_6$ decomposition, display a more homogeneous depth distribution in the AlGa sample, while a highly nonuniform distribution is observed for the Al sample. Moreover, a higher abundance of inorganic species, including $NaCO_2^-$ and $NaGa^+$, is detected in the AlGa sample, which is expected to help the SEI with enhanced mechanical robustness. Overall, the modified AlGa electrode exhibits a SEI with uniformly distributed organic and inorganic components in both the lateral and depth directions, whereas the SEI formed on the Al electrode is highly heterogeneous. We have added the content in revised manuscript (see page 14-15, lines 320-336).

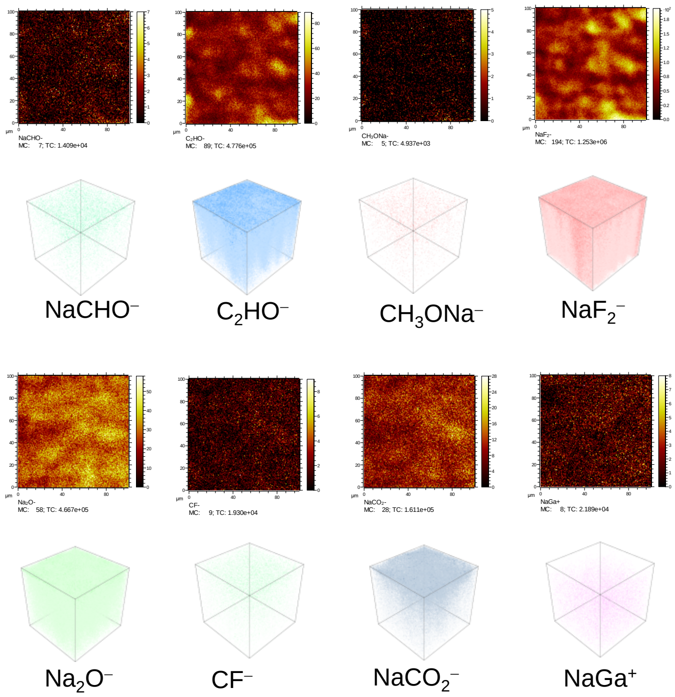

Instrument: IONTOF M6-Hybrid	
Acquisition phase	
Ion species	Bi3 (positive mode)
Energy	30 keV
Ion current	0.517 pA
Raster size	100 µm x 100 µm
pixel dimensions	128 x 128
Mass range	0 - 270 u
Mode	All-Purpose (Hybrid)
Analysis time	600 s (spectra)
Sputter phase (for cleaning)	
Ion species	Ar
Energy/Current	500 eV / 65.020 nA
Raster size	300 µm x 300 µm
Sputtering time	600 sec

Instrument: IONTOF M6-Hybrid	
Acquisition phase	
Ion species	Bi3 (negative mode)
Energy	30 keV
Ion current	0.518 pA
Raster size	100 µm x 100 µm
pixel dimensions	128 x 128
Mass range	0 - 270 u
Mode	All-Purpose (Hybrid)
Analysis time	600 s (spectra)
Sputter phase (for cleaning)	
Ion species	Ar
Energy/Current	500 eV / 64.560 nA
Raster size	300 µm x 300 µm
Sputtering time	600 sec

Supplementary Fig. 22 TOF-SIMS 3D reconstruction of the sputtered volume of several secondary ion fragments on the cycled AlGa surface.

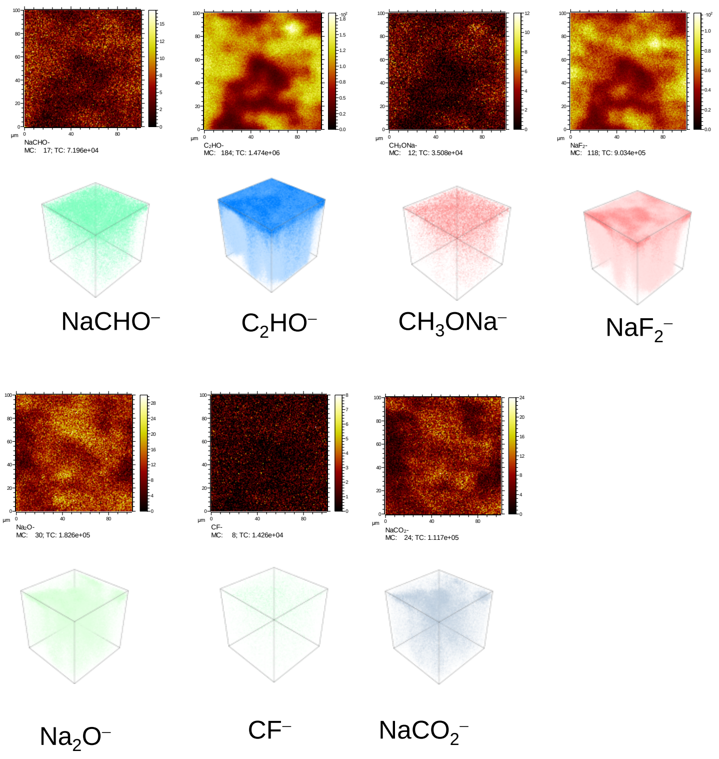

Instrument: IONTOF M6-Hybrid	
Acquisition phase	
Ion species	Bi3 (positive mode)
Energy	30 keV
Ion current	0.511 pA
Raster size	100 µm x 100 µm
pixel dimensions	128 x 128
Mass range	0 - 270 u
Mode	All-Purpose (Hybrid)
Analysis time	600 s (spectra)
Sputter phase (for cleaning)	
Ion species	Ar
Energy/Current	500 eV / 65.300 nA
Raster size	300 µm x 300 µm
Sputtering time	600 sec

Supplementary Fig. 23 TOF-SIMS 3D reconstruction of the sputtered volume of several secondary ion fragments on the cycled Al surface.

Comment 6:

The capacity of AlGa||NVP cell in Figure 5c increases with rising current density (after 8C). What is the reason for this phenomenon? Is it because dissolved Al^{3+} ions participate in the battery reaction? If not, please explain the reason of the sudden increase in capacity.

Response:

Thank you for this insightful comment. In general, increasing the current density

leads to a decrease in capacity due to kinetic limitations, which is also observed in this system at current densities below 8C. However, when the current density exceeds 8C, the dissolution of Al^{3+} from the AlGa current collector is significantly accelerated, resulting in an increased concentration of Al^{3+} species in the electrolyte. This process facilitates the migration and utilization of Na^{+} ions from the electrolyte toward the positive electrode, thereby providing additional charge compensation and giving rise to an apparent capacity increase at high current densities.

To verify this interpretation, AlGa||NVP full cells were assembled and first activated for one cycle at 0.1 C, followed by direct cycling at 4 C, 8 C, and 12 C for 20 cycles. After cycling, the PP separators from cells operated at different current densities were retrieved and immersed in an Al-specific reagent solution (Supplementary Fig. 29). The dissolved Al^{3+} content was analyzed by UV–Vis spectroscopy. As shown by the results, the amount of dissolved Al^{3+} increases with increasing current density (12 C > 8 C > 4 C). We have added the clarifications in revised manuscript (see page 19, lines 416-422).

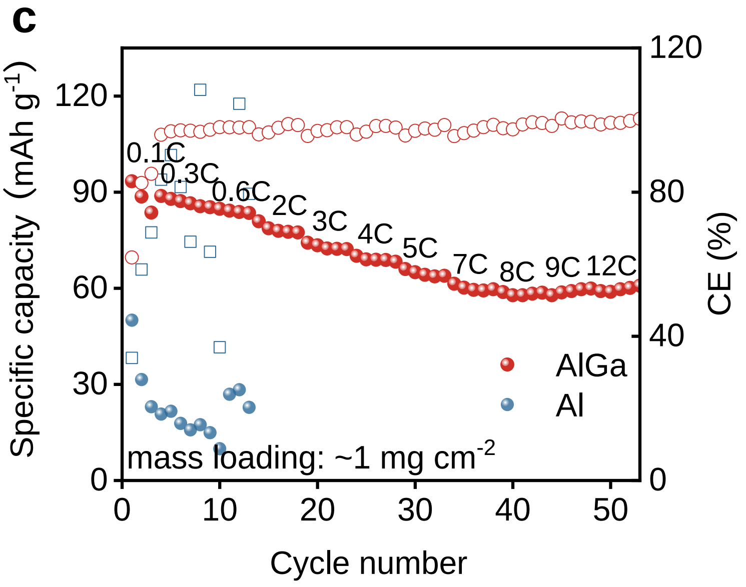

Fig 5 (c) Rate performance of AlGa||NVP and Al||NVP anode-free cells.

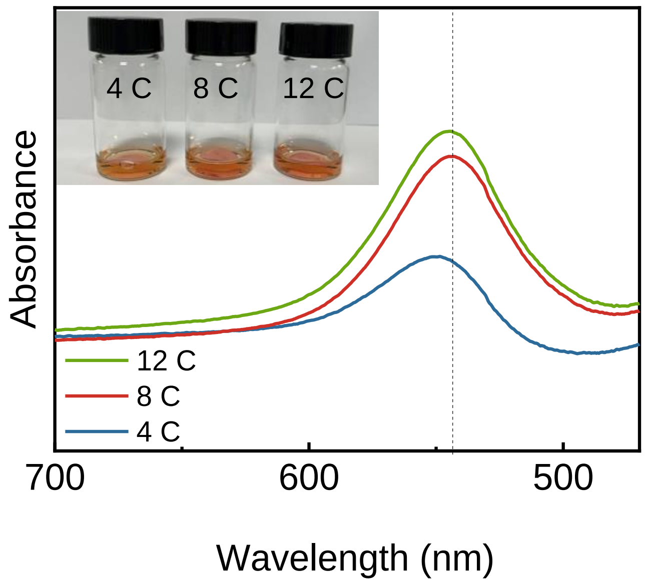

Supplementary Fig. 29 UV-vis spectra of electrolyte-infused PP separators from AlGa||NVP at different current density (4 C, 8 C,12 C) for 20 cycles reacted with Al reagent solution. Inset is Optical images.

Reference

1. Rajagopalan M, Bhatia MA, Tschopp MA, Srolovitz DJ, Solanki KN. Atomic-scale analysis of liquid-gallium embrittlement of aluminum grain boundaries. Acta Materialia 73, 312-325 (2014).
2. Ludwig W, Bellet D. Penetration of liquid gallium into the grain boundaries of aluminium: a synchrotron radiation microtomographic investigation. Materials Science and Engineering: A 281, 198-203 (2000).
3. Lv X, *et al.* Sodium–Gallium Alloy Layer for Fast and rReversible Sodium Deposition. *SusMat* **2**, 699-707 (2022).

Responses to reviewer 2:

This manuscript addresses the critical issue of rapid active Na depletion in anode free sodium metal batteries by presenting a cutting-edge strategy, which holds great relevance and timeliness for the advancement of post-lithium battery technologies. The AlGa collector design demonstrates innovation and feasibility for mass production, and the superior performance of both Na||AlGa and AlGa||NVP cells is commendable. Additionally, the proposed mechanism of charge compensation offers useful insights for collector engineering, making the work likely to interest battery researchers. To further enhance the manuscript's quality, a few issues need clarification and improvement. Therefore, I recommend accepting this manuscript once minor revisions are completed.

Response:

We thank the reviewer for providing us with the opportunity to revise this manuscript and greatly appreciate the reviewer's constructive and critical comments. In response, we have addressed the questions regarding the continuous release of aluminum species in AlGa, as well as the origin of the C and O signals correlated with Ga. We have also further clarified the Coulombic efficiency (CE) behavior and the fluctuations observed around 3.4 V. In addition, experimental details concerning the transfer of samples to the testing procedures have been supplemented, and several typographical and writing errors in the manuscript have been corrected. We sincerely thank the reviewer for these valuable comments and hope that the revisions and explanations provided adequately address the concerns raised.

Comment 1:

The authors verified the dissolution of Al^{3+} from AlGa. I wonder if Al^{3+} in AlGa will be continuously released during the battery cycling process.

Response:

Thanks for your valuable comments. PP separators after cycling with different numbers in AlGa||Na and Al||Na cells were removed and immersed in Al reagent solution (Figure 1d). Remarkably, even minimal electrolyte infiltration (μL scale) into individual PP pieces resulted in significant Al^{3+} leaching. Moreover, the color changes to redder hue as the number of cycles increases, proving that the gradual dissolution of Al^{3+} along with the cycles, but compared to Al||Na, there are still no appearance about the Al^{3+} dissolution even after 200 cycles. The UV-Vis results are consistent with the above conclusions (Supplementary Fig. 3).

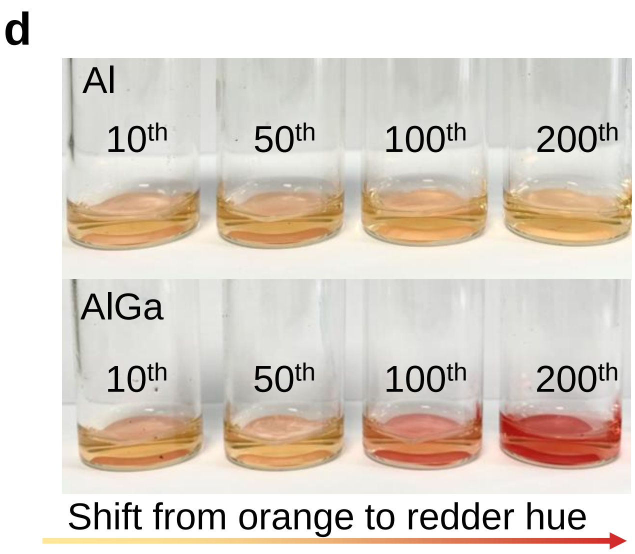

Fig.1 (d) The electrolyte on PP separators after cycling in Al||Na and AlGa||Na cells, reacted with Al reagent solution for optical visualization.

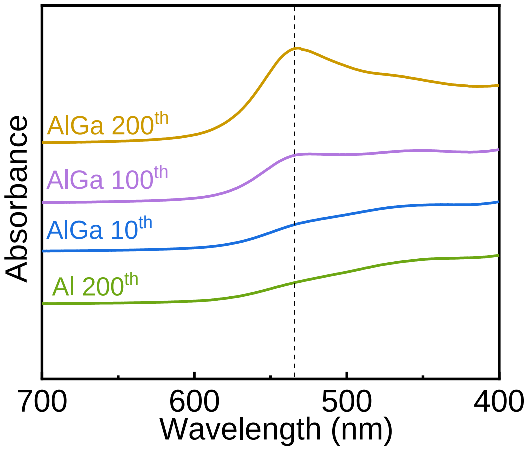

Supplementary Fig. 3 UV-Vis spectra of electrolyte-infused PP separators (Al||Na and AlGa||Na, different cycles) after reaction with Al reagent

Comment 2:

In Fig.2c, the distribution of C and O elements appears to correspond to that of Ga, what is their origin?

Response:

Thanks for your valuable comments. Ga have been reported to be able to adsorb oxygen- and carbon-containing substances from the surrounding environment[1, 2, 3], including carbon dioxide and organic molecules. Therefore, although our preparation process was completed in a glove box, the presence of organic solvents in the glove box inevitably causes Ga to adsorb on C/O, resulting in the synchronous observation of Ga, C, and O in the SEM. We have added the clarifications in revised manuscript (see page 7, lines 160-166).

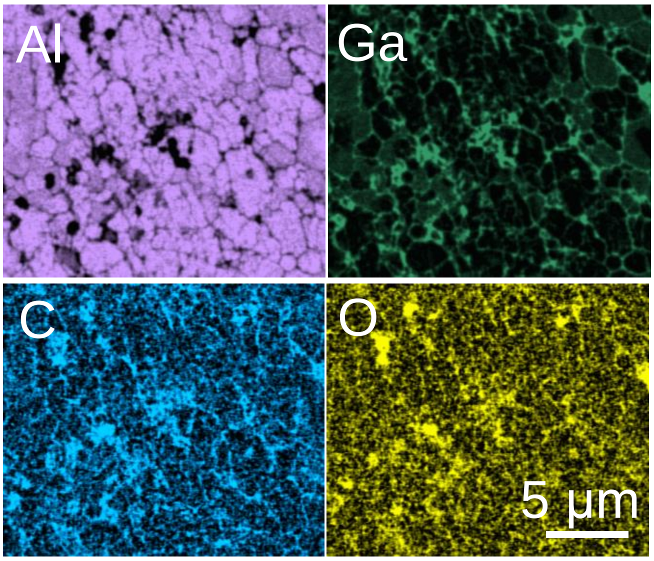

Fig. 2 (c) EDS mappings of AlGa.

Comment 3:

In Fig.5a, what are the reasons for the slight fluctuation in the Coulombic efficiency of the battery?

Response:

Thank you for your valuable comment.

In an anode-free sodium battery, Na^+ ions form a transient Na metal anode on the current collector (Al) during each charging process, while the deposited Na is expected to be stripped as completely as possible during discharge. Therefore, the electrical contact at the Al-Na-SEI three-phase interface directly determines which portion of the deposited Na can participate in electron transport and undergo reversible stripping. As a result, the stability of the electrical contact plays a critical role, and any contact instability is directly reflected as fluctuations in the Coulombic efficiency (CE).

During cycling, non-uniform Na deposition may occur, where Na accumulates excessively in certain regions while other areas remain extremely thin or nearly bare. Upon stripping, such inhomogeneous Na deposits can lead to the formation of voids, which disrupt the original Na and Al contact points. This disruption may induce the formation of newly reconstructed, electronically insulating SEI layers at the interface, causing part of the deposited Na to remain physically present but electrochemically inaccessible. Meanwhile, repeated volume changes during Na plating and stripping can trigger continuous SEI rupture and reconstruction, which may intermittently reopen electronic pathways. As a result, a portion of previously isolated Na can be released in subsequent cycles, showing as a recovery in CE. For the AlGa current collector, we propose that Ga first promotes homogeneous Na nucleation, leading to a more uniform and dense Na deposition. More importantly, Ga improves the interfacial electrical contact. Ga provides enhanced interfacial adhesion and compliance, thereby stabilizing the electrical contact between the deposited Na and the current collector. Consequently,

interfacial delamination and electrical disconnection are suppressed, resulting in a more stable Na stripping/plating process and reduced CE fluctuation.

It should be acknowledged that, although the interfacial stability and Coulombic Efficiency are significantly improved, the performance has not yet fully approached that of conventional Na metal batteries. Nevertheless, the present results demonstrate a substantial mitigation of interfacial instability inherent to anode-free configurations and provide a promising pathway toward further optimization.

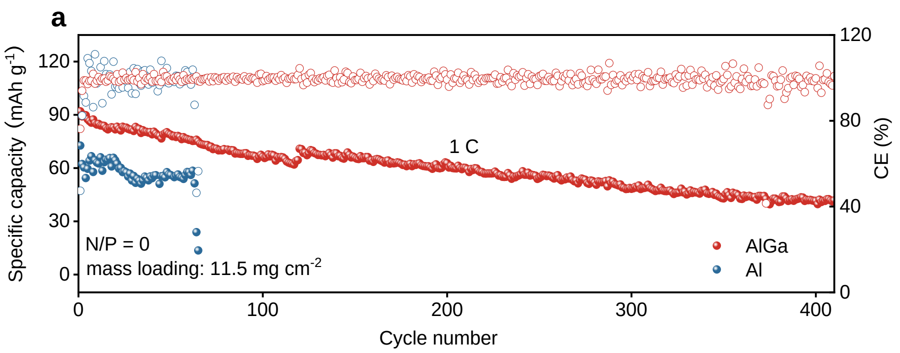

Fig. 5 (a) The cyclic stability of AlGa||NVP and Al||NVP anode-free cells at 1 C, 1 C=117mAh g^{-1}.

Comment 4:

In Fig.5b, there are fluctuations in the charge-discharge curve around 3.4 V, please explain the reason.

Response:

Thank you for your valuable comment.

As shown in Fig. R1, for the AlGa sample, voltage fluctuations primarily occur during the initial deposition stage near 3.4 V, corresponding to the formation of the solid electrolyte interphase (SEI). This phenomenon disappears in the second charging cycle. For the Al sample, unstable electrical contact persists throughout cycling. In addition to the initial fluctuations caused by SEI formation, significant voltage variations are still observed during the later stages of stripping. During cycling, uneven sodium deposition may occur, leading to localized over-accumulation of sodium in certain regions, while other areas remain thinly covered or nearly bare. For the stripping process, such heterogeneous deposition can result in the formation of voids, which disrupt the original Na and Al contact points. This disruption may induce the formation of a new, electronically insulating SEI layer at the interface. Consequently, even though some sodium remains physically deposited, it becomes electrochemically inaccessible, leading to persistent voltage fluctuations during stripping.

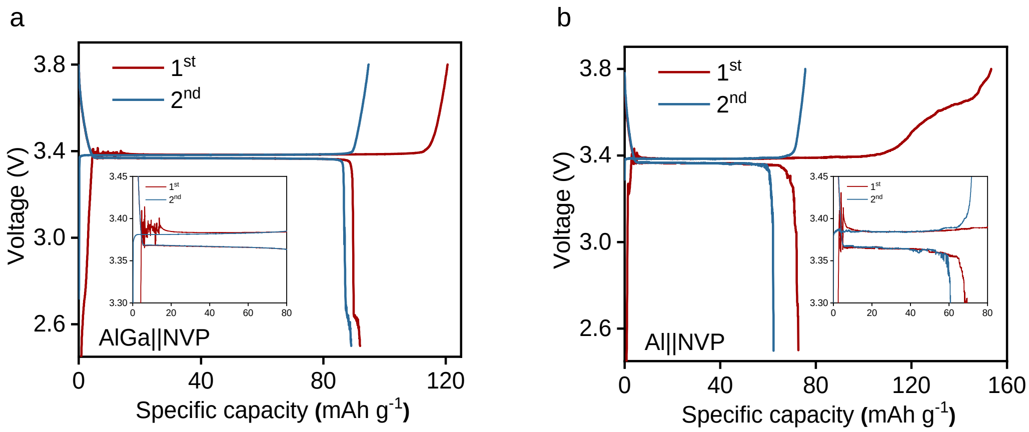

Fig. R1 The voltage-capacity curves of the AlGa || NVP(a) and Al || NVP(b) at first cycle.

Comment 5:

Please specify the sample preparation conditions and the specific transfer conditions in the Experimental Section.

Response:

Thank you for your valuable comment. In the experimental section, we have specified the sample preparation conditions and the specific transfer conditions. We have added the content in revised manuscript (see page 22, lines 493-496).

"All sample preparation and handling were conducted in an argon-filled glovebox (H2O < 0.1 ppm, O2< 0.1 ppm) unless otherwise stated. After cycling, electrodes were rinsed with diglyme, vacuum-dried at 25 °C, and transferred in sealed holders to prevent contamination."

Comment 6:

There are numerous formatting issues in the manuscript, the authors are requested to carefully check and revise them. For example, "2wt%" should be "2 wt%", "FigureS8" should be "Supplementary Fig. 8".

Response:

We sincerely thank you for the careful and rigorous comments. The mentioned examples have been corrected accordingly. In addition, all other similar formatting issues throughout the manuscript have been carefully checked and revised.

"NVP powder (92 wt%, NVP- polycrystalline (particle size 20 μm): NVP-single crystal (1–2 μm) = 4 : 1), CNT (1 wt%), graphene (2 wt%) were mixed and hand-milled for 30 min in marmalade mortar. "

"Further illustrating why Ga only needs to accumulate mostly at grain boundaries, we prepared samples homogeneous Al – Ga solid solutions (Supplementary Fig. 8, H-AlGa) and with surfaces full of Ga (Supplementary Fig. 9, S-AlGa)."

Reference

1. Ryu G, Park I, Kim H. Liquid Metal Micro- and Nanodroplets: Characteristics, Fabrication Techniques, and Applications. ACS omega 8, 15819-15830 (2023).

2. Wang X, et al. Reconfigurable and regenerable liquid metal surface oxide for continuous and quantifiable adsorption of biological dye. Applied Materials Today 26, 101265 (2022).

3. Ahmadian A, et al. Aluminum depletion induced by co-segregation of carbon and boron in a bcc-iron grain boundary. Nature Communications 12, 6008 (2021).

Responses to reviewer 3:

This work designs a current collector by coating Ga onto Al foil (AlGa) for anode-free Na batteries. The penetration of Ga into the grain boundaries of the Al foil induces the controlled dissolution of Al^{3+}, thereby altering the local electrolyte charge distribution and triggering spontaneous Na+ migration from the bulk electrolyte to the cathode. As a result, the AlGa//Na cell demonstrates stable cycling performance for 2500 cycles at 1 mA cm^{-2} and 1 mAh cm^{-2}. When paired with a $Na_3V_2(PO_4)_3$ cathode, the assembled anode-free cell realizes a high energy density exceeding 201.5 Wh kg^{-1}. Overall, this work presents some valuable and insightful research findings. The experiments are well conducted in general, and the results are carefully analyzed and generally support the conclusions. Therefore, I would like to recommend the acceptance of this work after some revisions noted below.

Response:

Thank you for reviewing our manuscript and for highlighting the remaining issues. In response to these comments, we have carefully examined the enrichment behavior of Ga at grain boundaries after cycling and investigated the influence of Ga on the mechanical properties and electrical conductivity of Al. And we have added cyclic voltammetry (CV) curves of AlGa||Na and Al||Na cells, and fitted the SEI resistance (R_{SEI}) and charge-transfer resistance (R_{ct}) based on the Nyquist plots of AlGa||Na and Al||Na. These analyses provide further insight into the interfacial kinetics and impedance evolution. In addition, we clarify how the AlGa current collector regulates the morphology of the Na plating layer during cycling. Moreover, cross-sectional SEM images of the NVP cathode have been provided to illustrate its thickness and structural integrity. We also include SEM images of the AlGa current collector after cycling in an anode-free sodium battery configuration to demonstrate its stability. Finally, the formatting and presentation issues in the manuscript have been carefully revised.

Comment 1:

The authors mention that enriching Ga at grain boundaries enhances battery performance. Can Ga at the grain boundaries maintain its stability during long-term cycling? Please clarify.

Response:

We sincerely thank you for this critical and constructive suggestion.

We appreciate your concern regarding the possibility that Ga may migrate from grain boundaries into the Al lattice or undergo Na–Ga alloying during cycling, potentially depleting Ga at grain boundaries and weakening its long-term ability to induce Al dissolution. However, since Ga is predominantly enriched at Al grain boundaries rather than uniformly distributed in the bulk lattice, the Na–Ga alloying reaction is spatially confined by the grain boundary regions. As a result, even though part of the Ga may participate in alloying, a significant fraction of Ga remains segregated at grain boundaries, continuously contributing to Al dissolution and

interfacial regulation.

Direct observation of Ga at grain boundaries after long-term cycling is experimentally challenging. Grain boundaries are only a few nanometers wide and typically require TEM for visualization. Nevertheless, given that the Al grain size is on the order of several micrometers, it is extremely difficult during TEM sample preparation to consistently obtain lamellae that simultaneously contain multiple grains and their connecting grain boundaries. In most cases, the prepared samples predominantly represent grain interiors rather than grain boundary regions.

To overcome this limitation, we employed an indirect but reliable mechanical characterization strategy. It is well established that Ga segregation at Al grain boundaries significantly weakens intergranular cohesion, resulting in a pronounced reduction in hardness[1,2]. Therefore, if Ga remains enriched at the grain boundaries after cycling, the hardness is expected to remain comparable to that of the pristine AlGa sample. Accordingly, nanoindentation measurements were performed on the cycled AlGa samples. To further exclude the possibility that the reduced hardness originates from Ga dissolved in the Al lattice (solid-solution), we prepared a control sample by annealing AlGa at 800 °C for 5 h to promote complete diffusion of Ga into the Al lattice (denoted as H-AlGa). Considering that thermal treatment itself may influence hardness, pure Al foil subjected to the same annealing process (Al-800 °C) was also tested for comparison.

The results show that both H-AlGa and Al-800 °C exhibit significantly higher hardness than the as-prepared AlGa sample, indicating that Ga uniformly dissolved in the Al lattice does not account for the pronounced softening. Notably, the hardness of the AlGa sample after 100 cycles remains comparable to that of the pristine AlGa and is still markedly lower than those of H-AlGa and Al-800 °C. This observation provides strong indirect evidence that Ga remains enriched at the grain boundaries even after extended cycling.

Therefore, although direct nanoscale visualization of grain-boundary Ga after cycling is challenging, the nanoindentation results convincingly demonstrate the long-term stability of Ga at grain boundaries, supporting its sustained role in regulating Al dissolution and enhancing battery performance.

We have modified the content in revised manuscript (see page 13, lines 305-308.) The revised description is as follows:

"Nanoindentation results (Supplementary Table 1) show that the hardness of AlGa after cycling is comparable to that before cycling, confirming the persistent accumulation of Ga at the grain boundaries."

Supplementary Table 1 The hardness (H-IT) of Al, AlGa, H-AlGa, Al-800°C, AlGa after 100 cycled.

sample	H-IT (Gpa)	Average H-IT (Gpa)
Al	0.807	0.804
	0.783	
	0.821	
AlGa	0.280	0.314

	0.262	
	0.401	
H-AlGa	0.572	0.586
	0.566	
	0.621	
Al-800℃	0.543	0.521
	0.507	
	0.515	
AlGa after 100 cycled	0.373	0.311
	0.229	
	0.332	

The H-IT data were determined in accordance with ISO 14577.

Comment 2:

How does the introduction of Ga affect the intrinsic properties of the Al current collector, such as its mechanical property and electrical conductivity?

Response:

We sincerely thank you for this critical and constructive suggestion.

The mechanical and electrical properties of AlGa and pristine Al were evaluated by nanoindentation and four-probe measurements, respectively. The hardness (H-IT) were determined in accordance with ISO 14577, while the electrical conductivity represents the electronic conductivity of the substrates. Owing to the presence of Ga at the grain boundaries, a reduction in modulus, hardness, and electrical conductivity is observed for AlGa. Nevertheless, despite the decrease in hardness and conductivity compared with pristine Al, the resulting values (0.31 GPa in hardness and 2.12×10^7 S m^{-1} in conductivity) remain fully sufficient for current-collector applications. We have added the content in revised manuscript (see page 7, lines 161-166; page 22-23, line 516-521).

Supplementary Table 1 The hardness (H-IT) of Al, and AlGa.

sample	H-IT (Gpa)	Average H-IT (Gpa)
Al	0.807	0.804
	0.783	
	0.821	
AlGa	0.280	0.314
	0.262	
	0.401	

Supplementary Table 2 The conductivity of Al and AlGa.

sample	Conductivity ($S\cdot m^{-1}$)	Average conductivity ($S\cdot m^{-1}$)
Al	2.55×10^7	2.64×10^7
	2.66×10^7	

	2.71×10^{7}	
AlGa	2.13×10^{7}	2.12×10^{7}
	2.10×10^{7}	
	2.14×10^{7}	

Comment 3:

Figure 3h illustrates improved Na^{+} transport kinetics at the AlGa interface. To further substantiate this conclusion, the CV curves of AlGa//Na and Al//Na cells should be provided.

Response:

Thank you for your valuable comment.

In Supplementary Fig. 14(a), we assembled half cells and performed cyclic voltammetry (CV) measurements at a scan rate of 0.1 mV s^{-1} within a voltage window of −0.05 to 1.0 V (vs. Na/Na^{+}). The CV curve further confirmed that AlGa has a stronger response current density and a lower deposition overpotential. Under these conditions, no obvious Na–Ga alloying behavior was observed. This is mainly attributed to the dominant Na deposition/stripping process, whose large current response overwhelms the much weaker alloying signal, together with the limited sensitivity of the electrochemical workstation at extremely low currents.

To suppress excessive Na deposition, the lower cutoff potential was further narrowed to −0.005 V while maintaining the same scan rate (0.1 mV s^{-1}, Supplementary Fig. 14b–c). Under this condition, Na deposition is significantly reduced, and the weak Na–Ga alloying behavior becomes detectable. During the first discharge, no pronounced cathodic peak associated with Na–Ga alloying is observed, whereas a characteristic Al dissolution peak (~0.71 V) appears during the subsequent stripping process. In the second cycle, a weak cathodic peak at ~0.62 V corresponding to Na–Ga alloying and a shallow anodic peak at ~0.78 V related to Na–Ga dealloying (vs. Na/Na^{+}) are identified[3]. We have added the content in revised manuscript (see page 10, lines 243-250).

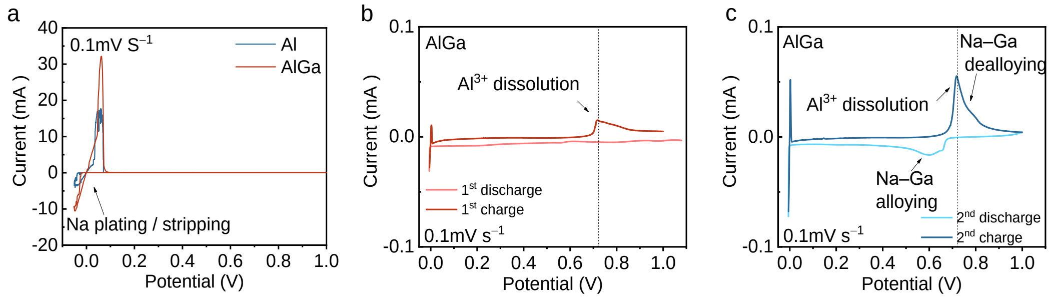

Supplementary Fig. 14 CV curves AlGa || Na and Al || Na cycling at 0.1 mV s^{-1}. The voltage scanning range is -0.05V – 1V(a) and -0.005V –1V(b-c).

Comment 4:

Figure S15 shows the Nyquist plots of AlGa/Na and Al/Na symmetric batteries at different temperatures. Please provide the equivalent circuit diagram to investigate the

evolution of RSEI and Rct.

Response:

Thank you for your valuable comment. Due to the complex interfacial processes involved, the impedance spectra could not be satisfactorily fitted using conventional equivalent circuit models. Therefore, the distribution of relaxation times (DRT) method was employed to deconvolute the impedance response by transforming the frequency-domain data into the time domain[4, 5]. By integrating the DRT peaks over specific relaxation time ranges, the resistance contributions associated with the SEI layer (R_{SEI}, τ : 10^{-6}–10^{-4} s) and charge transfer processes (R_{ct}, τ : 10^{-3}–10^{-1} s) were quantitatively extracted. Both R_{SEI} and R_{ct} decrease monotonically with increasing temperature, which is consistent with thermally activated ion transport and charge transfer processes following Arrhenius-type behavior (Supplementary Fig. 16, Supplementary Table 4).

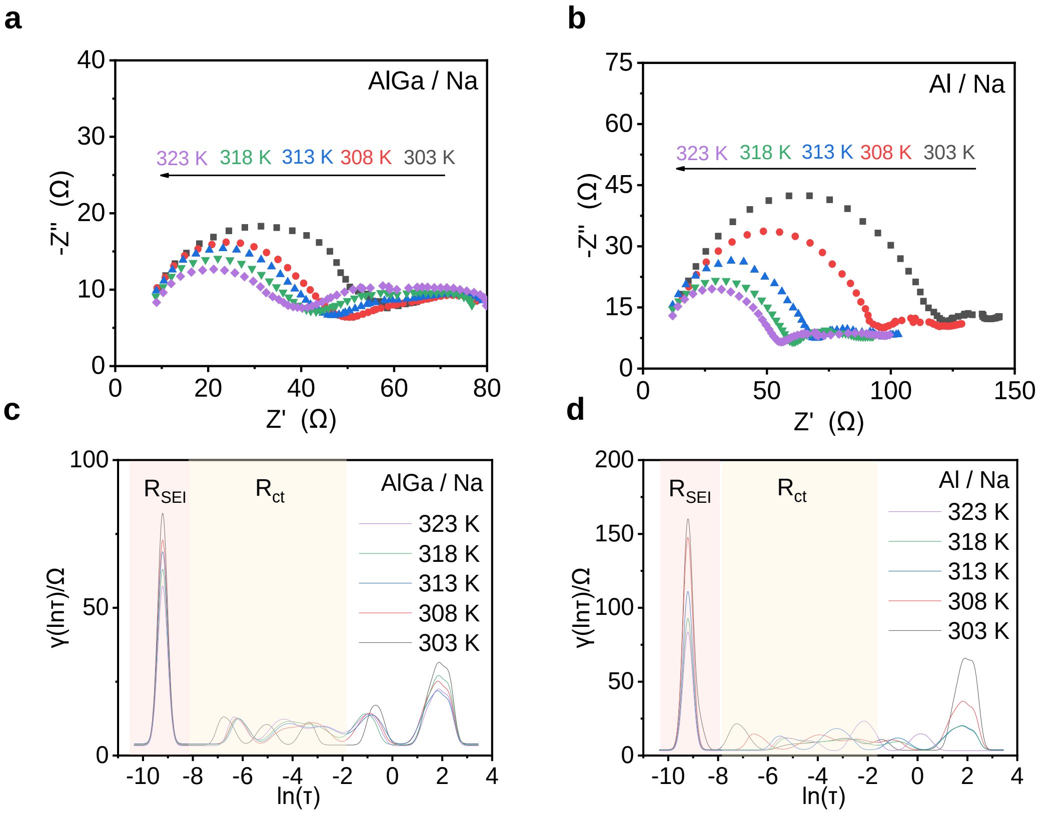

Supplementary Fig. 16 The nyquist plots and the corresponding DRT analyses of AlGa/Na (a, c) and Al/Na (b, d) based symmetric batteries at different temperatures (plating Na 1 mA cm^{-2} / 1 mAh cm^{-2}).

Supplementary Table 4 The R_{SEI} and R_{ct} results obtained by fitting based on the DRT results

AlGa		Al	
R_{SEI} (Ω)	R_{ct} (Ω)	R_{SEI} (Ω)	R_{ct} (Ω)
38.51	29.21	85.24	39.71
33.83	25.21	70.49	33.00
31.92	23.04	52.60	26.23

29.13	21.11	43.71	25.83
26.49	19.05	39.46	24.29

Comment 5:

Figure S16 shows that the AlGa current collector is covered by Na metal at an areal capacity of 0.1 mAh cm^{-2}. Please discuss in depth how the AlGa current collector continues to influence Na plating morphology under these conditions.

Response:

We appreciate your insightful comments. We discussed how the AlGa collector continues to influence the morphology of the Na coating and provides higher-magnification SEM images of Na deposition under these conditions. We have added the content in revised manuscript (see page 12-13, lines 279-288).

"The deposited Na exhibits a tighter interface with AlGa. In contrast, Na grows sparsely on pristine Al, with a clearly defined Na and Al boundary at a low areal capacity of 0.1 mAh cm^{-2} (Supplementary Fig. 17), which indicates inhomogeneous Na deposition and poor interfacial adhesion on the Al substrate. High magnification SEM images further reveal that the AlGa substrate maintains a dense and uniform Na deposition morphology even at high magnification, whereas the pristine Al substrate shows pronounced height variations and localized pit-like features. This behavior can be attributed to Ga enrichment at the AlGa grain boundaries, which provides effective nucleation sites and reduces the initial nucleation size of Na. As a result, stable electrical contact between Na and AlGa is maintained, and a more uniform Na deposition interface is achieved."

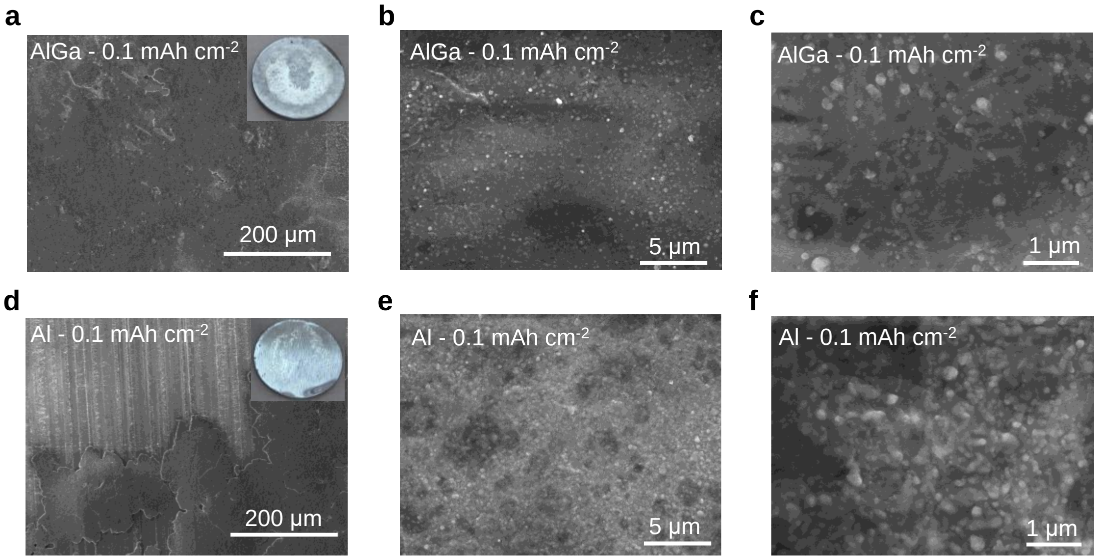

Supplementary Fig. 17 SEM images of Na deposition AlGa (a-c) and Al (b-d) at 1 mA cm^{-2} for 0.1 mAh cm^{-2}

Comment 6:

The anode-free AlGa||NVP cell with high mass loadings of the NVP cathode (~11.4 mg

cm-2 and ~47.40 mg cm-2) demonstrates stable cycling performance. To better correlate performance with electrode structure, it is recommended to include cross-sectional SEM images of the NVP cathode, illustrating its thickness and morphology.

Response:

Thank you for your valuable comments. SEM testing of the cycled NVP (11.40 mg cm^{-2}) reveals that the NVP matched with AlGa retained a relatively intact interfacial morphology with well-defined particles (Supplementary Fig. 24). And electrode thickness approaches that of fresh NVP (103 μm vs 106 μm). However, the thickness of the NVP cycled with Al decreased significantly (86.8 μm), indicating substantial collapse of the NVP structure. High magnification SEM images reveal that the surface particles of the NVP sample matched with AlGa exhibit intact boundaries. In contrast, the surface morphology of NVP cycled with Al appears relatively sticky and indistinct, confirming the presence of a thicker CEI layer. In Supplementary Fig. 25, even at high cathode loading (47.40 mg cm^{-2}), the thickness and particle integrity of NVP based cathode sheets remain excellent after cycling. We have added the content in revised manuscript (see page 16, lines 357-365).

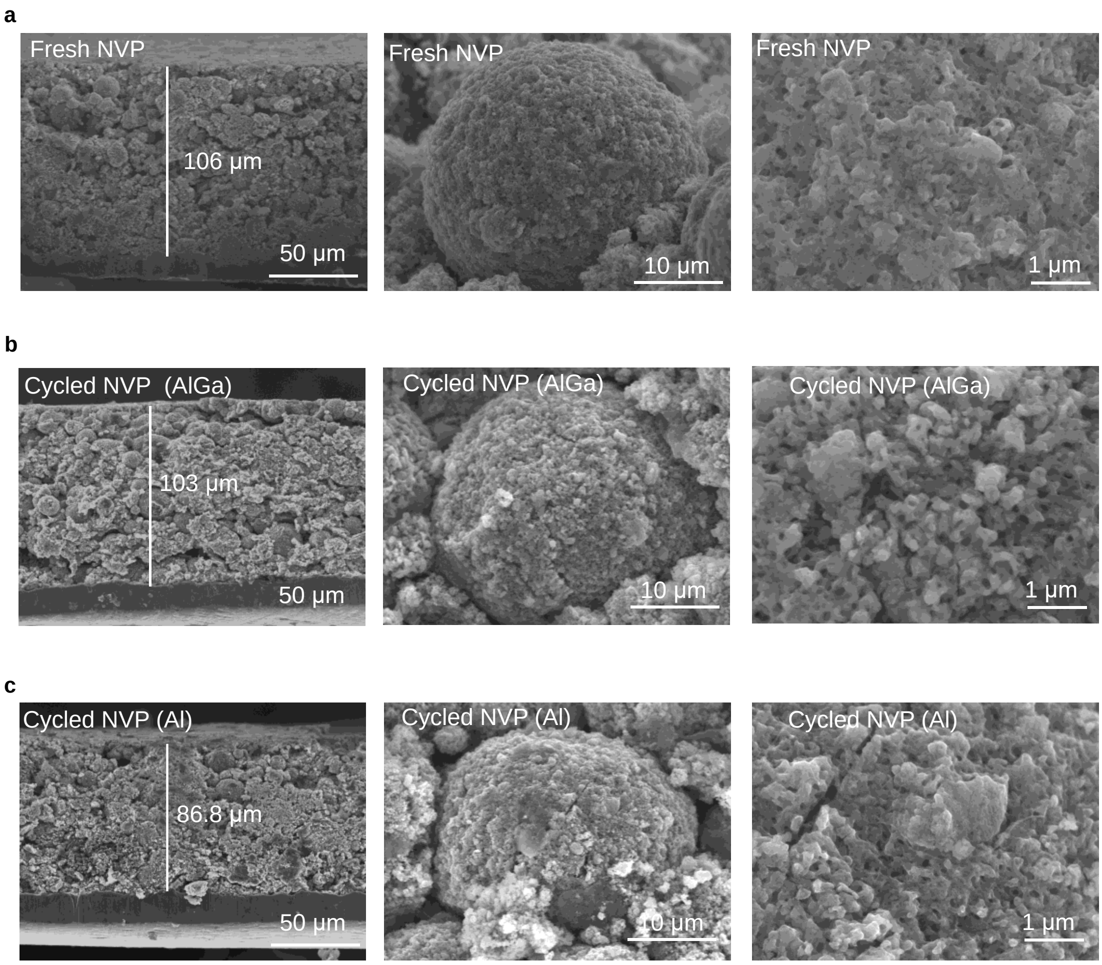

Supplementary Fig. 24 The cross-sectional SEM images of the fresh NVP (a), and NVP after cycled with AlGa (b) and Al (c). The NVP cathode massing loading is 11.4 mg

cm^{-2}

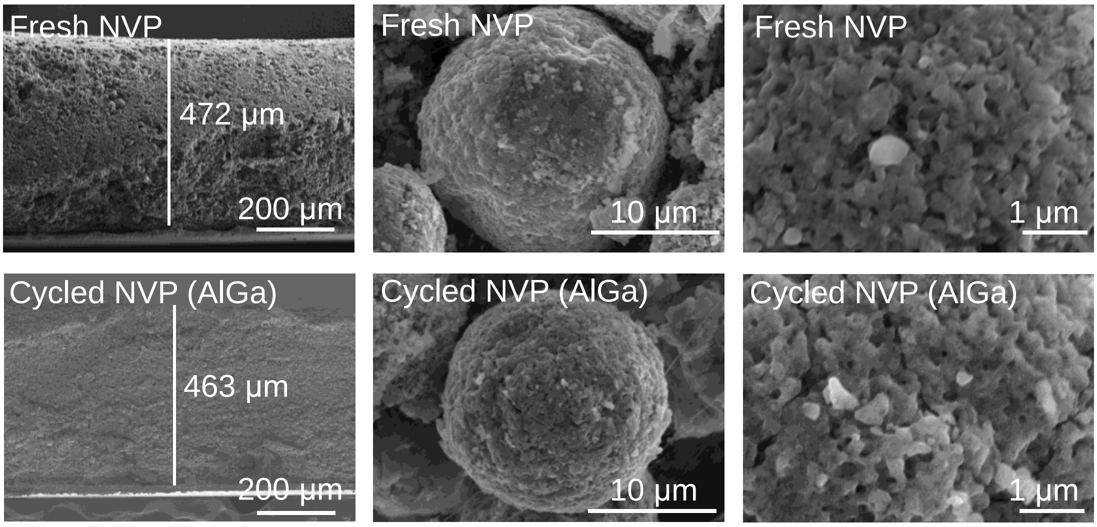

Supplementary Fig. 25 The cross-sectional SEM images of the fresh NVP(a), and NVP after cycled with AlGa (b). The NVP cathode massing loading is 47.4 mg cm^{-2}

Comment 7:

Please provide SEM image of the AlGa current collector after cycling in the anode-free Na battery to demonstrate its structural stability.

Response:

We sincerely thank you for the careful and rigorous comments.

SEM characterization of the AlGa current collector after cycling in the anode-free Na battery has been conducted after both cycles and extended cycling up to 860 cycles, as shown in Supplementary Fig. 20–21. Owing to dissolution of Al^{3+} during long-term cycling, localized surface peeling can be observed on the AlGa current collector. Nevertheless, the overall surface remains relatively flat, indicating that the AlGa current collector maintains acceptable structural integrity even after prolonged cycling.

Furthermore, energy-dispersive X-ray spectroscopy (EDS) mapping reveals that the elemental distribution across the AlGa surface is largely preserved after cycling. Notably, the Na signal on the cycled surface is extremely weak, suggesting negligible accumulation of residual sodium. This observation indicates that the formation of "dead Na" is effectively suppressed on the AlGa current collector during long-term cycling. These results demonstrate that despite surface evolution, the AlGa current collector retains its functional stability and does not induce detrimental sodium accumulation during long-term anode-free cycling.

We have added the content in revised manuscript (see page 13, lines 301-305).

"After 100 and 860 cycles (Supplementary Fig. 20–21), slight surface exfoliation is observed on the AlGa current collector due to Al dissolution, while the overall surface remains essentially flat. EDS mapping confirms uniform elemental distribution and negligible Na accumulation, indicating suppression of "dead Na" and overall structural stability during long term cycling."

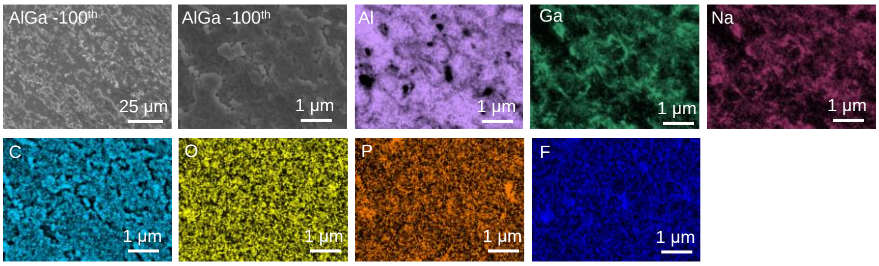

Element	Atom(%)
Al	62.12
Ga	2.64
Na	0.78
C	22.38
O	8.94
P	0.01
F	3.13
total	100

Supplementary Fig. 20 SEM images of Na deposition AlGa(a) and Al(b) after 1500 cycles at 1mA cm^{-2} for 1 mAh cm^{-2}

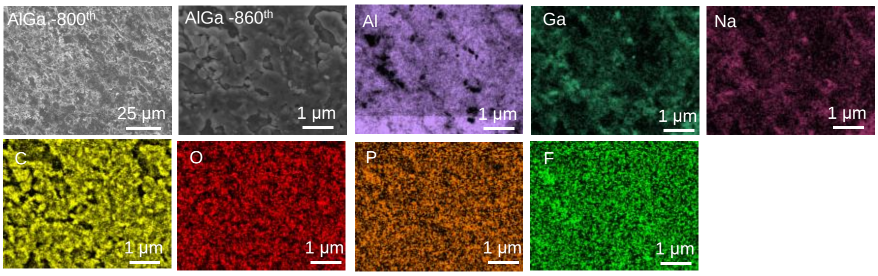

Element	Atom(%)
Al	64.80
Ga	1.85
Na	0.66
C	21.21
O	8.51
P	0.00
F	2.96
total	100

Supplementary Fig. 21 SEM images of Na deposition AlGa(a) and Al(b) after 1500 cycles at 1mA cm^{-2} for 1 mAh cm^{-2}

Comment 8:

Some minor errors should be corrected.

In Figure 3d, "mAh cm-1" should be "mAh cm-2"

In Page 2 and 3, "… achieving simultaneous control over dendrite-free Na deposition and in situ self-replenishment of active Na remains …" should be "… achieving simultaneous control over dendrite-free Na deposition and in situ self-replenishment of active Na remain ..."

In Page 3, "The AlGa||Na exhibites …" should be "The AlGa||Na exhibits …"

In Page 3, "… which can easily extent to other anode-free battery systems" should be "… which can easily extend to other anode-free battery systems"

In Page 4, "The electrochemical processes on the Al and AlGa surface …" should be "The electrochemical processes on the Al and AlGa surfaces …"

In Page 5, "… the deposited Na on the Al and AlGa current collector …" should be "… the deposited Na on the Al and AlGa current collectors …"

In Page 7, "… elemental mapping tests (Figure 2b-c; Supplementary Fig. 5) shows … " should be "… elemental mapping tests (Figure 2b-c; Supplementary Fig. 5) show … "

In Page 17, "… the AlGa||NVP cell deliver …" should be "… the AlGa||NVP cell delivers …"Improve readability by avoiding abbreviations in the abstract.

Response: We sincerely thank you for the careful and rigorous comments. We have revised the relevant sections of the manuscript accordingly to address the issues raised above. And in the abstract, we remove the unnecessary abbreviations. Figure 3d has

been corrected as shown below.

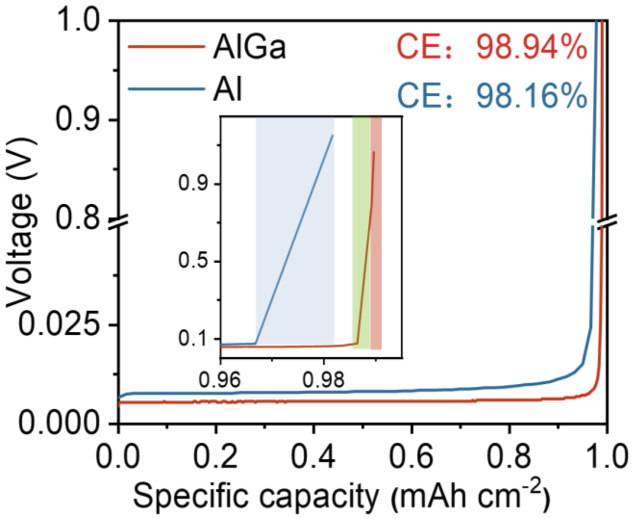

Fig. 3 (d) The galvanostatic potential versus capacity curves of Na stripping at 1 mA cm^{-2} with 1 mAh cm^{-2}

Comment 9:

This direction of anode-free Na batteries is highly promising for future practical high-energy density batteries. Several highly related papers about functionalized Al collectors were most recently published, which do not undermine the novelty of this work significantly, for example, A functionalized porous Al current collector enables high-energy density anode-free Na batteries, Science Advances, 2025, 11, eadx7124; Growing Sodiophilic ZnO Nanorod Arrays on Al Substrate for High-Energy-Density Anode-Free Na Batteries, Journal of the American Chemical Society, https://doi.org/10.1021/jacs.5c16420

Response:

We appreciate your insightful recommendations. We have carefully studied the two suggested articles.

The first work (Science Advances, 2025, 11, eadx7124) reports the design of a functionalized porous aluminum current collector to regulate Na. The controllable porous architecture, effectively reduces structural stress and suppresses dendritic Na formation by lowering the local current density and homogenizing Na^+ flux.

The second work (J. Am. Chem. Soc. 2025, 147, 46440–46448) focuses on highly reversible sodium plating/stripping behavior achieved on D-ZnO nanorod array–modified Al substrates, which exhibit a low nucleation overpotential and dendrite-free sodium morphology.

Both works provide valuable insights into the deposition behavior of Na on current collectors. These studies have been highly instructive for our work, particularly in clarifying the mechanistic description of Na deposition and in adopting more precise and cautious language in the discussion. We have revised the manuscript to better emphasize the Na deposition mechanism. The suggested references have been cited at

appropriate locations in the revised manuscript (see References 45 and 46).

Reference

1. Rajagopalan M, Bhatia MA, Tschopp MA, Srolovitz DJ, Solanki KN. Atomic-scale analysis of liquid-gallium embrittlement of aluminum grain boundaries. Acta Materialia 73, 312-325 (2014).

2. Ludwig W, Bellet D. Penetration of liquid gallium into the grain boundaries of aluminium: a synchrotron radiation microtomographic investigation. Materials Science and Engineering: A 281, 198-203 (2000).

3. Lv X*, et al.* Sodium–Gallium Alloy Layer for Fast and rReversible Sodium Deposition. *SusMat* **2**, 699-707 (2022).

4. Wan TH, Saccoccio M, Chen C, Ciucci F. Influence of the Discretization Methods on the Distribution of Relaxation Times Deconvolution: Implementing Radial Basis Functions with DRTtools. Electrochimica Acta 184, 483-499 (2015).

5. Maradesa A, et al. Advancing electrochemical impedance analysis through innovations in the distribution of relaxation times method. Joule 8, 1958-1981 (2024).

Responses to reviewer 4:

The manuscript by Zheng et al. "Engineered Al Drives Na Self-Replenishment Realizes 200 Wh kg^{-1} Anode Free Batteries" discusses an interesting approach to preventing dendrite formation and dead Na in reservoir-free sodium ion batteries. To this end, the authors apply Ga to the aluminium current collector. This accumulates at the grain boundaries of the aluminium and, according to a cited paper, ensures that no covering layer of aluminium oxide forms in these areas. As a consequence, according to the authors' hypothesis, Al^{3+} ions are dissolved out of the current collector during cell cycling. The paper examines full cells with commercial NVP as the cathode material. It is demonstrated that the cells can operate for over 2500 cycles without short-circuiting. This is indeed a remarkable advance, although the charging rate of 0.3 C is very conservative.
The energy density achieved here is remarkably high for SIB and represents a noteworthy improvement and is worth being published in a high prestigious journal.
Although the connections are clearly illustrated with appropriate figures, the work has a few weaknesses that need to be addressed before publication. I have the following comments, which are in the order in which they appear in the manuscript:

Response:

Thank you for reviewing our manuscript and for highlighting the remaining issues. We have carefully revised the manuscript to address the points that could potentially lead to misunderstanding, and we have provided additional experimental details to facilitate clearer interpretation and reproducibility of the reported measurements. In addition, formatting issues throughout the manuscript have been corrected. The author contributions have also been re-discussed and revised to accurately reflect each author's role. We hope that these revisions satisfactorily address your concerns. Please see reply to the following comments.

Comment 1:

I cannot follow the argument of the introduction "The dissolution alters the local charge distribution in the electrolyte, thereby inducing spontaneous migration of Na^+ from the bulk electrolyte toward the cathode to compensate." - Why should this happen? Al^{3+} can simply be dissolved at a lower rate due to its three times higher charge in comparison to Na^+. Or instead, anions are attracted.

Response:

We appreciate your insightful comments. and agree that the original description was confusing and could lead to alternative interpretations.

Our intention was to describe an electrochemical process. The dissolution of Al^{3+} in the electrolyte alters the local coordination environment through interactions with anions and solvent molecules, perturbing the Na^+ solvation equilibrium and promoting the dissociation of coordinated Na^+, which subsequently migrates toward the cathode for charge compensation. It is not intended to express the low dissolution rate of Al^{3+}.

Therefore, we have made the following modifications to this sentence. We have modified the content in revised manuscript (see page 3, lines 62-65).

Comment 2:

Please comment further on this statement "Further, the Ga penetration along the grain boundaries generates amorphous regions, which weaken the Al crystal structure and facilitate Al atoms gradual dissolution." – Is Ga alloying with Al? If it is only adsorbed in the grain boundaries, the Al should not get amorphous.

Response:

Thank you for your valuable comment.

We appreciate your comment and agree that our original description was not sufficiently precise. We have therefore revised the description and reinterpreted the results based on the XRD data to ensure a more accurate discussion.

First, according to the Al–Ga phase diagram, Ga and Al can form an Al–Ga solid solution at the preparation temperature of 30 °C, which is consistent with our experimental conditions. In the XRD results, the (200) plane shows a peak at a slightly lower angle, which can be attributed to the entry of some large-sized Ga into the small-sized Al lattice to form an Al–Ga solid solution.

In addition, we observe that the intensities of the (111) and (200) planes decrease, while the (220) and (311) planes increase significantly. We acknowledge that these changes in peak intensity may arise from the formation of partial Al–Ga solid solution and associated crystallographic reorientation, rather than serving as direct evidence for amorphization.

We therefore agree that the term "amorphous regions" was overly strong based on the current experimental evidence. Our original intention was to emphasize Ga enrichment and local structural disorder at the Al grain boundaries, rather than the formation of a fully amorphous phase. Accordingly, we have revised both the original statement and the corresponding XRD discussion to avoid overinterpretation and to ensure a more rigorous description. We have modified the content in revised manuscript (see page 4, lines 91-94; page 8, line 180-186).

The revised description is as follows:

"Further, Ga penetration along the Al grain boundaries leads to Ga enrichment and partial Al–Ga alloying. This grain boundary modification weakens the local Al bonding environment and facilitates Al atoms gradual dissolution."

"The (200) plane shows a peak with a slightly smaller angle, which can be attributed to the entry of some large-sized Ga into the small-sized Al lattice to form an Al–Ga solid solution. The (200) plane shows a peak with a slightly smaller angle, which can be attributed to the entry of some large-sized Ga into the small-sized Al lattice to form an Al－Ga solid solution. The (111) and (200) planes show a decrease in intensity, whereas the (220) and (311) planes show a significant increase in intensity, which probably arises from the formation of a partial Al－Ga solid solution and the associated crystallographic reorientation."

Comment 3:

If Al^{3+} is dissolved it might be also shuttling to the cathode. Can it be found in the cathode after cycling? – This might easily be proven by ToF-SIMS depth profiling, which is highly sensitive.

Response:

Thank you very much for your valuable comments.

After 30 cycles, the AlGa||NVP battery was disassembled. ToF-SIMS depth profiling of the NVP surface revealed abundant Al-related fragments: Al^{+}, AlO^{-}, AlO_2^{-}, and $AlOC^{-}$ ion fragments (Supplementary Fig. 26). This further confirms the dissolution of Al^{3+} form AlGa collector. We have added the content in revised manuscript (see page 17, lines 385-386).

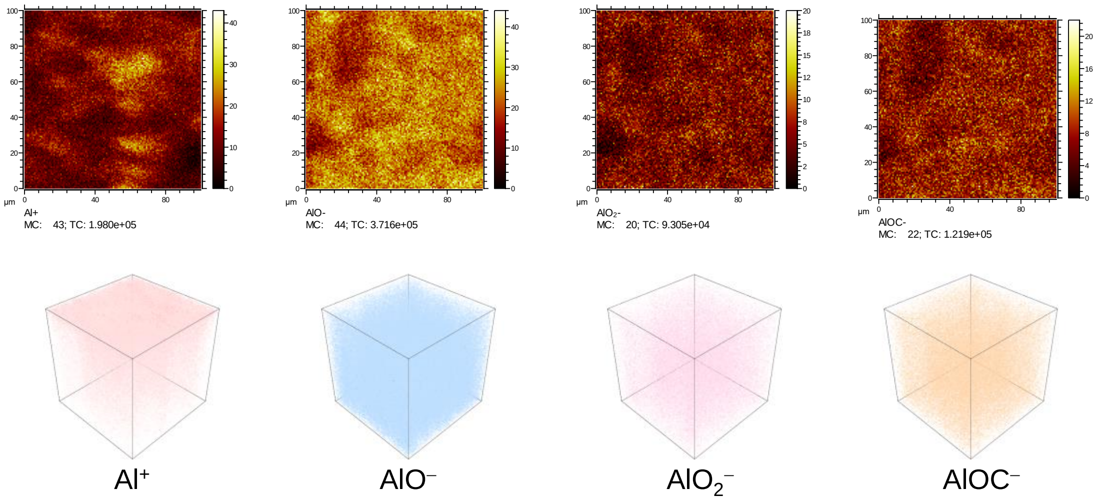

Instrument: IONTOF M6-Hybrid	
Acquisition phase	
Ion species	Bi3 (positive mode)
Energy	30 keV
Ion current	0.522 pA
Raster size	100 μm x 100 μm
Mass range	0 - 270 u
Mode	All-Purpose (Hybrid)
Analysis time	600 s (spectra)
Sputter phase (for cleaning)	
Ion species	Ar
Energy/Current	500 eV / 62.12 nA
Raster size	300 μm x 300 μm
Sputtering time	600 sec

Instrument: IONTOF M6-Hybrid	
Acquisition phase	
Ion species	Bi3 (negative mode)
Energy	30 keV
Ion current	0.519 pA
Raster size	100 μm x 100 μm
Mass range	0 - 270 u
Mode	All-Purpose (Hybrid)
Analysis time	600 s (spectra)
Sputter phase (for cleaning)	
Ion species	Ar
Energy/Current	500 eV / 62.460 nA
Raster size	300 μm x 300 μm
Sputtering time	600 sec

Supplementary Fig. 26 TOF-SIMS 3D reconstruction of the sputtered volume of several secondary ion fragments on the cycled NVP matched with AlGa surface.

Comment 4:

"Ga is mechanically coated onto Al foil" – How exactly was this carried out? I cannot find any information about this in the experimental section.

Response:

Thank you very much for your valuable comments.

We acknowledge that the original wording was unclear. To avoid confusion, we have rephrased this description and revised the corresponding section in the main text. "The AlGa was prepared by uniformly brushing liquid Ga onto an Al foil." We have modified the content in revised manuscript (see page 7, lines 154).

Comment 5:

Figure 2c) Scale bars are missing

Response:

Many thanks for your correction. Figure 2c has been corrected as shown below.

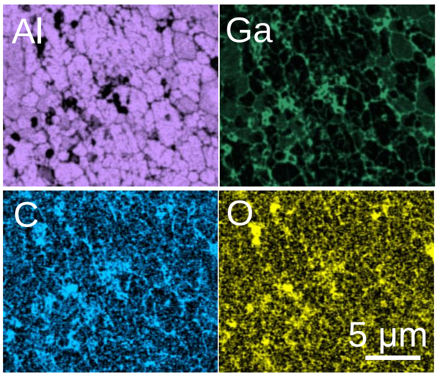

Fig. 2 (c) EDS mappings of AlGa

Comment 6:

Figure 2d) No scale bars are given (dimensions and intensities (colour code)).

Response:

Thank you for your valuable comments. Figure 2d has been corrected as shown below.

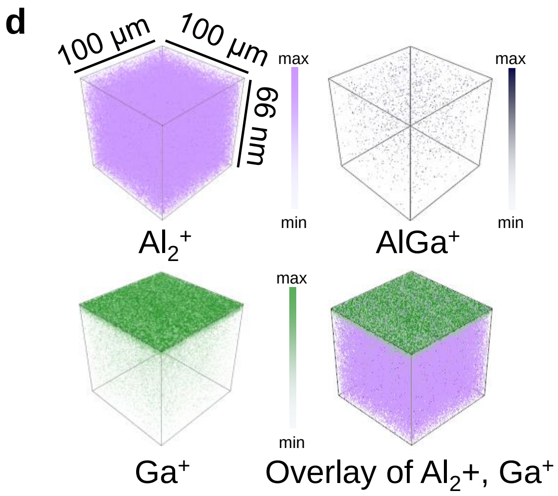

Fig. 2 (d) TOF-SIMS depth profiles of AlGa.

Comment 7:

The experimental section has significant gaps in the materials characterisation part. The information provided is insufficient to repeat the experiments/analyses or to assess the quality of the work within the scope of reviewing this manuscript. For example, the rather complex ToF-SIMS analysis is used. Here, only the device type is named, followed by two confusing pieces of information.

"In addition, the three-dimensional structure of AlGa was characterized by time-of-flight secondary ion mass spectrometry (TOF-SIMS) (IONTOF M6), which was equipped with a 30 kV Bi+ analysis beam and 2000 eV Ar ion sputtering."

Fact is that the device is equipped with a 30 kV liquid metal bismuth ion source. Several primary ion species are available to choose from. The same applies to the 2 kV Ar source. Here, several energies can be selected. The beam is then an Ar+ beam. Essential information is missing about the method itself in order to repeat the measurements. No statement was made about the ablation rate for metallic Ga mentioned in the caption to Supplementary Fig. 7. With so few measured data points, the depth profiling should be repeated even with a lower energy sputter beam. For data reporting in ToF-SIMS I can recommend the paper by Graham and Gamble (https://doi.org/10.1116/6.0002633). In section IV they state what is essential following the rules of good scientific practice. This statement applies to all other analytical techniques used.

Response:

We agree that a comprehensive description of the materials characterization methods is essential for the work, particularly the ToF-SIMS analysis. According to the recommended reporting guidelines for ToF-SIMS data (Graham and Gamble, Biointerphases, 2023), key parameters such as the specific primary ion species, sputter beam conditions, and depth profiling details should be explicitly provided. Due to space limitations in the manuscript, we have included the above test parameters in the corresponding supporting information (Supplementary Fig. 7, S22, S23, S26).

Regarding deep profiling, we acknowledge that high-energy sputtering initially led to a rapid sputtering rate and a limited number of data points, which resulted in inaccurate depth estimation of the Ga metal layer. Therefore, the sputtering energy was reduced, and two independent depth-profiling experiments were performed to reliably determine the Ga layer thickness.

Since the AlGa sample is predominantly composed of Al, with Ga present only in a minor amount and preferentially enriched at grain boundaries, the sputtering depth was calculated based on the sputter rate of Al. Using a 500 eV Ar^+ sputtering source and the instrument's built-in calculation parameters, the sputter rate of Al was estimated to be 0.1109 nm s^{-1}. Two depth-profiling measurements performed at different locations yielded highly consistent depth profiles (Supplementary Fig. 7).

From the depth profiles, the Ga signal decreases to a stable baseline at approximately 100 s, indicating that the Ga-enriched layer is largely removed beyond this sputtering time. Accordingly, the thickness of the Ga layer is estimated to be approximately 11.1 nm (100 s × 0.1109 nm s^{-1}). It should be noted that the presence of

Ga may slightly influence the local sputtering rate; however, this effect is expected to be limited given the low overall Ga content.

The revised description is as follows: We have added the content in revised manuscript (see page 7, lines 169, page 22-23, lines 510-516).

"The thickness is about 11.1 nm (Supplementary Fig. 7)."

"TOF-SIMS (IONTOF M6) was employed to characterize the three-dimensional structure of AlGa and SEI/CEI, using a 30 kV Bi^+ primary ion beam for AlGa and a 30 kV $Bi3^+$ beam for SEI/CEI analysis, combined with 500 eV Ar^+ sputtering. The analysis area was 100 μm × 100 μm, while the sputtering area was 300 μm × 300 μm. Detailed experimental parameters for each measurement are provided in the corresponding Supplementary Figures."

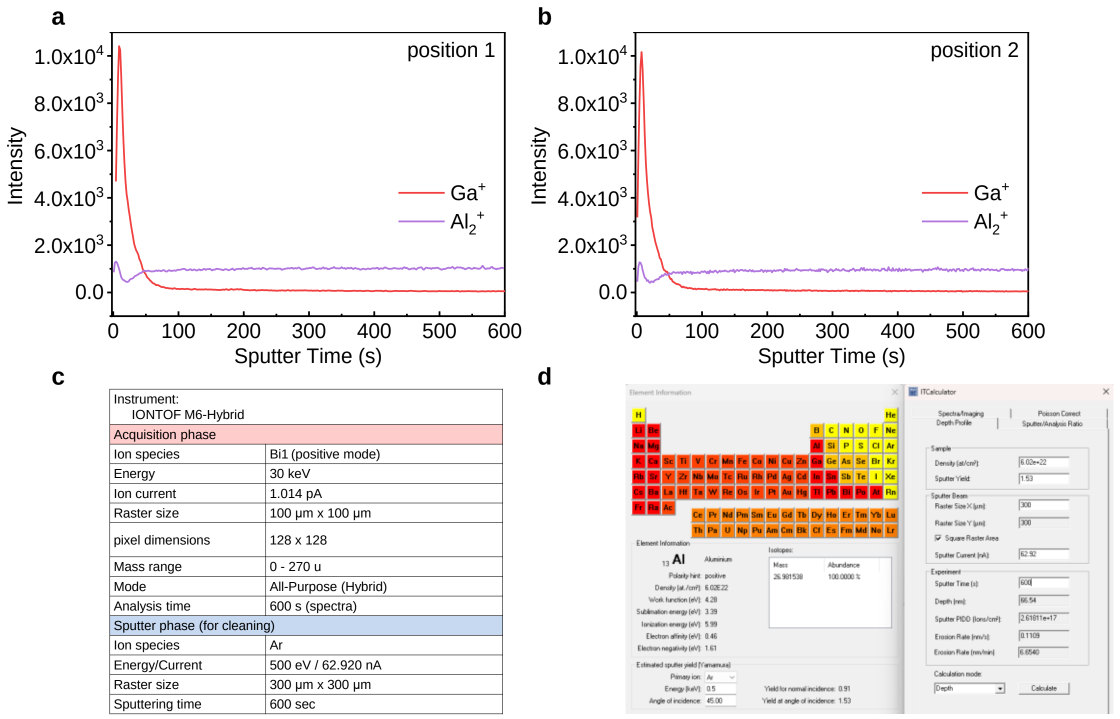

FigureS7 (a-b) The variation of fragment species intensity with sputtering time. (c) Actual conditions of TOF-SIMS testing. (d) Calculation of the Al etching rate corresponding to the set conditions within the instrument. For metal Al, the etching rate is 0.1109 nm s^{-1}. At an etching time of approximately 100 s, there is no significant change in Ga^+ ions. Therefore, the thickness of the Ga layer is estimated to be approximately 11.1 nm.

Comment 8:

The initials in the Author contribution section cannot be attributed to the authors. I have the feeling that this section has never been edited. Given the multitude of methods used, there are also doubts as to whether one person carried out all the experiments and analyses.

Response:

Thank you for your valuable comments. We reanalyzed and discussed the contributions of all authors and revised the Author Contributions section accordingly.

D.N.L. and C.X.W. conceived and supervised the project. D.N.L. and X.Y.Z. conceived the idea and designed the experiments. X.Y.Z. and D.P.Y. carried out the material synthesis, electrochemical measurements. X.Y.Z., D.P.Y., D.N.L. analyzed the data. F.T., D.N.L. and C.X.W. assisted with data interpretation and provided critical discussions. X.Y.Z. wrote the original draft of the manuscript. X.Y.Z., D.P.Y., F.T., D.N.L. and C.X.W. revised and refined the manuscript. All authors discussed the results, and approved the final version.

Itemized Responses/Revisions to the original manuscript

Responses to reviewer 1:

The authors have made substantial efforts to address my previous concerns by providing additional electrochemical data, nanoindentation results, and TOF-SIMS analysis. I appreciate the revisions and the expanded discussion. However, after carefully examining the responses and the newly provided data, several important issues remain insufficiently resolved. Some mechanistic interpretations are still not fully convincing, and certain conclusions appear to be overextended relative to the presented evidence.

Response:

Thank you very much for your valuable comments on our work. In response, we have attempted a quantitative analysis of Ga migration. While precise quantification is challenging, the analysis reasonably captures the nanoscale nature of the migration. And further addressed the impact of $NaGa_4$ alloying–induced volume expansion and Al dissolution on structural stability. In addition, we have provided additional results and clarifications regarding the role of dissolved Al in charge compensation and in modifying the solvation structure.

Comment 1:

While the authors argue that Ga is confined at grain boundaries and exists in low concentration, the core issue regarding its low melting point has not been fundamentally addressed. The resolution of EDS is extremely limited, which makes it impossible to rule out the occurrence of micro-scale liquid migration and unable to prove that Ga does not undergo localized rearrangement. The nanoindentation argument is not fully conclusive, as hardness variations can arise from multiple factors beyond Ga redistribution (e.g., surface corrosion, SEI coverage, residual stress). Therefore, the stability of Ga under electrochemical and thermal stress remains only indirectly supported.

Response:

We sincerely thank the reviewer for the insightful comment regarding the intrinsically low melting point of Ga (29.76 °C) and the potential for liquid migration under electrochemical and thermal perturbations. We fully acknowledge this concern and agree that Ga, due to its low melting point, may exhibit a certain degree of mobility. Based on the cross-sectional SEM images and TOF-SIMS depth profiling results before and after cycling, we also observe that Ga can migrate on the microscale. However, it is important to note that the amount of Ga introduced in this system is extremely limited (~0.023 mg cm^{-2} relative to Al). Therefore, even if local migration occurs, it is unlikely to induce catastrophic redistribution under electrochemical or thermal conditions.

Firstly, we focus on vertical migration (from top to bottom) through cross-sectional analysis. EDS line-scan measurements and TOF-SIMS depth profiling were performed on AlGa electrodes after 50 and 100 cycles (with Na stripped), together with a control

same batch sample stored without cycling (AlGa standing). The sectional SEM observations show that Ga remains enriched near the surface after cycling, with a gradual decrease toward the bulk (Supplementary Fig.22). We note that EDS analysis may introduce fluctuations due to its limited spatial resolution and sensitivity to local surface flatness.

To obtain more reliable depth-resolved information, TOF-SIMS sputtering analysis was further conducted. The results reveal that the Ga-enriched region increases from ~9.3 nm (calculated as described in the Supplementary Fig. 23–25) to ~28.8 nm after 50 cycles, although the presence of an SEI layer may introduce some uncertainty in absolute depth determination, the nanoscale migration range remains reasonable. In addition, both Ga^+ and $NaGa^+$ signals decrease significantly after ~250 s of sputtering, and the Ga signal becomes nearly constant after ~500 s (57.5 nm). Notably, $NaGa^+$ species are enriched in the near-surface region (approximately 4.6 – 18 nm from the top surface, as highlighted by the green shaded area in Supplementary Fig. 24), whereas the Ga^+ signal is strongest within the topmost ~4.6 nm. This suggests that Na–Ga formed at the surface undergoes reversible alloying/dealloying, whereas the subsurface Na-Ga alloy phase acts as a stabilizing layer that effectively suppresses further inward migration of Ga. For 100 cycles, it can be observed that the Ga^+-enriched region and the $NaGa^+$-enriched region remain nearly unchanged in position, indicating that the migration of Ga remains essentially stable during cycling.

Moreover, as a low-melting-point metal, Ga is reported to potentially offer a certain degree of interface adaptability, enabling it to dynamically adapt to the fluctuations in volume and stress during plating/deposition processes [1-4].

Therefore, although Ga migration does occur in AlGa, it is confined to a nanoscale range (tens of nanometers). Furthermore, we examined the Na deposition morphology after 50, 100, and 850 cycles by plating 1 mAh cm^{-2} Na onto the AlGa surface (Supplementary Fig.26). The deposited Na remains smooth and uniform even after prolonged cycling, indicating that the nanoscale redistribution of Ga does not deteriorate Na plating behavior.

We have modified the content in revised manuscript (see page 14-15, lines 320-338.) The revised description is as follows:

"Furthermore, the evolution of the AlGa current collector and the Na deposition behavior after prolonged cycling were investigated. The microscale migration of Ga has been carefully considered. Vertical migration was investigated through EDS line scans and TOF-SIMS depth profiling, which were performed on AlGa after 50 and 100 cycles (with Na stripped), along with a control sample stored without cycling. Cross-sectional SEM shows that Ga remains enriched near the surface after cycling, gradually decreasing toward the bulk (Supplementary Fig. 22), although some fluctuations may arise from the limited resolution of EDS. To obtain more reliable depth information, TOF-SIMS analysis was conducted (Supplementary Fig. 23–25). The Ga-enriched region increases from ~9.3 nm to ~28.8 nm after 50 cycles, indicating nanoscale migration, despite some uncertainty from the SEI layer. Both Ga^+ and $NaGa^+$ signals decrease significantly after ~250 s sputtering, with Ga becoming nearly constant beyond ~500 s (~57.5 nm). Notably, $NaGa^+$ is enriched in the near-surface region

(~4.6–18 nm), while Ga^+ is strongest within the topmost ~4.6 nm, suggesting reversible alloying at the surface and the formation of a subsurface Na–Ga layer that suppresses further inward migration. After 100 cycles, the positions of the Ga^+ and $NaGa^+$ enriched regions remain nearly unchanged, indicating stable Ga distribution during cycling. The Na deposition morphology after 50, 100, and 850 cycles by plating 1 mAh cm^{-2} Na onto the AlGa surface were tested (Supplementary Fig.26). The deposited Na remains smooth and uniform even after prolonged cycling."

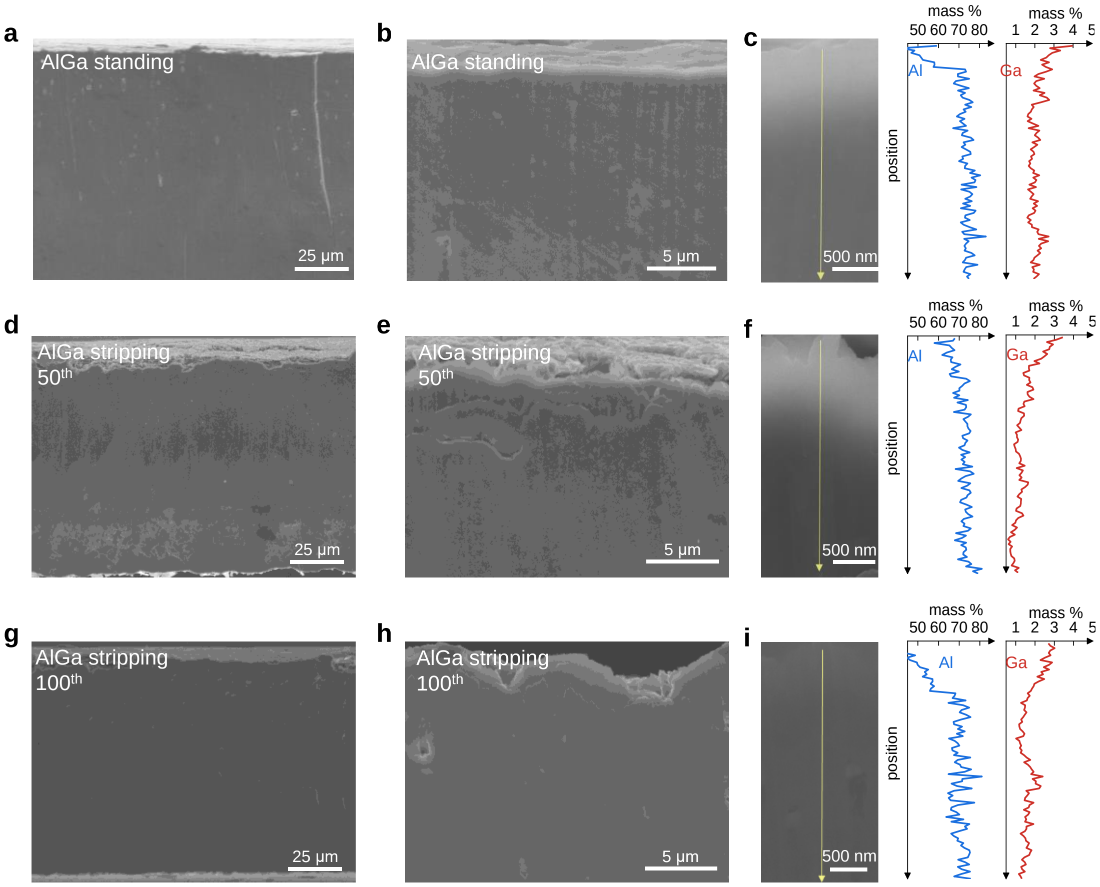

Supplementary Fig. 20 Cross-sectional SEM images of AlGa after 8 days of standing (a, b) and the corresponding EDS line scan of Al and Ga (c), showing an atomic ratio of 71.98:1.24. After 50 cycles (d, e), the corresponding line scan (f) shows an Al:Ga ratio of 66.74:0.90. After 100 cycles (g, h), the line scan (i) shows an Al:Ga ratio of 69.19:1.10.

AlGa standing

a

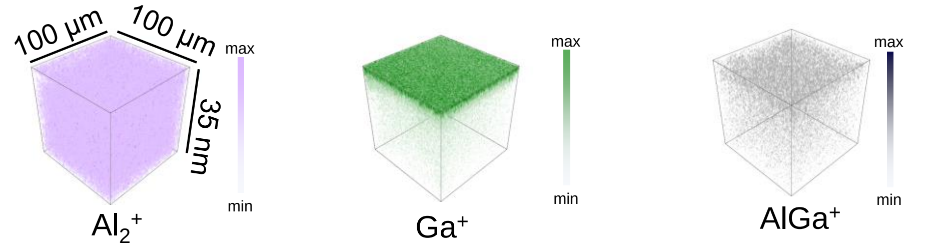

b

**Instrument: IONTOF M6-Hybrid**	
**Acquisition phase**	
**Ion species**	**Bi1 (positive mode)**
**Energy**	**30 keV**
**Ion current**	**0.870 pA**
**Raster size**	**100 μm x 100 μm**
**Mass range**	**0 - 270 u**
**Mode**	**All-Purpose (Hybrid)**
**Analysis time**	**300 s (spectra)**
**Sputter beam**	
**Ion species**	**Ar**
**Energy/Current**	**500 eV / 66.130 nA**
**Raster size**	**300 μm x 300 μm**
**Sputtering time**	**300 sec**

d

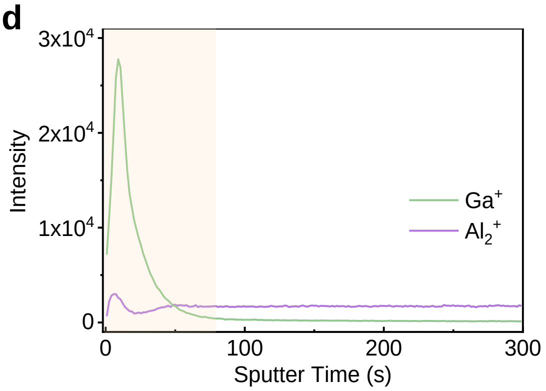

c

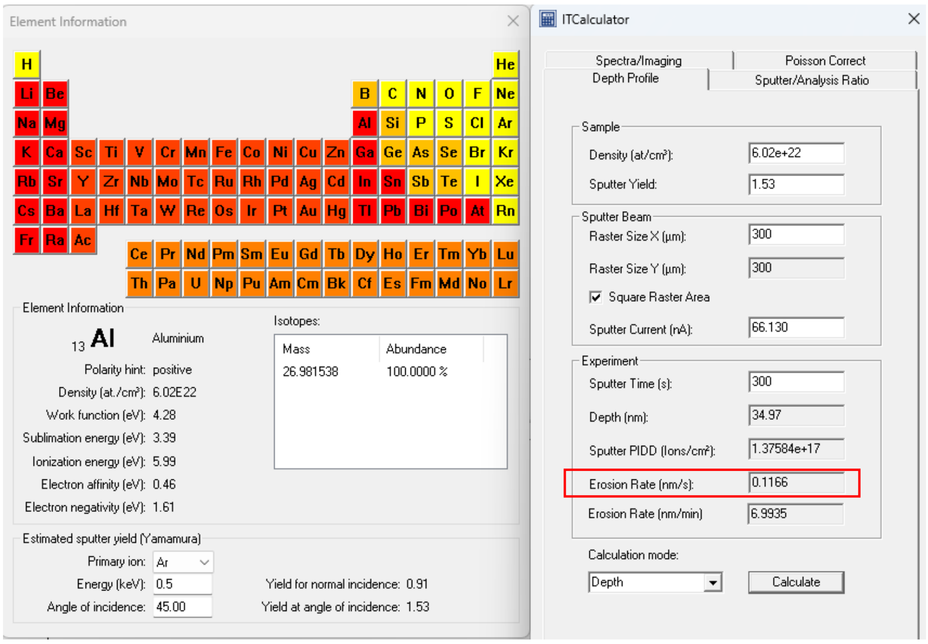

Supplementary Fig. 21 (a) TOF-SIMS depth profiles of AlGa after 8 days of standing. (b) Actual conditions of TOF-SIMS testing. (c) Calculation of the Al etching rate corresponding to the set conditions within the instrument. For metal Al, the etching rate is 0.1166 nm s^{-1}. At an etching time of approximately 80 s, there is no significant change in Ga^+ ions. Therefore, the thickness of the Ga layer is estimated to be approximately 9.3 nm (0.1166 × 80 = 9.3). (d)The variation of fragment species intensity with sputtering time.

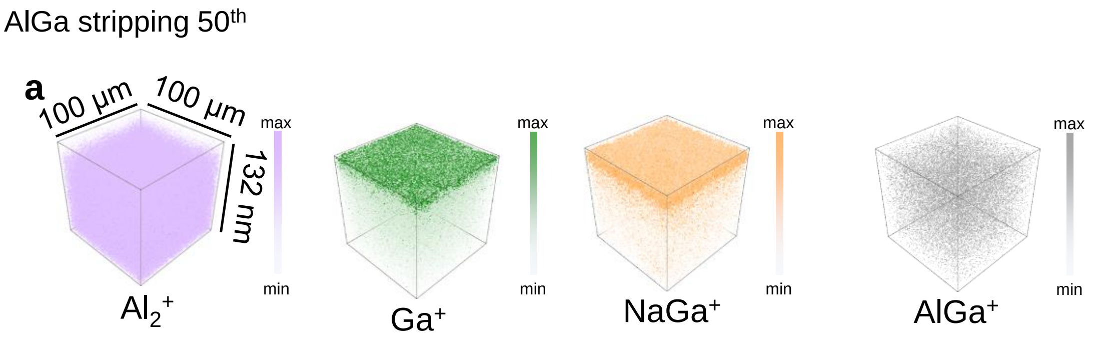

b

Instrument: IONTOF M6-Hybrid	
**Acquisition phase**	
Ion species	Bi1 (positive mode)
Energy	30 keV
Ion current	0.900 pA
Raster size	100 μm x 100 μm
Mass range	0 - 270 u
Mode	All-Purpose (Hybrid)
Analysis time	1147 s (spectra)
**Sputter beam**	
Ion species	Ar
Energy/Current	500 eV / 65.240 nA
Raster size	300 μm x 300 μm
Sputtering time	1147 sec

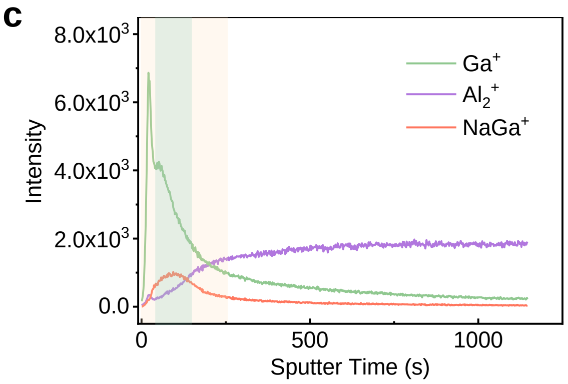

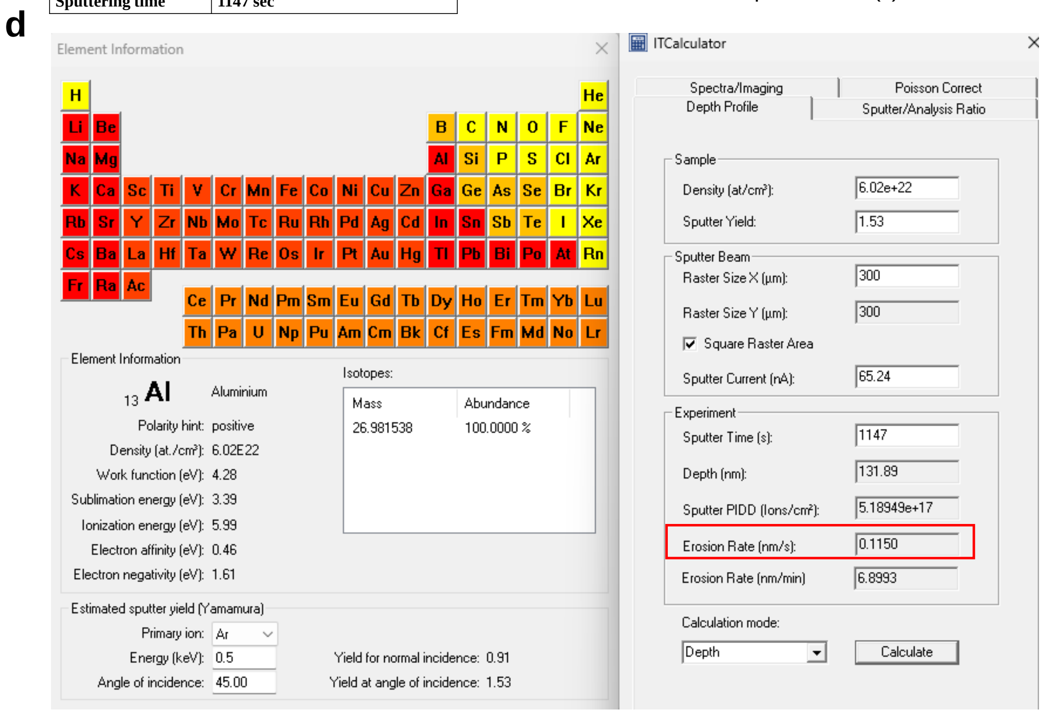

Supplementary Fig. 22 (a) TOF-SIMS depth profiles of AlGa after 50 cycles. (b) Actual conditions of TOF-SIMS testing. (c) Calculation of the Al etching rate corresponding to the set conditions within the instrument. For metal Al, the etching rate is 0.1150 nm s^{-1}. At an etching time of approximately 250 s, there is no significant change in Ga^+ ions. Therefore, the thickness of the Ga layer is estimated to be approximately 9.3 nm (0.1150 × 250 = 28.8 nm). (d)The variation of fragment species intensity with sputtering time.

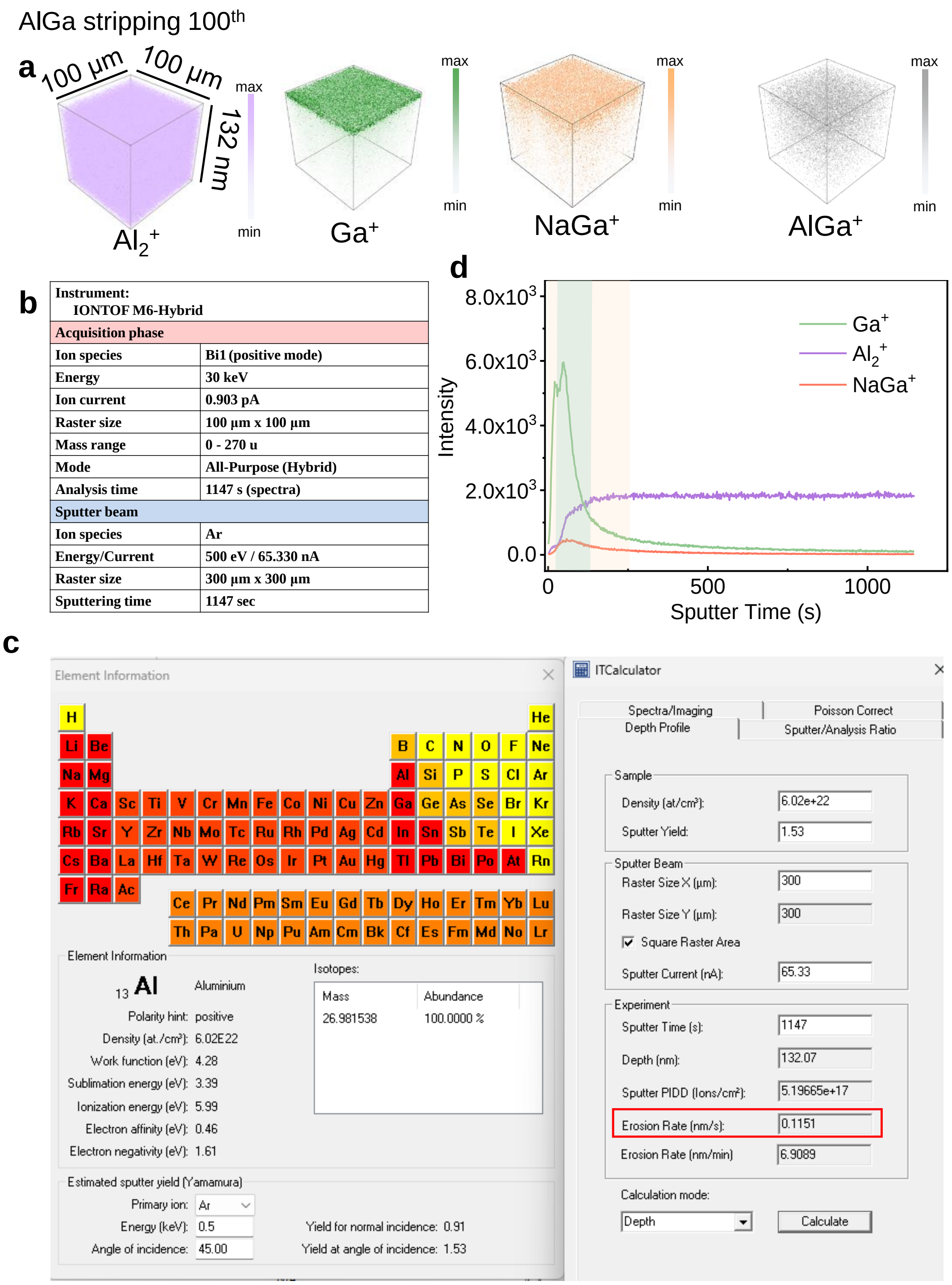

Supplementary Fig. 23 (a) TOF-SIMS depth profiles of AlGa after 100 cycles. (b) Actual conditions of TOF-SIMS testing. (c) Calculation of the Al etching rate corresponding to the set conditions within the instrument. For metal Al, the etching rate is 0.1151 nm s^{-1}. At an etching time of approximately 250 s, there is no significant change in Ga^+ ions. Therefore, the thickness of the Ga layer is estimated to be approximately 28.8 nm (0.1151 × 250 = 28.8 nm). (d)The variation of fragment species intensity with sputtering time.

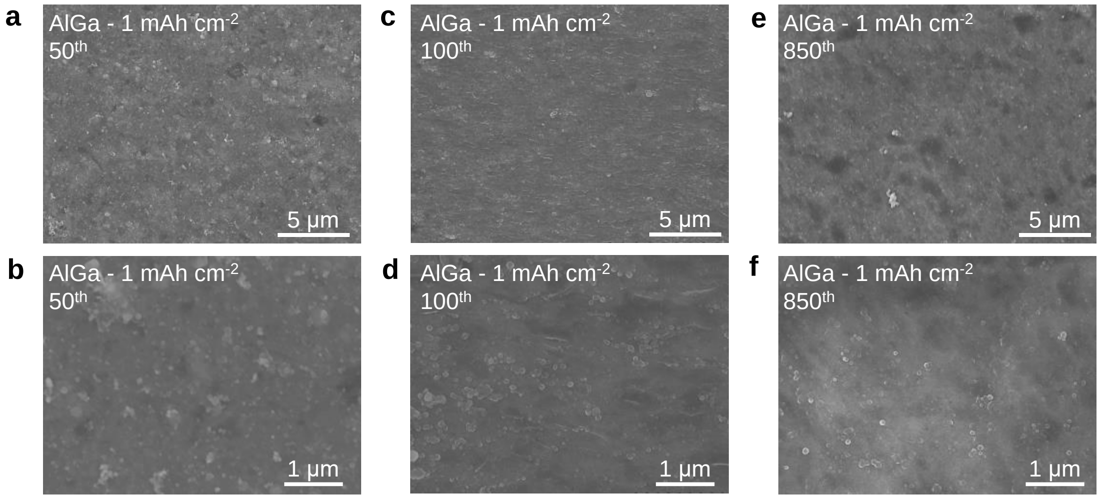

Supplementary Fig. 24 SEM images of Na deposition on AlGa at 1 mA cm^{-2} for 0.1 mAh cm^{-2} after 50(a-b), 100(c-d), 850(e-f) cycles.

Reference:

1. Zhang H, *et al.* An integrated self-healing anode assembled via dynamic encapsulation of liquid metal with a 3D Ti3C2Tx network for enhanced lithium storage. *Energy & Environmental Science* **15**, 5240-5250 (2022).
2. Xie Z, *et al.* Liquid metal interface mechanochemistry disentangles energy density and biaxial stretchability tradeoff in composite capacitor film. *Nature Communications* **15**, 7817 (2024).
3. Fu M, *et al.* Optimizing Na plating/stripping by a liquid sodiophilic Ga-Sn-In alloy towards dendrite-poor sodium metal anodes. *Energy Storage Materials* **63**, 103020 (2023).
4. Zhang S, *et al.* Liquid metal batteries for future energy storage. *Energy & Environmental Science* **14**, 4177-4202 (2021).

Comment 2:

The CV results clearly reveal detectable Na-Ga alloying peaks, confirming the formation of $NaGa_4$, as acknowledged by the authors in their response to Comment 3. A weak alloying peak does not necessarily correspond to a small volume change. The peak intensity in CV primarily reflects electrochemical kinetics and current response, rather than directly quantifying the magnitude of structural expansion. Even if the extent of alloy formation is limited, the associated localized volume changes may still induce significant mechanical stress, particularly at grain boundaries.

Response:

We sincerely thank the reviewer for the careful comment. We acknowledge that the CV results only indicate that the alloying of $NaGa_4$ is limited, but cannot directly quantify the extent of volume expansion. For alloy-type anodes, Ga generally exhibits relatively moderate volume change compared with conventional alloying materials. The volume expansion was estimated based on the molar volume (derived from density), yielding a value of ~28% (Table R1), calculated as:

$$V_{\mathrm{expand}} = \frac{V_{\mathrm{NaGa_4}} - 4V_{\mathrm{Ga}}}{V_{\mathrm{NaGa_4}}} = \frac{60.6 - 4 \times 11.8}{60.6} \approx 28\%$$

This volume change is significantly smaller than that of typical alloy anodes such as Sn (~420%)[1] and Bi (250%)[2]. Moreover, the amount of Ga used in our system is extremely low (~ 0.023 mg cm^{-2}). In addition, Ga tends to diffuse preferentially along favorable grain boundaries rather than uniformly throughout the bulk, which avoids large-scale structural collapse. Meanwhile, part of Ga can form an Al–Ga solid solution, further reducing the effective amount of $NaGa_4$ formed.

Importantly, the formation of Na–Ga alloy also suppresses the further inward diffusion of Ga, contributing to interfacial stability. Furthermore, Rietveld refinement of the initial and after 20 cycles (Supplementary Fig.16) samples shows negligible change in the lattice parameters of Al, indicating small structural distortion of the current collector.

As pointed out in Comment 1, the hardness obtained from nanoindentation reflects a combination of multiple factors, including Ga distribution, residual stress, surface Al dissolution, and SEI coverage (Supplementary Table 2). The relatively small change in hardness before and after cycling suggests that the overall mechanical state of the surface remains largely unchanged, implying that the impact of Na–Ga alloy-induced volume expansion on the current collector is limited.

Finally, even considering Ga migration, Na–Ga alloy formation, and partial Al dissolution, the Na deposition morphology remains smooth after prolonged cycling (50, 100, and 850 cycles). This further proves that the volume expansion of $NaGa_4$ does not have detrimental effect on the Na deposition process.

We have modified the content in revised manuscript (see page 12, lines 256-264.) The revised description is as follows:

"In AlGa collector, due to the extremely low content of Ga (~ 0.023 mg cm^{-2}) and the presence of some Al–Ga solid solution. The volume expansion caused by a trace amount of $NaGa_4$ has a relatively small impact on the current collector of the AlGa. Nanoindentation results (Supplementary Table 2) show that the hardness of AlGa after cycling is comparable to that before cycling. The relatively small change in hardness before and after cycling suggests that the overall mechanical state of the surface remains largely unchanged, implying that the impact of Na–Ga alloy-induced volume expansion on the current collector is limited. Furthermore, Rietveld refinement of AlGa before and after 20 cycles (Supplementary Fig. 16) reveals only minor changes in the lattice parameters of Al, indicating that the combined effects of Al dissolution and Na–Ga alloying-induced volume expansion exert a limited impact on the structural integrity of the AlGa collector."

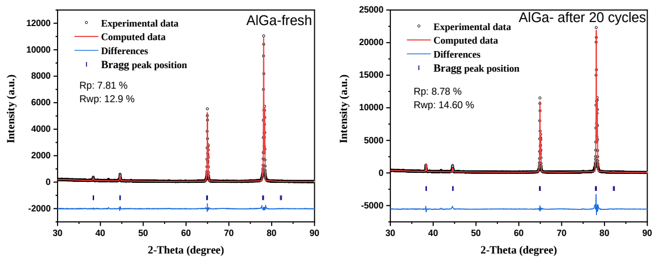

c

	a(Å)	b(Å)	c(Å)
Al-fresh	4.06552	4.06552	4.06552
Al after 20cycles	4.06464	4.06464	4.06464

Figure S16 Rietveld refinement of the fresh and after 20 cycles AlGa.

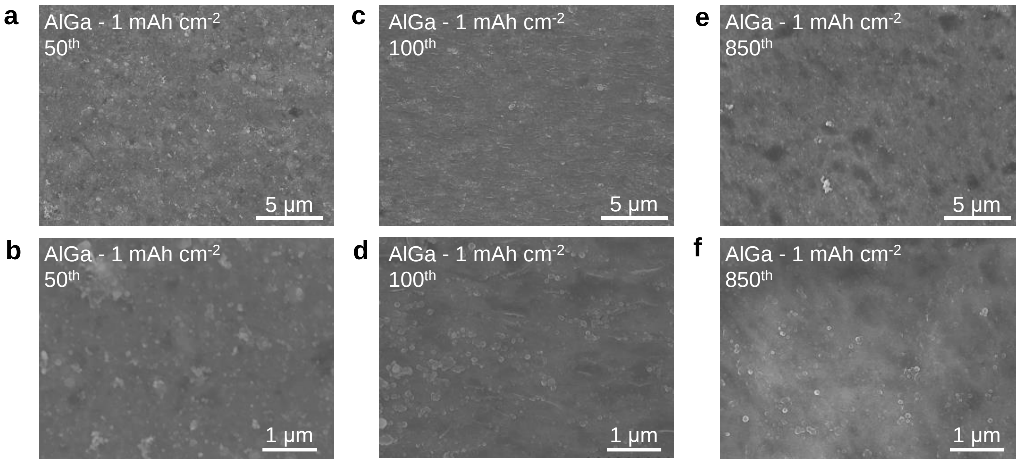

Supplementary Fig. 24 SEM images of Na deposition on AlGa at 1 mA cm^{-2} for 0.1 mAh cm^{-2} after 50(a-b), 100(c-d), 850(e-f) cycles.

Table R1 The parameters required for volume expansion calculation.

	Ga	NaGa[4]
density (g cm^{-3})	5.91	5
molar mass (g mol^{-1})	69.7	302.8
molar volume (cm^3 mol^{-1})	11.8	60.6

Supplementary Table 2 The hardness (H-IT) of Al, AlGa, H-AlGa, Al-800℃ and AlGa after 100 cycled.

Sample	H-IT (Gpa)	Average H-IT (Gpa)
Al	0.807	0.804
	0.783	
	0.821	
AlGa	0.280	0.314
	0.262	
	0.401	
H-AlGa	0.572	0.586
	0.566	
	0.621	
Al-800℃	0.543	0.521
	0.507	
	0.515	
AlGa after 100 cycled	0.373	0.311
	0.229	
	0.332	

The H-IT data were determined in accordance with ISO 14577.

Reference:

1. Deori D*, et al.* Self-Healing, Ga-Sb Alloy Confined Sn Anode for Stable Na-Ion Storage in Glyme Based Electrolytes. *Small* **21**, 2504185 (2025).
2. Wang Y*, et al.* Facile Galvanic Replacement Construction of Bi@C Nanosheets Array as Binder-Free Anodes for Superior Sodium-Ion Batteries. *Advanced Energy Materials* **14**, 2401833 (2024).

Comment 3:

The authors attribute the capacity increase to accelerated Al3+ dissolution, which supposedly facilitates Na+ utilization. If additional electrons originate from Al oxidation (Al → Al3+ + 3e-), this represents an irreversible parasitic reaction rather than reversible battery capacity, and this would contradict the claim of structural stability and raise concerns about long-term durability.

Response:

We fully acknowledge that the dissolution of Al^{3+} could, in principle, affect the structural integrity of the current collector. To quantitatively evaluate this effect, we measured the Al concentration in the electrolyte after long cycling by Inductively Coupled Plasma-Atomic Emission Spectrometry (ICP-OES), which is ~0.013 mol L^{-1}. This corresponds to an equivalent Al thickness of ~34 nm for a 12 mm diameter current collector, which is only ~0.034% of the Al foil thickness (~100 μm), indicating a negligible impact on the electrode's structural stability.

In addition, the dissolution of Al may also play a beneficial role. First, the released Al^{3+} can provide additional charge compensation, facilitating Na^{+} insertion on the cathode side. Second, Al^{3+} may act as a functional additive in the electrolyte, modifying the solvation structure and influencing the formation of the SEI and CEI.

In the AlGa current collector, the electrochemical performance enhancement can be attributed to both the significantly improved reversibility of Na and the minor contribution from Al dissolution, which together enhance the overall Na^{+} utilization efficiency. Furthermore, cross-sectional observations show no obvious structural degradation after cycling, further confirming that the limited Al dissolution does not compromise the mechanical integrity of the current collector (Supplementary Fig.22).

We have modified the content in revised manuscript (see page 6, lines 142-146.) The revised description is as follows:

"To quantitatively evaluate this effect, as shown in Supplementary Table 1, the Al concentration in the electrolyte after long cycling was measured to be ~0.013 mol L^{-1} by Inductively Coupled Plasma-Atomic Emission Spectrometry (ICP-OES). Meanwhile, the dissolved Al^{3+} may also play a beneficial role by contributing to charge compensation and modifying the solvation structure, thereby influencing SEI and CEI formation."

Supplementary Table 1 ICP-OES test result.

Sample	Al element concentrationin the test solution (mg/L)

Diglyme	0. 193
After cycled electrolyte	5.558

Based on ICP-OES results, the Al concentration in the electrolyte after cycling is 5.580 mg L^{-1}, while the Al concentration in the pristine diglyme is 0.193 mg L^{-1}. After subtracting the background contribution, the net Al concentration is 5.387 mg L^{-1}. This value corresponds to a sample obtained by diluting 30 μL of electrolyte to 2 mL. Therefore, the original Al concentration in the electrolyte is calculated to be 0.359 g L^{-1} (5.387 × 2000 / 30 = 359.1 mg L^{-1} = 0.359 g L^{-1}). Given the molar mass of Al (27 g mol^{-1}), the corresponding molar concentration is approximately 0.013 mol L^{-1} (0.359 / 27 ≈ 0.013 mol L^{-1}).

The total amount of dissolved Al in 30 μL electrolyte is thus 3.9×10^{-7} mol (0.013 mol L^{-1} × 30 μL), corresponding to a mass of 10.5 μg ($3.9 \times 10^{-7} \times 27 = 1.05 \times 10^{-5}$ g). Using the density of Al (ρ = 2.70 g cm^{-3}), the corresponding volume of dissolved Al can be estimated. For an electrode with a diameter of 12 mm (area ≈ 1.13 cm^2), the equivalent thickness is calculated to be ~34 nm (1.05×10^{-5} / 2.7 / 1.13 ≈ 3.4×10^{-6} cm ≈ 34 nm).

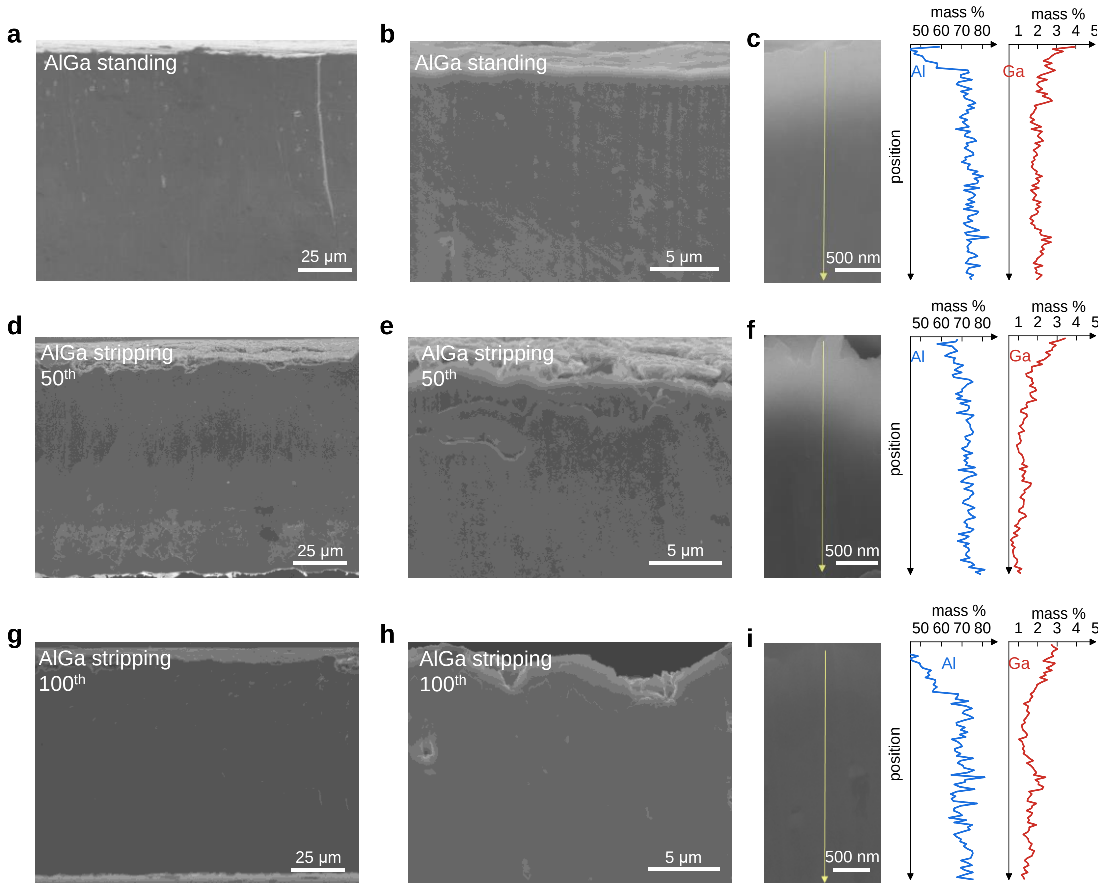

Supplementary Fig. 22 Cross-sectional SEM images of AlGa after 8 days of standing (a, b) and the corresponding EDS line scan of Al and Ga (c), showing an atomic ratio of 71.98:1.24. After 50 cycles (d, e), the corresponding line scan (f) shows an Al:Ga ratio of 66.74:0.90. After 100 cycles (g, h), the line scan (i) shows an Al:Ga ratio of

69.19:1.10.

Comment 4

I am still confused about "The dissolved Al3+ in the electrolyte alters the local coordination environment through interactions with anions and solvent molecules perturbing the Na+ solvation equilibrium and promoting the dissociation of coordinated Na+, which subsequently migrates toward the cathode for charge compensation." Why could this happen? This requires a clear electrochemical driving force. It is not evident how local perturbation of Na^+ solvation by Al^{3+} would independently generate a net Na^+ flux toward the cathode beyond that already imposed by the external current.

Response:

Thank you for your careful inspection and constructive comments. We apologize for confusion caused by our previous description. Our intention was to describe an electrochemically process. Specifically, Al^{3+} dissolution at the anode is accompanied by the release of electrons, which are transported through the external circuit to the cathode. To maintain overall charge neutrality of the electrochemical system, these electrons are consumed by Na^+ insertion at the cathode, thereby inducing the migration of Na^+ from the bulk electrolyte toward the cathode.

Meanwhile, to investigate the change in solvation structure, the electrolyte after cycling was collected. Prior to the NMR measurement, the cycled electrolyte (~30 μL) was diluted with 1 mL of pristine electrolyte (1 M $NaPF_6$ in diglyme) to obtain a sufficient volume for analysis. Although this dilution inevitably reduces the concentration of dissolved Al^{3+} and may partially weaken their influence on the solvent environment, the observed spectral shifts still clearly indicate the interaction between Al^{3+} and the ether oxygen atoms of diglyme.

Compared with the pristine electrolyte, the diluted cycled electrolyte shows shifts in the ^{13}C NMR spectra, indicating that the dissolved Al^{3+} interact with the ether oxygen atoms of diglyme. In diglyme, three types of C atoms can be identified (C_a, C_b, C_c, as shown in Supplementary Fig.1). For C_a, a slight downfield shift is observed. This can be attributed to the interaction between Al^{3+} and the terminal ether oxygen (O_a), which withdraws electron density toward the oxygen atom and consequently decreases the electron density around the adjacent carbon. Whereas, when Al^{3+} interacts with the inner ether oxygen (O_b), the electron redistribution along the C–O–C framework results in an opposite effect. Due to the competing electron-withdrawing effects from both sides, electron density is redistributed toward the C–C bond, leading to a increase in the electron density around C_b and C_c, which results in an upfield shift in their corresponding peaks.

Therefore, the dissolution of Al^{3+} into the electrolyte leads to its interaction with diglyme molecules, which may modify the solvation structure of Na^+ by altering the coordination environment between diglyme and Na^+. This change in solvation equilibrium could facilitate the dissociation of coordinated Na^+.

Therefore, we have made the following modifications to this sentence (see page 3,

lines 62-67; page 4 lines 99-103).

"The dissolved Al^{3+} interacts with the ether oxygen of diglyme, potentially altering the Na^+ solvation structure and facilitating the dissociation of coordinated Na^+. Meanwhile, the dissolution provides additional electrons to the external circuit, which electrochemically couples with Na^+ insertion at the cathode to maintain charge neutrality. This drives the migration of Na^+ from the electrolyte toward the cathode to compensate."

"In addition, the dissolution of Al^{3+} into the electrolyte leads to its interaction with diglyme molecules (Supplementary Fig. 1), which may modify the solvation structure of Na^+ by altering the coordination environment between diglyme and Na^+. This change in solvation equilibrium could facilitate the dissociation of coordinated Na^+."

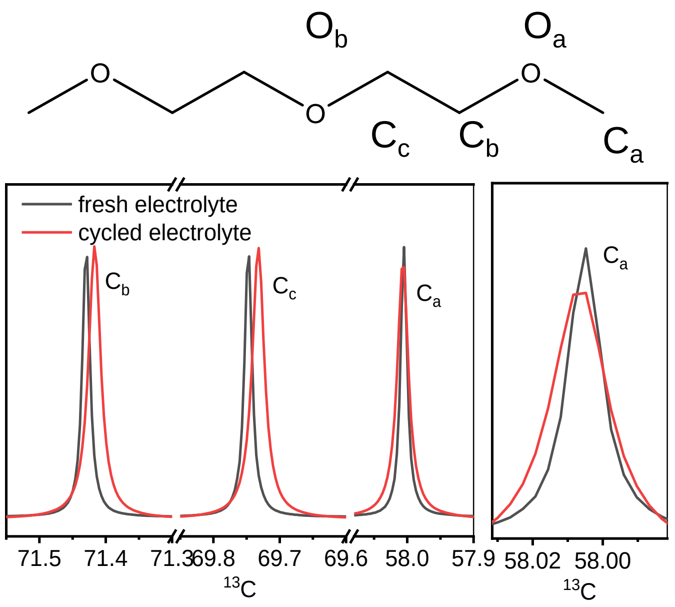

Supplementary Fig. 1 ^{13}C NMR of fresh electrolyte and cycled electrolyte.

Responses to reviewer 2:

The author has well addressed all my comments. I suggest to accept the contribution at the current status.

Response:

We sincerely thank the reviewer for the positive evaluation of our work and for recommending the acceptance of our manuscript.

Responses to reviewer 3:

I have looked at the revised manuscript and the response letter to all 4 reviewers. Overall, the authors have done a good job in revision. I am happy to recommend its acceptance of this excellent work for publication in Nat comm. The only minor comment I have now is that there are some minor errors in the references in year number, volume number, page number/article number, etc.
These can be corrected during the proof stage.

Response:
We sincerely thank the reviewer for the careful evaluation of our revised manuscript and for recommending the acceptance of our work. We have corrected some minor errors in the year number, volume number, page number/article number, etc. of the references.

Responses to reviewer 4:

The authors have satisfactorily addressed the raised concerns and revised the manuscript accordingly. However, two minor issues have come to my attention that should be addressed prior to publication:

Response:

We sincerely thank the reviewer for the positive evaluation of our work. The two minor errors have been corrected as requested.

Comment 1

In the abstract you write The AlGa||$Na_3V_2(PO_4)_3$ pouch cell". Please give the full cell including the electrolyte. This makes it clearer for the reader. This also counts for the other mentioned cells.

Response:

Thank you for your careful inspection and constructive comments. We have added the electrolyte near the battery so that readers can understand it more clearly. We have modified the content in revised manuscript (see page 1, line25.) The revised description is as follows:

"The AlGa||$Na_3V_2(PO_4)_3$ pouch cell delivers 88.7% capacity retention after 100 cycles in electrolyte (1 M $NaPF_6$ in diglyme)."

Comment 2

In Supplementary Fig. 22 your write in the tables "Sputter phase (for cleaning)". Please correct to "sputter beam". I am wondering why you are writing "cleaning". It is standard depth profiling, and you want to show the 3D SEI composition. Or am I wrong? – Same for Fig. 26

Response:

Thank you for your careful comments. We acknowledge that the writing "Sputter phase (for cleaning)" might cause confusion. What we actually mean is to construct a 3D image of SEI through deep analysis. We have already changed it to "sputter beam". We have modified the content in Supplementary Fig. 8, 23-25, 29-30, 33.

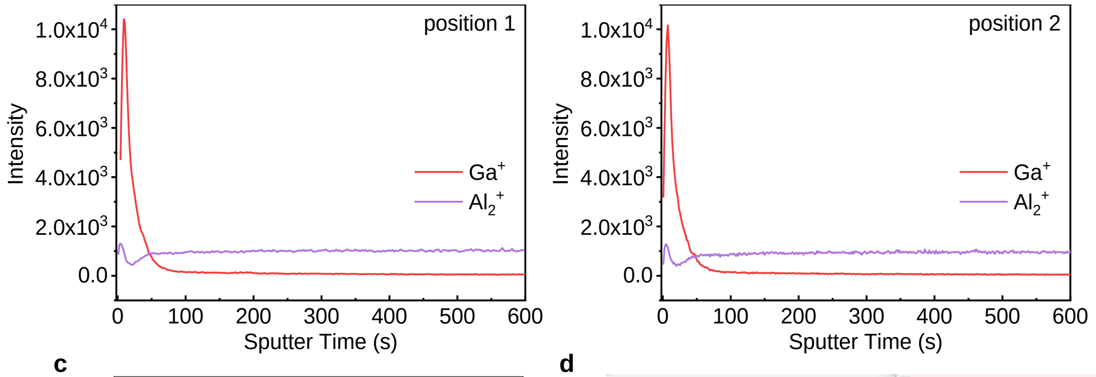

c

Instrument: IONTOF M6-Hybrid	
Acquisition phase	
Ion species	Bi1 (positive mode)
Energy	30 keV
Ion current	1.014 pA
Raster size	100 µm x 100 µm
pixel dimensions	128 x 128
Mass range	0 - 270 u
Mode	All-Purpose (Hybrid)
Analysis time	600 s (spectra)
Sputter beam	
Ion species	Ar
Energy/Current	500 eV / 62.920 nA
Raster size	300 µm x 300 µm
Sputtering time	600 sec

d

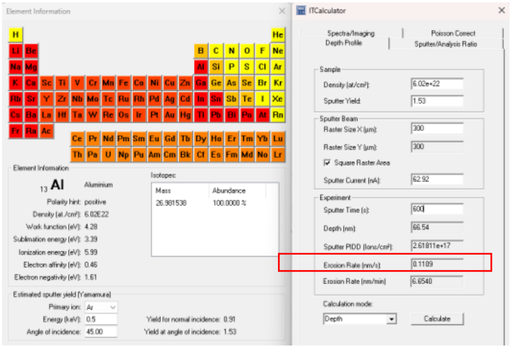

Supplementary Fig. 8 (a-b) The variation of fragment species intensity with sputtering time. (c) Actual conditions of TOF-SIMS testing. (d) Calculation of the Al etching rate corresponding to the set conditions within the instrument. For metal Al, the etching rate is 0.1109 nm s^{-1}. At an etching time of approximately 100 s, there is no significant change in Ga^{+} ions. Therefore, the thickness of the Ga layer is estimated to be approximately 11.1 nm.

AlGa standing

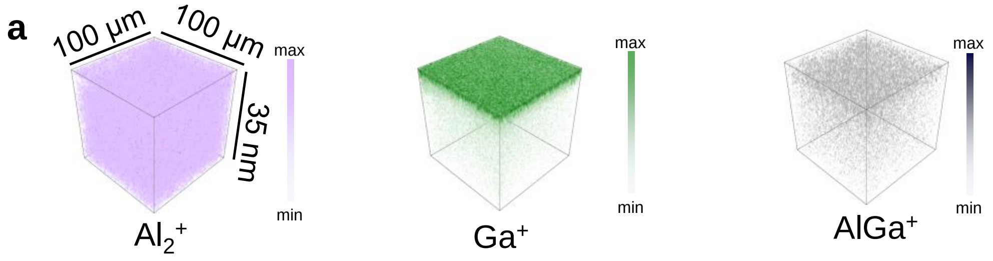

b

Instrument: IONTOF M6-Hybrid	
**Acquisition phase**	
Ion species	Bi1 (positive mode)
Energy	30 keV
Ion current	0.870 pA
Raster size	100 μm x 100 μm
Mass range	0 - 270 u
Mode	All-Purpose (Hybrid)
Analysis time	300 s (spectra)
**Sputter beam**	
Ion species	Ar
Energy/Current	500 eV / 66.130 nA
Raster size	300 μm x 300 μm
Sputtering time	300 sec

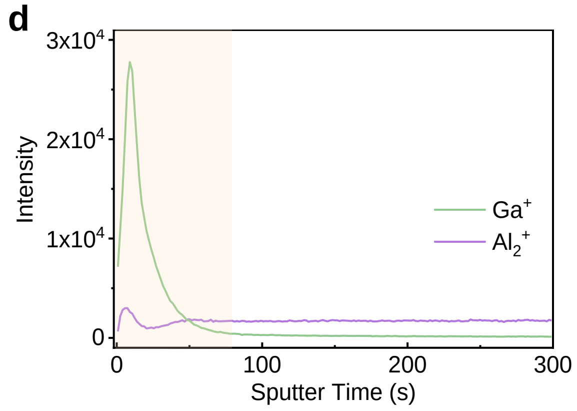

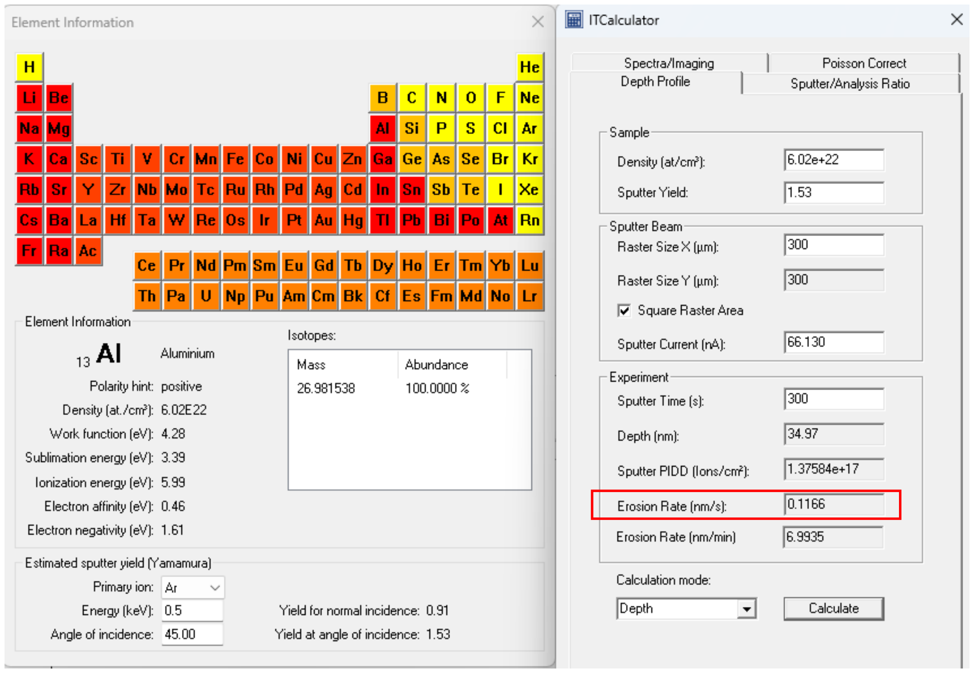

Supplementary Fig. 23 (a) TOF-SIMS depth profiles of AlGa after 8 days of standing. (b) Actual conditions of TOF-SIMS testing. (c)The variation of fragment species intensity with sputtering time. (d) Calculation of the Al etching rate corresponding to the set conditions within the instrument. For metal Al, the etching rate is 0.1166 nm s^{-1}. At an etching time of approximately 80 s, there is no significant change in Ga^{+} ions. Therefore, the thickness of the Ga layer is estimated to be approximately 9.3 nm (0.1166 × 80 = 9.3).

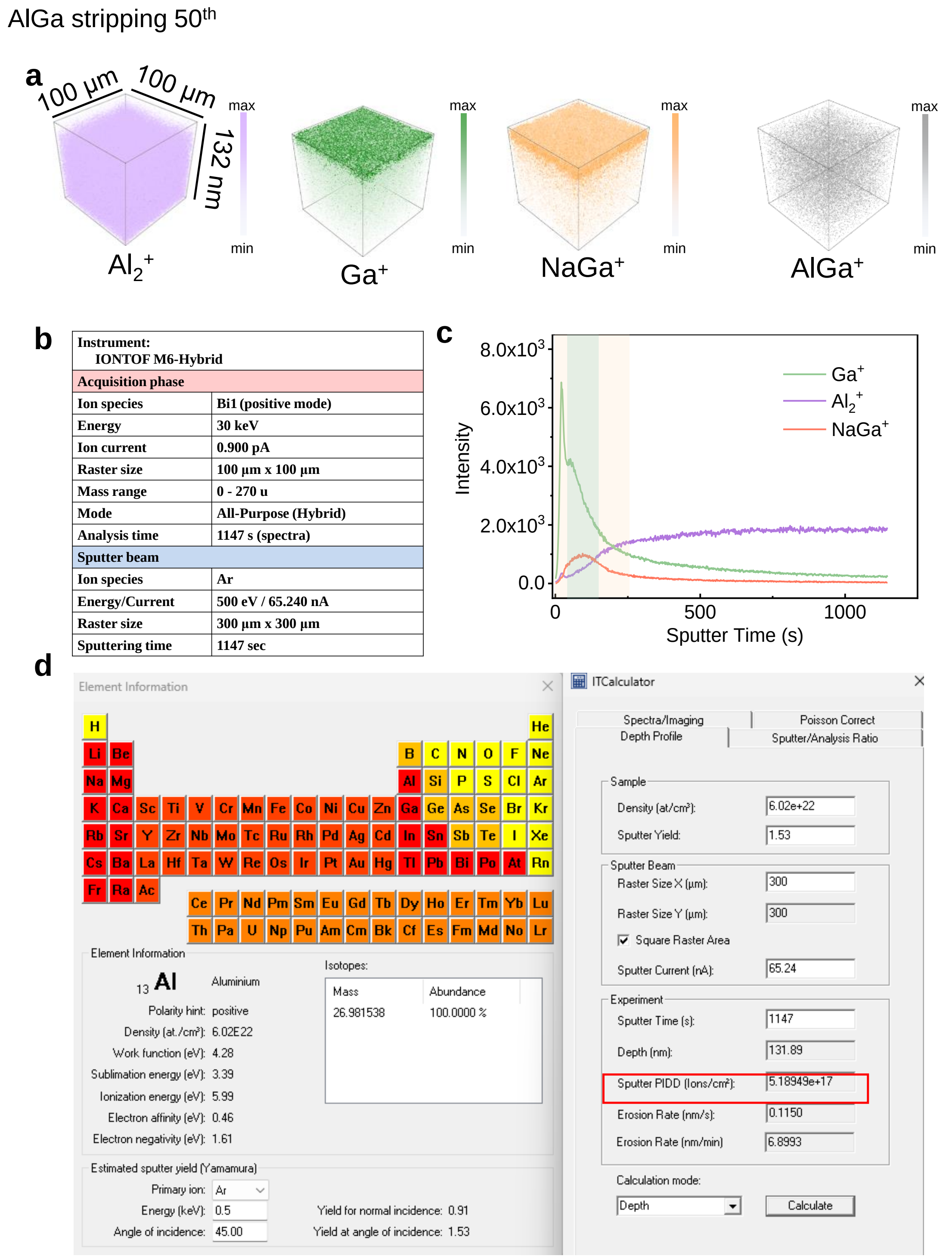

Supplementary Fig. 24 (a) TOF-SIMS depth profiles of AlGa after 50 cycles. (b) Actual conditions of TOF-SIMS testing. (c)The variation of fragment species intensity with sputtering time. (d) Calculation of the Al etching rate corresponding to the set conditions within the instrument. For metal Al, the etching rate is 0.1150 nm s^{-1}. At an etching time of approximately 250 s, there is no significant change in Ga^+ ions. Therefore, the thickness of the Ga layer is estimated to be approximately 9.3 nm (0.1150 × 250 = 28.8 nm).

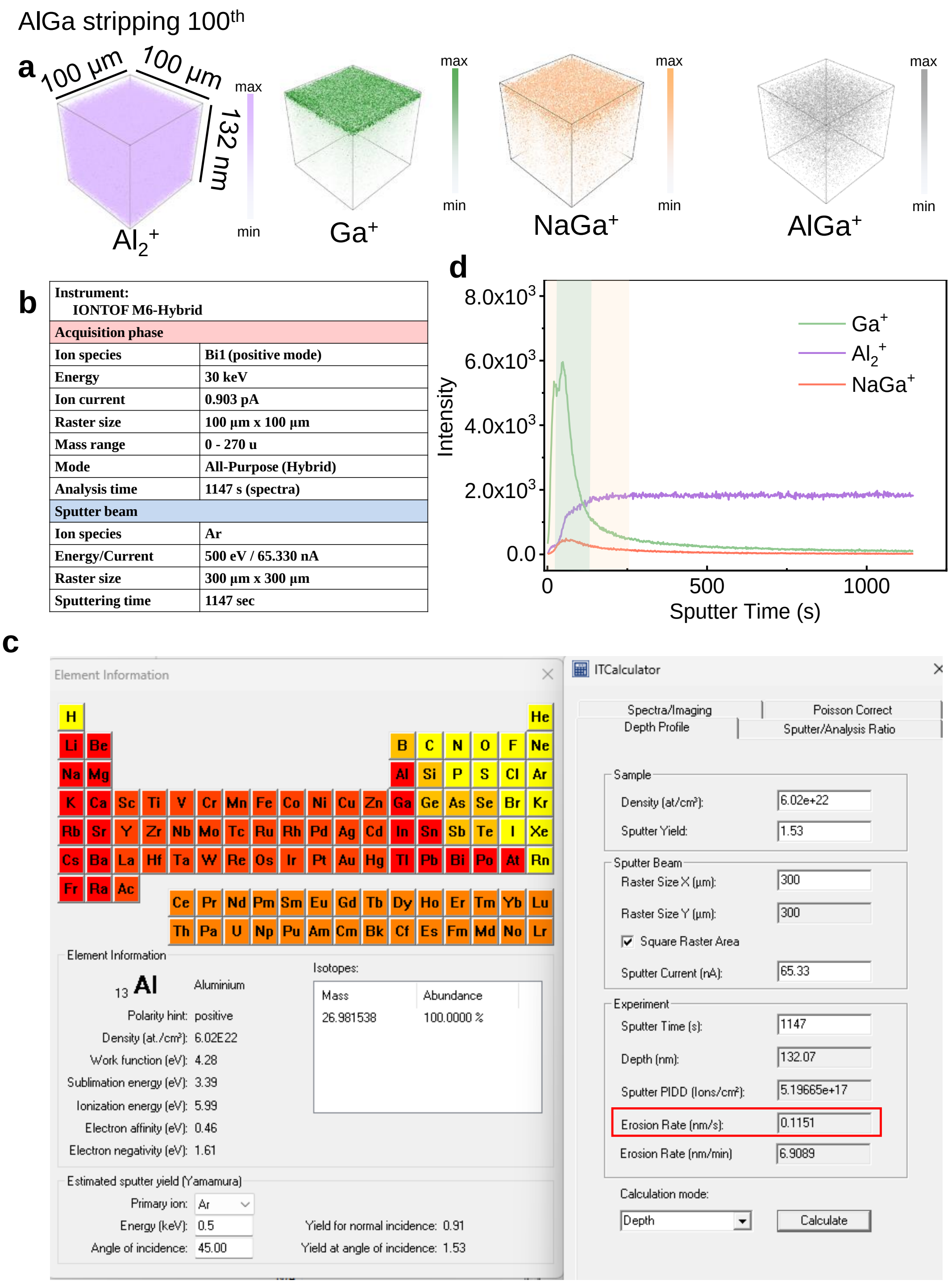

**Instrument:** **IONTOF M6-Hybrid**	
**Acquisition phase**	
**Ion species**	**Bi1 (positive mode)**
**Energy**	**30 keV**
**Ion current**	**0.903 pA**
**Raster size**	**100 μm x 100 μm**
**Mass range**	**0 - 270 u**
**Mode**	**All-Purpose (Hybrid)**
**Analysis time**	**1147 s (spectra)**
**Sputter beam**	
**Ion species**	**Ar**
**Energy/Current**	**500 eV / 65.330 nA**
**Raster size**	**300 μm x 300 μm**
**Sputtering time**	**1147 sec**

Supplementary Fig. 25 (a) TOF-SIMS depth profiles of AlGa after 100 cycles. (b) Actual conditions of TOF-SIMS testing. (c)The variation of fragment species intensity with sputtering time. (d) Calculation of the Al etching rate corresponding to the set conditions within the instrument. For metal Al, the etching rate is 0.1151 nm s^{-1}. At an etching time of approximately 250 s, there is no significant change in Ga^+ ions. Therefore, the thickness of the Ga layer is estimated to be approximately 28.8 nm $(0.1151 \times 250 = 28.8$ nm).

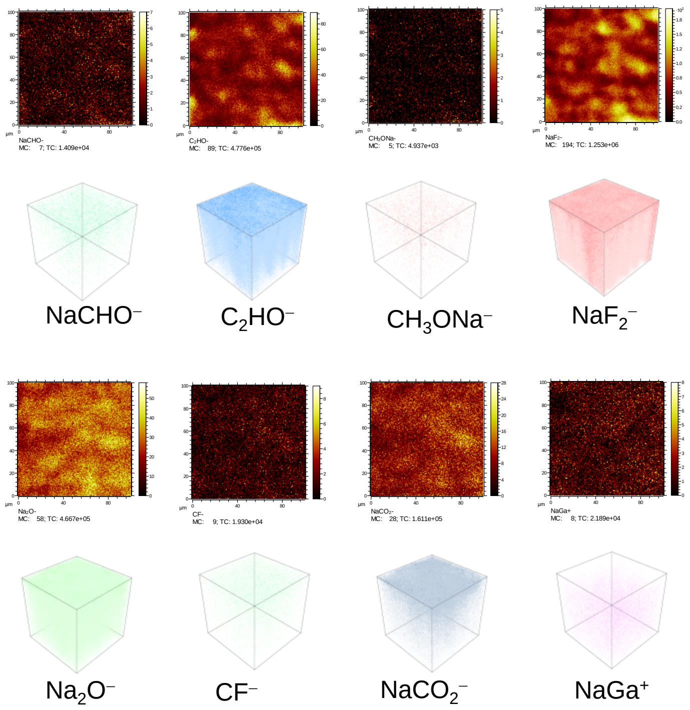

Instrument: IONTOF M6-Hybrid	
Acquisition phase	
Ion species	Bi3 (positive mode)
Energy	30 keV
Ion current	0.517 pA
Raster size	100 µm x 100 µm
pixel dimensions	128 x 128
Mass range	0 - 270 u
Mode	All-Purpose (Hybrid)
Analysis time	600 s (spectra)
Sputter beam	
Ion species	Ar
Energy/Current	500 eV / 65.020 nA
Raster size	300 µm x 300 µm
Sputtering time	600 sec

Instrument: IONTOF M6-Hybrid	
Acquisition phase	
Ion species	Bi3 (negative mode)
Energy	30 keV
Ion current	0.518 pA
Raster size	100 µm x 100 µm
pixel dimensions	128 x 128
Mass range	0 - 270 u
Mode	All-Purpose (Hybrid)
Analysis time	600 s (spectra)
Sputter beam	
Ion species	Ar
Energy/Current	500 eV / 64.560 nA
Raster size	300 µm x 300 µm
Sputtering time	600 sec

Supplementary Fig. 29 TOF-SIMS 3D reconstruction of the sputtered volume of several secondary ion fragments on the cycled AlGa surface.

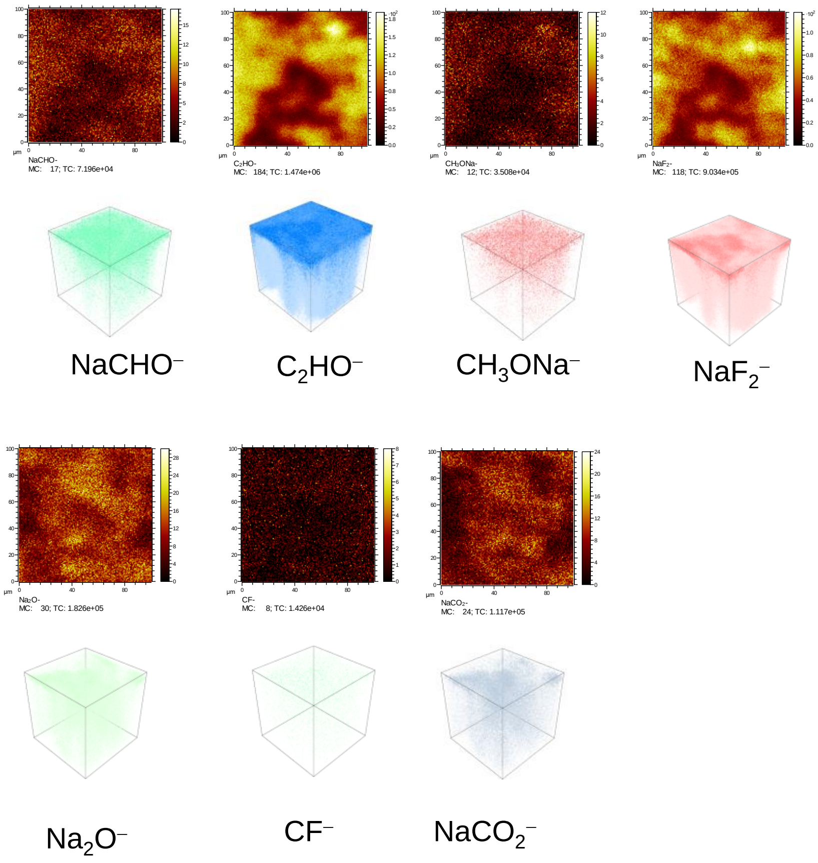

Instrument:<br>IONTOF M6-Hybrid	
Acquisition phase	
Ion species	Bi3 (positive mode)
Energy	30 keV
Ion current	0.511 pA
Raster size	100 μm x 100 μm
pixel dimensions	128 x 128
Mass range	0 - 270 u
Mode	All-Purpose (Hybrid)
Analysis time	600 s (spectra)
Sputter beam	
Ion species	Ar
Energy/Current	500 eV / 65.300 nA
Raster size	300 μm x 300 μm
Sputtering time	600 sec

Supplementary Fig. 30 TOF-SIMS 3D reconstruction of the sputtered volume of several secondary ion fragments on the cycled Al surface.

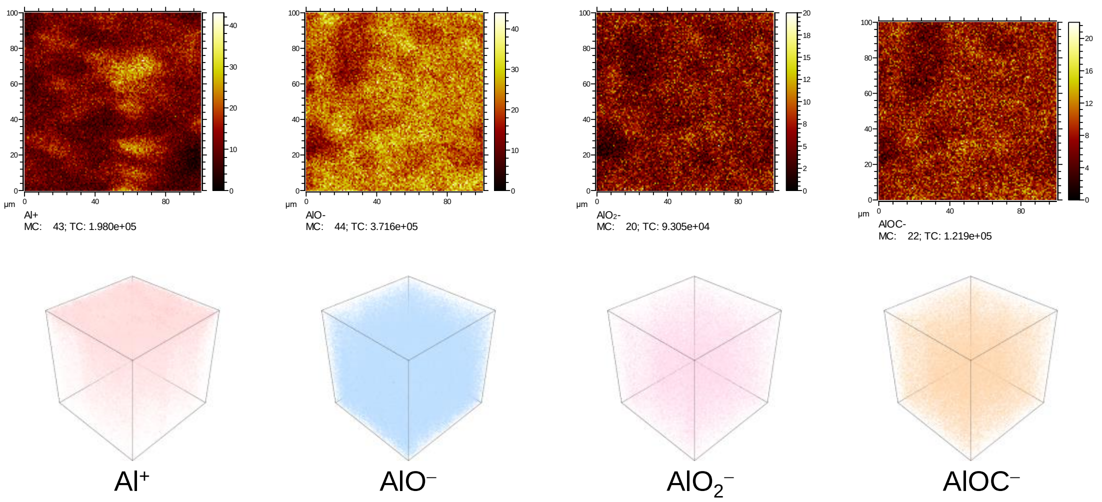

Instrument: IONTOF M6-Hybrid	
Acquisition phase	
Ion species	Bi3 (positive mode)
Energy	30 keV
Ion current	0.522 pA
Raster size	100 µm x 100 µm
Mass range	0 - 270 u
Mode	All-Purpose (Hybrid)
Analysis time	600 s (spectra)
Sputter beam	
Ion species	Ar
Energy/Current	500 eV / 62.12 nA
Raster size	300 µm x 300 µm
Sputtering time	600 sec

Instrument: IONTOF M6-Hybrid	
Acquisition phase	
Ion species	Bi3 (negative mode)
Energy	30 keV
Ion current	0.519 pA
Raster size	100 µm x 100 µm
Mass range	0 - 270 u
Mode	All-Purpose (Hybrid)
Analysis time	600 s (spectra)
Sputter beam	
Ion species	Ar
Energy/Current	500 eV / 62.460 nA
Raster size	300 µm x 300 µm
Sputtering time	600 sec

Supplementary Fig. 33 TOF-SIMS 3D reconstruction of the sputtered volume of several secondary ion fragments on the cycled NVP matched with AlGa surface.
